# Identification of QTNs Associated With Flowering Time, Maturity, and Plant Height Traits in *Linum usitatissimum* L. Using Genome-Wide Association Study

**DOI:** 10.3389/fgene.2022.811924

**Published:** 2022-06-14

**Authors:** Ankit Saroha, Deepa Pal, Sunil S. Gomashe, Akash, Vikender Kaur, Shraddha Ujjainwal, S. Rajkumar, J. Aravind, J. Radhamani, Rajesh Kumar, Dinesh Chand, Abhishek Sengupta, Dhammaprakash Pandhari Wankhede

**Affiliations:** ^1^ Division of Genomic Resources, Indian Council of Agricultural Research (ICAR)-National Bureau of Plant Genetic Resources, New Delhi, India; ^2^ Amity Institute of Biotechnology, Amity University, Noida, India; ^3^ ICAR-National Bureau of Plant Genetic Resources, Regional Station Akola, Maharashtra, India; ^4^ Division of Germplasm Evaluation, ICAR-National Bureau of Plant Genetic Resources, New Delhi, India; ^5^ Division of Germplasm Conservation, ICAR-National Bureau of Plant Genetic Resources, New Delhi, India

**Keywords:** flowering time, maturity, plant height, multi-locus GWAS, quantitative trait nucleotides, linseed, flaxseed

## Abstract

Early flowering, maturity, and plant height are important traits for linseed to fit in rice fallows, for rainfed agriculture, and for economically viable cultivation. Here, Multi-Locus Genome-Wide Association Study (ML-GWAS) was undertaken in an association mapping panel of 131 accessions, genotyped using 68,925 SNPs identified by genotyping by sequencing approach. Phenotypic evaluation data of five environments comprising 3 years and two locations were used. GWAS was performed for three flowering time traits including days to 5%, 50%, and 95% flowering, days to maturity, and plant height by employing five ML-GWAS methods: FASTmrEMMA, FASTmrMLM, ISIS EM-BLASSO, mrMLM, and pLARmEB. A total of 335 unique QTNs have been identified for five traits across five environments. 109 QTNs were stable as observed in ≥2 methods and/or environments, explaining up to 36.6% phenotypic variance. For three flowering time traits, days to maturity, and plant height, 53, 30, and 27 stable QTNs, respectively, were identified. Candidate genes having roles in flower, pollen, embryo, seed and fruit development, and xylem/phloem histogenesis have been identified. Gene expression of candidate genes for flowering and plant height were studied using transcriptome of an early maturing variety *Sharda* (IC0523807). The present study unravels QTNs/candidate genes underlying complex flowering, days to maturity, and plant height traits in linseed.

## Introduction

Linseed (*Linum usitatissimum* L.) is one of the earliest domesticated crops of the world and has been used for seed oil and fiber purposes since ancient times. It is a self-pollinated annual crop with a genome size of 373 Mb (2n = 2× = 30) (Wang et al., 2012). Linseed is considered to have originated in the central Asiatic center (northwest India), the near-eastern center, the Mediterranean center, and the Abyssinian Centre (Vavilov 1951). There are two morphotypes of linseed/flaxseed, flax type and linseed type, which differ substantially in terms of growth, development, and agronomic performance (Diederichsen and Ulrich, 2009; Soto-Cerda et al., 2013). For linseed type, short height, high branching, and high number of capsules and seed weight are desirable traits.

Linseed is one of the richest sources of omega-3 alpha linolenic acid (55–57%), which has tremendous cardiovascular benefits. Additionally, seeds of linseed have plenty of bioactive compounds such as lignans and soluble fibers, which are known for anticancer properties and reducing blood cholesterols, respectively (Green et al., 2008). In recent times, linseed is gaining popularity as a nutraceutical and functional food (Bassett et al., 2009; Goyal et al., 2014). India is one of the major linseed-producing countries (ranks 6th in the world) with a production of 174,000 tonne from 320,000 ha. area (FAOSTAT 2018). However, linseed productivity in India is far below (0.543 tonne/ha) the world average (1.053 tonne/ha) since it is mainly grown as a rainfed crop with limited or no additional resources such as irrigation and fertilizers (Kaur et al., 2017). High temperature during flowering even for a brief spell has significant negative impact on the seed set, which contributes to reduced seed yield (Ford and Zimmerman 1964; Wheeler et al., 2000; Jagadish et al., 2008). Early flowering and maturity are desirable traits for linseed cultivation in different countries (Miller et al., 2001; Hall et al., 2016; Sun et al., 2019) as it helps avoid frost, drought, and terminal heat and are suited for rainfed and Utera cultivation (Singh and Satapathy, 2019).

Flowering regulation in linseed is complex and affected by photoperiod as well as temperature. Linseed is a facultative long-day plant, and flowering initiation in short days is essential for the onset of the reproductive stage to facilitate early maturity (Domantovich et al., 2012). There also exist genotypic differences for photoperiod response (Zhang 2013; Sirohi and Wasnik 2018; Sun et al., 2019; Saroha et al., 2022). Genetic dissection of flowering time, maturity, and complex agro-morphological traits could enable tailoring locally adaptable high-yielding varieties in linseed.

For identifying genes/genomic regions underlying the complex traits, biparental quantitative trait loci (QTLs) and genome-wide association study (GWAS) are being used (Mackay et al., 2009; Burghardt et al., 2017). GWAS has the advantage over biparental QTL mapping on two fronts. The latter assays genetic variation limited to two parents and fewer recombination events. GWAS assays a wide swathe of natural variation through population-scale samples and also takes into account the historic recombination events across lineages, enabling a finer resolution of QTL (Burghardt et al., 2017). GWAS has been widely used in a wide range of plants such as rice, wheat, foxtail millet, chickpea, and greengram (Kumar et al., 2015; Jaiswal et al., 2019; Kumar et al., 2020; Reddy et al., 2020; Chaurasia et al., 2021). In flax, this approach was recently used for genetic dissection of fatty acid biosynthesis and agro-morphological traits (such as plant height, fiber percentage, 50% flowering, seed weight, and capsule and branch numbers), mucilage, and seed hull content (Xie et al., 2018a; Xie et al., 2018b, Singh et al., 2019; Soto-Cerda et al., 2018).

Advances in next-generation sequencing technologies have revolutionized research in agriculture and medical sciences. SNPs identified in the high-throughput manner have enabled association between phenotype and genotype (Wickland et al., 2017). Genotyping by Sequencing (GBS), a reduced representation sequencing approach (Hirsch et al., 2014), has become popular in agriculture owing to low cost and high-throughput genotyping of large number of accessions (Elshire et al., 2011; Wickland et al., 2017). GBS has been employed successfully in several crops such as barley, chickpea, and greengram (Kujur et al., 2015; Milner et al., 2019; Reddy et al., 2020).

In linseed/flaxseed, 340 QTLs have been identified by making use of SSR or SNP markers for 31 traits including 24 seed yield, seed quality, and agronomic traits (You and Cloutier, 2020; Soto-Cerda et al., 2021). For days to flowering, days to maturity, and plant height, there are a total of 28, 2, and 30 known QTLs, respectively, from previous studies (Soto-Cerda et al., 2014, 2021; Wu et al., 2018; You et al., 2018; You and Cloutier, 2020). Most of these studies have been undertaken on the Canadian flax core collection (Diederichsen et al., 2013; Soto-Cerda et al., 2014; 2021), or core collections from China and other countries (Xie et al., 2018a; Xie et al., 2018b) which have no or limited representation of linseed genetic resources from the Indian sub-continent. The natural genetic variation underlying the linseed accessions of the National Gene Bank (NGB), India, is yet to be tapped and utilized for dissection of complex agro-morphologically important traits. The present study was conducted with the aim of genetic dissection of flowering time, days to maturity, and plant height using genome-wide association strategy in a diverse panel of linseed accessions from NGB, India.

## Materials and Methods

### Plant Materials, Field Evaluation, and Statistical Analysis

A total of 220 diverse linseed germplasm accessions (53 exotic collections and 167 indigenous collections) from NGB, Indian Council of Agricultural Research-National Bureau of Plant Genetic Resources, were evaluated at New Delhi (28°38′53.7″N 77°09′05.4″E) for 2 years, 2017–18 (DL17-18) and 2018–19 (DL18-19), as reported earlier by our group (Saroha et al., 2022). The same set was also evaluated in three more environments including 2019–20 at New Delhi (DL19-20) and at Akola (20°42′03.2″N 77°01′53.6″E) for 2 years, 2018–19 (AK18-19) and 2019–20 (AK19-20). The evaluation was carried out following Augmented Block Design (ABD) in six blocks with three checks (T-397, Shekhar, and Kartika), replicating twice in each block. Each accession was grown in 3-m single rows with a distance of 45 cm between two rows. ICAR-NBPGR descriptors (Mahajan et al., 2000) were followed for recording the agro-morphological data. Plant height (PH) was recorded on three randomly selected plants from the middle of the row. For flowering time (days to 5% flowering: DF5, days to 50% flowering: DF50, and days to 95% flowering: DF95) and days to maturity (days to 80% physiological maturity: DM) traits, the entire row/accession was considered for recording the observations. Data of each individual environment was analyzed using augmentedRCBD, R package version 0.1.5 (Aravind et al., 2021), following which the adjusted means were calculated. An association mapping (AM) panel of 131 diverse accessions was constituted following standard procedure to include maximum trait variation for the studied traits. For descriptive statistics, adjusted means of selected 131 accessions were filtered from a total of 220 accessions. Descriptive statistics for the AM panel were calculated using PAST software (v4.04) (Hammer et al., 2001). The AM panel consisted of 85 indigenous accessions and 46 exotic accessions (Supplementary Table S1).

### Genotyping, Population Structure, and Linkage Disequilibrium

Genomic DNA was isolated from two-week-old seedlings grown from seeds of the single plant progeny of each accession using a DNeasy Plant Mini Kit (QIAGEN). The ApeKI-digested, adapter-ligated, amplified, and purified 131-plex final DNA library was quantified using a Bioanalyzer (Agilent Technologies) and were sequenced on a single lane of the Illumina HiSeqTM X10 platform (Illumina^®^ Inc., San Diego, CA, United States). Cleaned reads were mapped to reference genome *Linum usitatissimum* (You et al., 2018) downloaded from NCBI (Pseudomolecule level) using the MEM algorithm of BWA (v0.7.5) (Li, 2013). Variant calling was done using the GATK pipeline (v3.6), (Geraldine and Brian, 2020). Variants were filtered and indels were removed using vcftools (v0.1.17) (Danecek et al., 2011), keeping only biallelic SNPs. For excluding the rare alleles which may also arise due to genotyping errors, all SNPs were filtered at read-depth 10, stringent MAF of 8%, and missing data <20%. Population structure was estimated using an admixture-based model in STRUCTURE software (v2.3.4) (Pritchard et al., 2000). Three runs were performed for each number of the population (K) set from 3 to 9. Burn-in time and MCMC replication number were set to 100,000 and 300,000, respectively, for each run. The most probable K-value was determined using Structure Harvester, using the log probability of the data [LnP(D)] and delta K (ΔK) based on the rate of change in [LnP(D)] between successive K-values. PCA was calculated using PLINK (version 1.9) and then plotted using ‘R.’ The dendrogram was constructed using TASSEL (v4.0) (Bradbury et al., 2007) with the Neighbour-Joining method and then plotted with the Structure Q matrix using iTOL (Letunic and Bork, 2021). Genome-wide LD was estimated using the squared correlations of allele frequency (*r* ^2^), using TASSEL (v5.2.73) with a sliding window size of 50. LD decay distance for the genome was estimated by plotting the scatterplot of LD *r* ^2^ values between marker pairs and physical distance.

### Genome-Wide Association Study (GWAS)

Six multi-locus models, FASTmrEMMA (Wen et al., 2018), FASTmrMLM (Zhang and Tamba, 2018), ISIS EM-BLASSO (Tamba et al., 2017), mrMLM (Wang et al., 2016), pLARmEB (Zhang et al., 2017), and pKWmEB (Ren et al., 2018) implemented in the mrMLM package v4.0.2 (Zhang et al., 2020) of ‘R,’ have been used in this study. Default values were used on all the parameters for the analysis. SNP genotyping information of the AM panel of 131 accessions and phenotyping information of five traits (DF5, DF50, DF95, DM, and PH) were used for the five individual environments independently for GWAS. QTNs with a threshold of Logarithm of Odds (LOD) score ≥3.0 were considered to be significantly associated with the trait. Manhattan and QQ plots were generated using the mrMLM package v4.0.2 of ‘R’ (Zhang et al., 2020).

### Identification of Candidate Genes and Gene Expression Study

Genes around the 30 kb sequence (30 kb upstream and downstream, total 60 kb) of the stable QTNs were extracted following the flax genome annotation (You et al., 2018; You and Cloutier, 2020). Putative candidate genes were filtered based on functional annotation and homology with the Arabidopsis ortholog. Functional annotation of the genes was performed using the PANNZER2 tool (Törönen and Holm, 2022).

Differential gene expression of the candidate genes for flowering time (DF5, DF50, and DF95) and PH was studied using transcriptome (BioProject ID: PRJNA773597) of the floral bud at two developmental stages, flower, leaf, and stem of the early flowering variety *Sharda* (IC0523807). Reads per kilobase of transcript per million fragments mapped (RPKM) values were estimated to get the expression levels of candidate genes. For DM, expression of the candidate genes was performed *in silico*. Gene expression data and protein sequences of the rice variety *Nipponbare* were downloaded from the Rice Genome Annotation Project database (http://rice.uga.edu/index.shtml). Putative candidate genes of the DM trait were aligned against the protein sequences of *Nopponbare*, and the best hit was considered for expression analysis. Expression analysis was performed for the following developmental stages: Embryo—25 Days After Pollination (DAP) (SRX100753), Endosperm—25 DAP (SRX100754) and Seed—5 DAP (SRX100749) and 10 DAP (SRX100755). Heatmap plots of gene expression were generated using the ComplexHeatmap package v2.10.0 (Gu et al., 2016) of ‘R.’

## Results

### Phenotypic Variation

**FIGURE 1:**
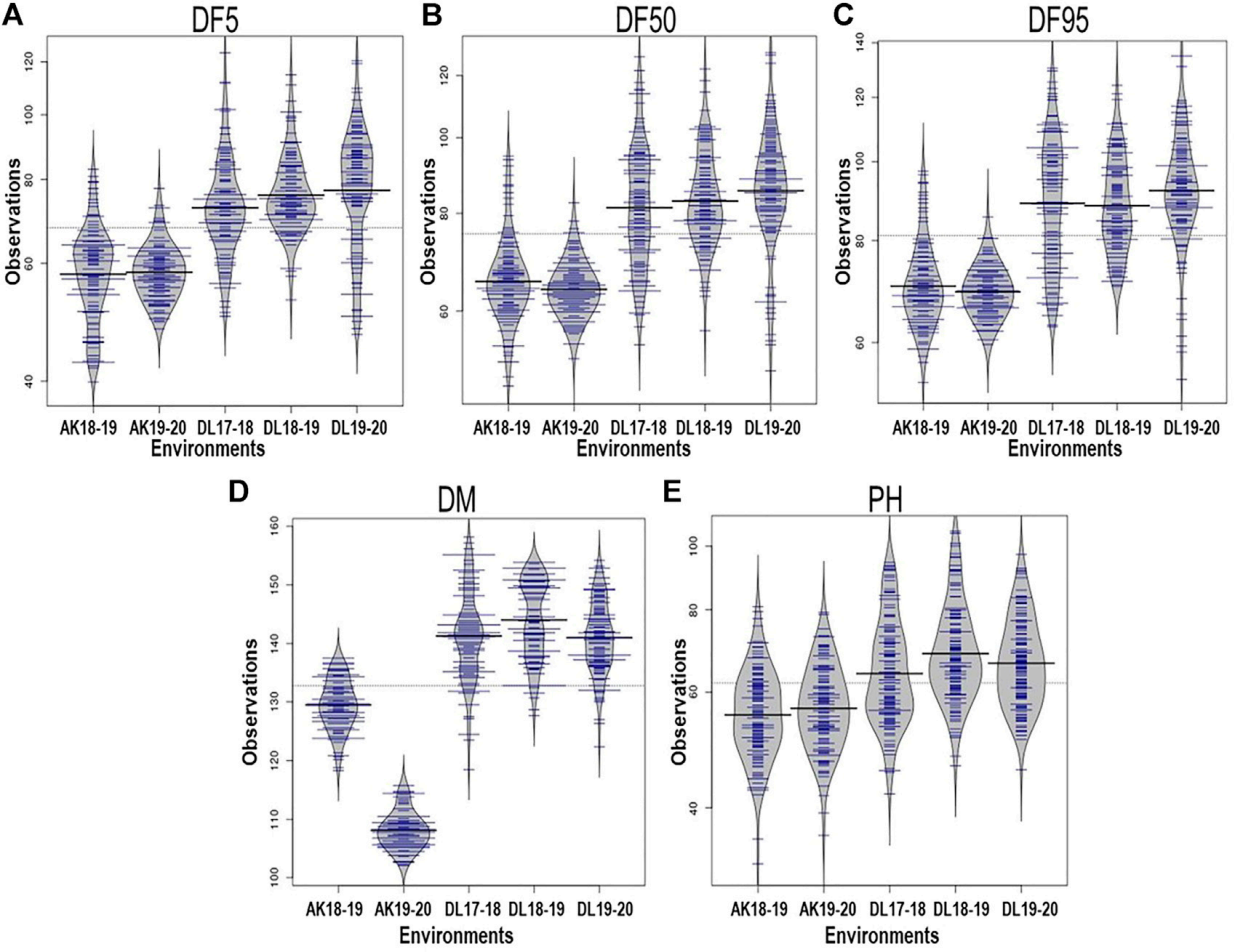
Phenotypic variation depicted in bean plots in the association mapping panel of linseed accession for days to 5% flowering, DF5 **(A)**; 50%, DF50 **(B)**; 95%, DF95 **(C)**; days to maturity, DM **(D);** and plant height, PH **(E)** in five environments. AK and DL stand for geographical locations Akola and New Delhi, respectively, and the following figures indicate years. The length of the bean depicts extent of variation and width along with blue lines depict relative number of accessions representing corresponding values on the Y-axis. The horizontal black lines represent the mean value.

All the studied traits, DF5, DF50, DF95, DM, and PH, were heterogeneous for error variances; therefore, analysis for the respective environments was done independently. The extent of variation among the germplasm accessions was very high for flowering time traits. Out of the five environments, the lowest value for flowering time initiation (DF5) was 39.86 days, whereas the highest value was 123.81 days, with a maximum coefficient of variation (CV) of 19.23 (Table 1). For DF50, the minimum and maximum values were 48.14 and 128.5 days, respectively, with a maximum CV of 17.96. For completion of flowering (DF95), 53.56 and 134.89 days were minimum and maximum, respectively, with the highest CV being 18.07. In case of days to maturity, the extent of variation was relatively less than that of the flowering time traits. The minimum and maximum days to attain the physiological maturity were 102.11 and 158.17, respectively, with a maximum CV of 5.41. For the plant height trait, the range was 32.90–105.23 cm, with a maximum CV of 18.71 from all five environments. The variation among the accessions for the individual environment was also very high (Table 1; Figure 1) For all five traits, there were conspicuous differences in trait expression between the Akola and New Delhi regions, the former being the 2^nd^ and the latter the 3^rd^ zone of linseed growing areas of India (Figure 1). Linseed accessions showed earlier flowering, fewer days to maturity, and relatively shorter height in Akola than in New Delhi. Overall, for the AM panel, analysis of variance in all five environments showed significant variation for all five traits (Supplementary Table S2).

**TABLE 1:** Descriptive statistics of association mapping panel of 131 accessions for flowering time, days to maturity, and plant height in five environments.

Trait	Environment	AK1819	AK1920	DL1718	DL1819	DL1920
DF5	Range	39.86–82.86	47.89–77.56	49.97–123.81	52.92–114.75	46.97–120.31
	Mean	58.54	58.46	73.64	76.45	78.60
	Standard deviation	9.14	5.55	12.75	10.39	15.11
	Coefficient variation	15.62	9.49	17.31	13.59	19.23
	Standard error	0.80	0.48	1.11	0.91	1.32
DF50	Range	48.14–94.64	52.17–82.50	54.31–126.81	56.67–122.33	50.33–128.50
	Mean	65.97	64.17	82.63	83.73	86.69
	Standard deviation	8.46	5.06	14.84	11.67	14.15
	Coefficient variation	12.82	7.88	17.96	13.93	16.32
	Standard error	0.74	0.44	1.30	1.02	1.24
DF95	Range	53.56–97.39	59.67–85.50	62.75–130.25	70.33–124.17	54.06–134.89
	Mean	70.85	69.43	90.41	89.07	93.26
	Standard deviation	8.49	4.66	16.34	11.88	14.15
	Coefficient variation	11.99	6.71	18.07	13.34	15.17
	Standard error	0.74	0.41	1.43	1.04	1.24
DM	Range	118.31–137.47	102.11–115.78	118.50–158.17	127.67–153.83	122.33–154.17
	Mean	129.51	108.12	141.27	144.02	140.98
	Standard deviation	4.32	3.15	7.64	6.84	6.12
	Coefficient variation	3.33	2.91	5.41	4.75	4.34
	Standard error	0.38	0.27	0.67	0.60	0.53
PH	Range	32.90–80.90	36.33–79.17	42.01–94.34	46.40–105.23	45.73–97.15
	Mean	56.01	57.24	65.04	69.58	67.16
	Standard deviation	8.64	8.31	12.17	12.15	10.53
	Coefficient variation	15.43	14.52	18.71	17.47	15.68
	Standard error	0.75	0.73	1.06	1.06	0.92

### Genome-Wide Identification of SNPs by Genotyping by Sequencing Approach

**FIGURE 2:**
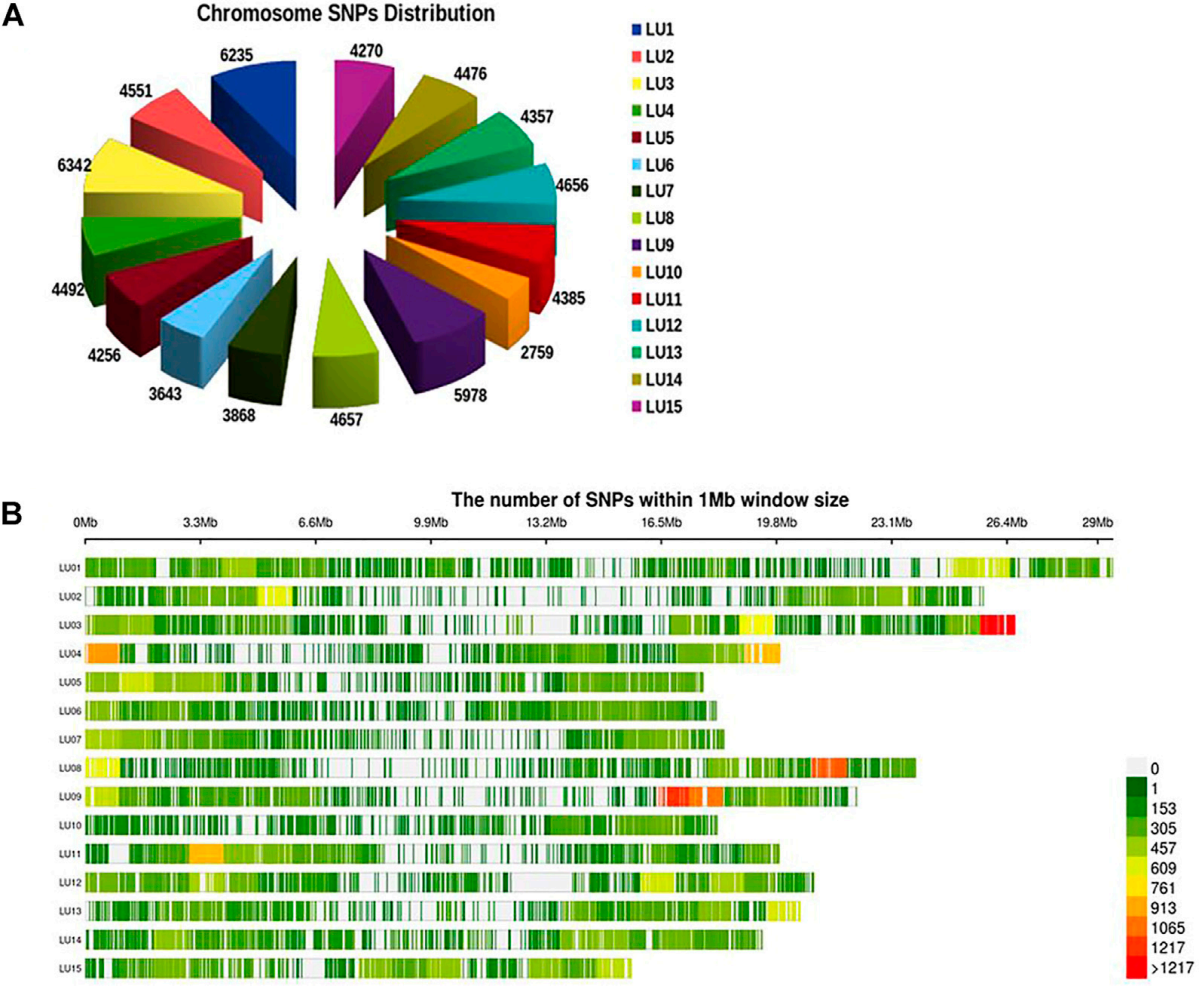
Distribution of SNPs on 15 chromosomes of linseed **(A)** and density of SNPs across individual chromosomes **(B)**.

**FIGURE 3:**
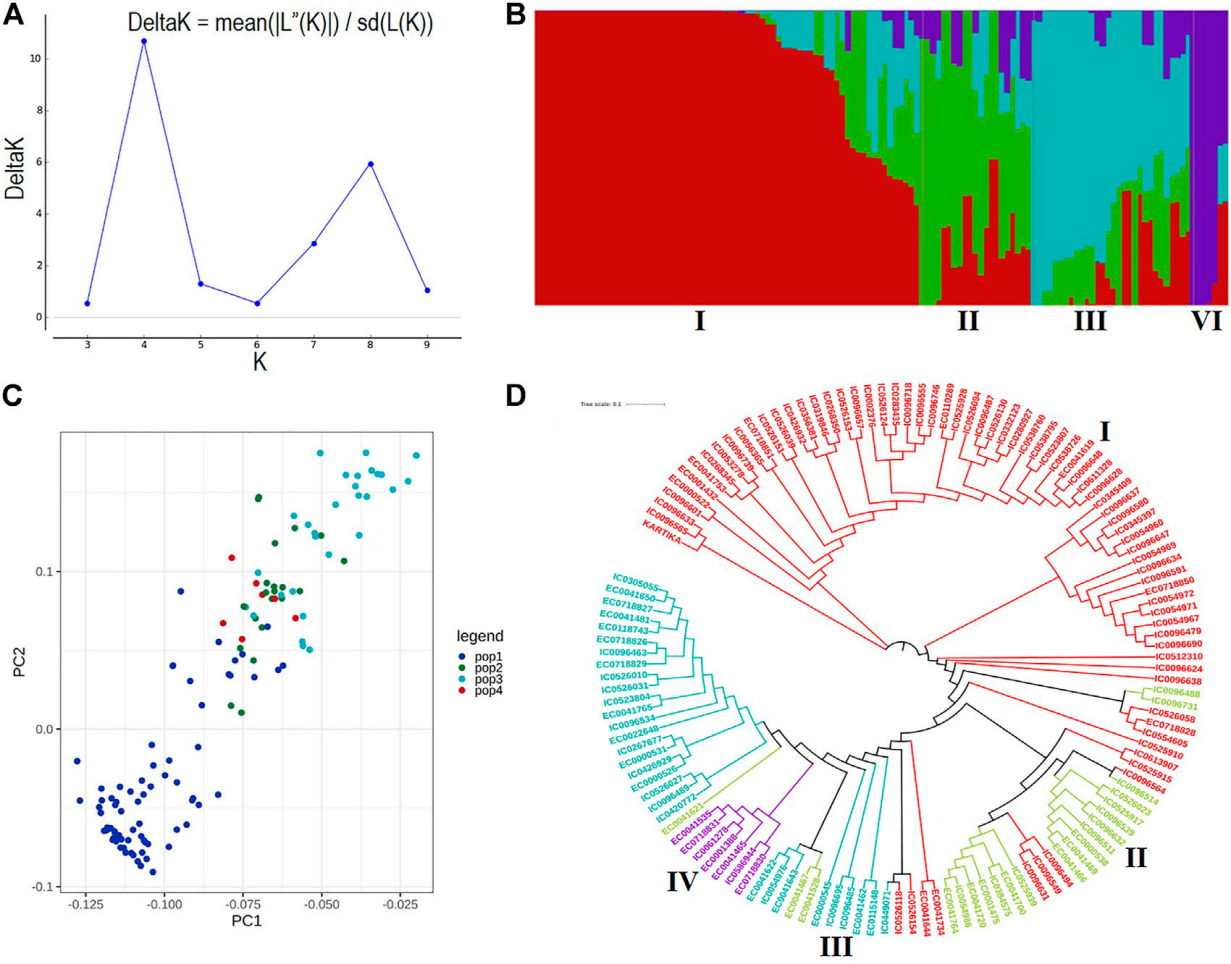
**(A)** ΔK plot showing best peak at K = 4. **(B)** Population structure of the linseed association mapping panel showing 4 sub-populations indicated by distinct colors. **(C)** Principal component analysis plot of the first two components showing 131 accessions into four clusters. **(D)** Dendrogram based on the Neighbour-Joining method. Accessions with different colors belong to respective sub-populations as shown in population structure.

**FIGURE 4:**
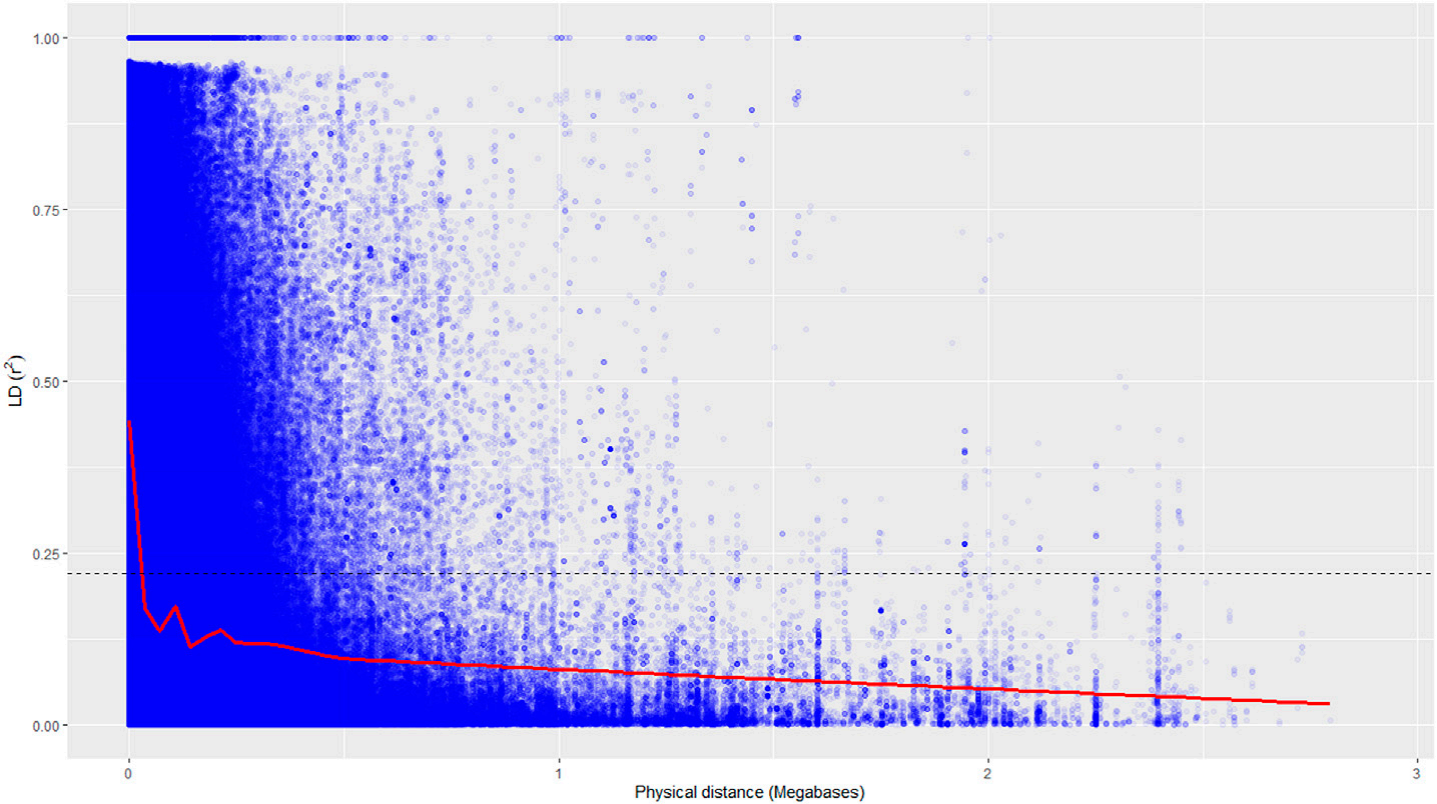
Genome-wide linkage disequilibrium decay of *r* ^*2*^ values (red line) against the physical distance in a panel of 131 accessions. The dotted line indicates a cut-off value of 50%.

A total of 19.5 million quality reads were obtained by sequencing of ApeKI digested GBS libraries of 131 linseed germplasm accessions. A total of 68,925 high-quality SNPs were obtained with a read depth of 10, <20% missing data, and 8% minor allele frequency, which were mapped on the 15 chromosomes. The highest number of SNPs, 6,342, were mapped on chromosome 3, and the lowest number, 2,759, were mapped on chromosome 10 (Figure 2A). The distribution of SNPs across 15 chromosomes is shown in Figure 2B. To understand the pattern of the population structure in the AM panel of linseed accessions, 68,925 SNPs were employed using an admixture-based model in STRUCTURE software. The number of distinct sub-populations in the AM panel was determined as 4 per the maximum Delta K value plotted against the K using the ‘Structure Harvester’ program (Figure 3A). The bar plot clearly shows four sub-populations (Figure 3B). Sub-population-I consists of a maximum of 73 accessions of which 10 were exotic (EC) and 63 were indigenous (IC). Sub-population-II showed 21 accessions (IC: 11, EC: 10), sub-population-III: 30 accessions (IC: 15, EC: 15), and sub-population-IV showed only 7 accessions (IC: 2, EC: 5). In principal component analysis (PCA), plotting of the first two components against each other shows 4 clusters (Figure 3C). Furthermore, the phylogenetic tree using 68,925 SNPs grouped 131 accessions into four major clusters (Figure 3D). The estimates of *r* ^*2*^ for 68,925 SNP loci were used to assess the rate of LD decay with distance. The genome-wide LD declined to 50% of its initial value at about 30 kb (Figure 4).

### Genome-Wide Association Study

Using five ML-GWAS methods, a total of 620 significant QTNs (LOD score of ≥3.0) have been identified for five traits across five environments, of which 335 were unique QTNs. With respect to methods, 26, 94, 144, 115, and 149 QTNs were identified using FASTmrEMMA, FASTmrMLM, ISIS EM-BLASSO, mrMLM, and pLARmEB, respectively; however, there were some common QTNs across methods (Supplementary Table S3). The multi-locus method, pKWmEB, could not identify any significant QTN in any of the environments. Among the significant QTNs, 68, 80, and 76 were for DF5, DF50, and DF95, respectively, whereas 84 and 95 QTNs were identified for DM and PH, respectively (Supplementary Table S3). In order to select the robust QTNs, the QTNs which have been identified either using ≥ two methods and/or in ≥ two environments were considered stable QTNs. From the stable QTNs, those identified in ≥ two methods or in ≥ two environments were considered strong QTNs, while those identified in ≥ two methods along with ≥ two environments were considered very strong QTNs. Accordingly, 21 very strong and 88 strong unique QTNs have been identified for the five traits (Supplementary Table S3). The rest of the significant QTNs were considered as potential QTNs. The total number of significant QTNs for all the studied traits in five environments using five ML-GWAS methods is shown (Supplementary Table S4).

In DF5, there were 21 stable QTNs, of which 5 and 16 were very strong and strong, respectively (Table 2). For DF50, a total of 28 stable QTNs comprised 4 very strong and 24 strong QTNs (Table 3), whereas for DF95, from 24 stable QTNs, 2 were very strong and 22 were strong QTNs (Table 4). Moreover, there were 10, 13, and 12 QTNs for DF5, DF50, and DF95, respectively, which were identified in ≥3 methods and/or environments. From the total of 73 stable QTNs for three flowering time traits, 15 QTNs were co-identified either in all or two of the three flowering time traits. One QTN (Lu07_3538758) showed pleiotropic effect and was identified for DF50 as well as DM. For three flowering time traits, the range of the LOD score and corresponding −log10(*p*) value ranged from 3.0 to 13.76 and 3.70 to 14.77, respectively, explaining up to 35.28% of flowering time variation (Tables 2−4).

**TABLE 2:** Quantitative trait nucleotides (QTNs) identified for days to 5% flowering (DF5).

QTN	Allele	Physical position (bp)	LOD score	−log10(*p*)	*r* ^*2*^ (%)	MAF	Environments (methods^a^)
**Lu01_17201820**	**G/A**	**Lu01, 17201820**	**3.01–6.88**	**3.71–7.74**	**6.15–19.21**	**0.19**	**AK19–20 (4); DL18–19 (5)**
Lu02_16065021	G/T	Lu02, 16065021	3.72–3.97	4.45–4.72	6.33–12.60	0.27	DL18–19 (4, 3)
Lu03_715991	T/G	Lu03, 715991	4.66–8.16	5.44–9.05	5.16–6.98	0.49	AK19–20 (4, 5)
Lu03_14655958	G/T	Lu03, 14655958	5.25–6.20	6.06–7.04	23.25–28.27	0.25	DL19–20 (2, 5)
Lu03_19174892	T/G	Lu03, 19174892	3.85–5.45	4.59–6.26	16.64–16.71	0.25	DL19–20 (4, 3)
Lu03_19423426	A/G	Lu03, 19423426	6.27–9.86	7.11–10.80	9.96–18.78	0.25	AK19–20 (4, 2, 5, 3)
Lu05_16229944	T/G	Lu05, 16229944	3.22–5.26	3.93–6.07	3.15–7.88	0.26	AK19–20 (4, 2, 5, 3)
Lu07_4061268	T/C	Lu07, 4061268	3.10–5.06	3.80–5.86	1.24–4.01	0.49	AK19–20 (4, 2, 5, 3)
Lu08_31911	A/G	Lu08, 31911	3.00–3.51	3.70–4.24	1.62–6.28	0.32	DL17–18 (1, 5, 3)
**Lu08_2981314**	**C/T**	**Lu08, 2981314**	**3.59–5.84**	**4.32–6.67**	**6.78–17.31**	**0.24**	**AK18**–**19 (2, 5, 3); DL18**–**19 (5)**
Lu09_16962441	A/G	Lu09, 16962441	3.29–3.86	4.00–4.60	6.62–8.93	0.41	DL19–20 (2, 3)
Lu09_21344639	A/G	Lu09, 21344639	4.86–6.16	5.65–7.00	10.82–15.80	0.29	AK19–20 (4, 2, 5, 3)
Lu10_15001635	G/A	Lu10, 15001635	3.75–5.65	4.49–6.47	5.96–11.55	0.46	DL17–18 (4, 5)
**Lu11_1592089**	**T/G**	**Lu11, 1592089**	**3.52–7.35**	**4.25–8.22**	**10.17–35.28**	**0.22**	**AK18**–**19 (4, 2, 5, 3); DL17-18 (4, 5, 3); DL19-20 (3)**
**Lu11_3283122**	**C/A**	**Lu11, 3283122**	**3.40–7.10**	**4.12–7.97**	**4.78–10.93**	**0.49**	**AK18**–**19 (4, 2, 5); DL17**–**18 (5); DL18**–**19 (5)**
Lu11_19845992	C/T	Lu11, 19845992	4.28–4.86	5.05–5.65	4.89–8.00	0.48	DL19–20 (2, 5)
**Lu12_2201590**	**T/C**	**Lu12, 2201590**	**3.46–7.98**	**4.19–8.87**	**10.47–15.29**	**0.42**	**AK18**–**19 (4, 2, 5); DL18**–**19 (4, 2, 3)**
Lu13_15811472	A/G	Lu13, 15811472	3.77–4.05	4.51–4.81	7.08–18.26	0.16	DL19–20 (4, 3)
Lu15_6199067	T/C	Lu15, 6199067	3.89–5.17	4.63–5.97	16.01–19.39	0.28	DL18–19 (4, 3)
Lu15_12894145	T/C	Lu15, 12894145	3.49–6.84	4.22–7.70	3.76–4.68	0.31	DL18–19 (2, 3)
Lu15_15418733	G/T	Lu15, 15418733	5.73–7.52	6.56–8.40	6.98–9.61	0.42	AK19–20 (4, 5, 3)

a Methods: FASTmrEMMA (1), FASTmrMLM (2), ISIS EM-BLASSO (3), mrMLM (4), pLARmEB (5), QTNs, with normal and bold fonts are designated as strong and very strong QTNs, respectively. Underlined QTNs, indicates that it was also identified in DF50 and/or DF95 traits.

**TABLE 3:** Quantitative trait nucleotides (QTNs) identified for days to 50% flowering (DF50).

QTN	Allele	Physical position (bp)	LOD score	−log10(*p*)	*r* ^*2*^ (%)	MAF	Environments (methods^a^)
Lu01_5208623	C/G	Lu01, 5208623	4.12–5.07	4.88–5.87	6.24–9.97	0.43	DL17–18 (4, 5, 3)
Lu01_10588059	T/C	Lu01, 10588059	3.47–4.74	4.19–5.53	6.24–11.19	0.44	DL19–20 (2, 5, 3)
Lu01_17201820	G/A	Lu01, 17201820	4.67–5.14	5.46–5.94	14.61–20.59	0.19	DL18–19 (2, 3)
Lu01_21634883	C/T	Lu01, 21634883	3.33–3.96	4.04–4.71	0.00–6.64	0.38	AK19–20 (1); DL19–20 (1)
**Lu01_27680796**	**G/A**	**Lu01, 27680796**	**3.15–4.42**	**3.85–5.20**	**4.86–17.71**	**0.48**	**AK18**–**19 (4); AK19**–**20 (3); DL18-19 (4)**
**Lu01_27777416**	**T/G**	**Lu01, 27777416**	**3.39–4.21**	**4.11–4.97**	**3.02–6.63**	**0.42**	**AK18**–**19 (5); AK19**–**20 (3)**
Lu02_22483108	G/C	Lu02, 22483108	6.12–7.77	6.96–8.65	6.48–6.49	0.38	AK19–20 (4, 2)
Lu03_4228242	T/C	Lu03, 4228242	4.64–5.80	5.42–6.63	2.93–3.81	0.38	AK19–20 (4, 2)
Lu03_9215257	G/A	Lu03, 9215257	3.99–4.88	4.75–5.67	10.27–14.62	0.21	DL17–18 (4, 5)
Lu03_19174892	T/G	Lu03, 19174892	3.48–4.87	4.20–5.66	8.96–24.67	0.25	DL19–20 (4, 2, 3)
**Lu03_19423426**	**A/G**	**Lu03, 19423426**	**3.29–13.76**	**4.00–14.77**	**4.33–27.65**	**0.25**	**AK18**–**19 (5); AK19**–**20 (4, 2, 5, 3)**
Lu03_24924140	T/C	Lu03, 24924140	3.35–3.97	4.07–4.72	5.32–9.16	0.30	DL17–18 (1, 5)
Lu05_7581214	T/C	Lu05, 7581214	3.59–3.88	4.33–4.63	5.68–8.33	0.32	DL17–18 (4, 5)
Lu05_14830471	C/G	Lu05, 14830471	3.45–3.71	4.17–4.45	5.13–6.79	0.32	AK19–20 (4, 3)
Lu05_15328362	G/T	Lu05, 15328362	6.26–10.44	7.11–11.39	7.65–10.49	0.47	AK19–20 (4, 2, 1, 3)
Lu06_5661970	C/G	Lu06, 5661970	3.25–5.24	3.96–6.04	4.98–6.95	0.27	AK19–20 (4, 2, 3)
Lu07_221399	A/G	Lu07, 221399	4.24–5.10	5.00–5.90	8.40–14.46	0.50	AK18–19 (4, 3)
Lu07_3538758	T/A	Lu07, 3538758	4.78–6.19	5.56–7.03	5.88–12.60	0.20	AK19–20 (4, 2, 5)
Lu07_3681565	G/T	Lu07, 3681565	3.77–6.98	4.51–7.85	5.55–12.37	0.32	DL17–18 (4, 2, 5)
Lu08_82326	A/T	Lu08, 82326	4.33–5.66	5.10–6.48	4.34–5.51	0.42	AK19–20 (4, 3)
Lu09_1801164	A/G	Lu09, 1801164	3.94–5.87	4.69–6.70	5.24–9.80	0.46	DL17–18 (4, 2, 5, 3)
Lu09_16714871	T/C	Lu09, 16714871	3.68–4.29	4.41–5.06	5.13–7.32	0.45	DL17–18 (4, 5)
Lu09_17305499	C/T	Lu09, 17305499	3.69–4.21	4.43–4.97	6.18–12.16	0.25	DL17–18 (4, 5)
Lu11_1592089	T/G	Lu11, 1592089	5.36–9.07	6.17–9.99	24.55–33.49	0.22	DL19–20 (4, 2, 3)
Lu11_3283122	C/A	Lu11, 3283122	3.25–4.29	3.96–5.06	5.14–8.95	0.49	AK18–19 (4, 3)
Lu12_4359290	G/A	Lu12, 4359290	3.33–3.99	4.05–4.74	9.52–20.37	0.28	AK18–19 (2); DL18–19 (2)
**Lu15_6199067**	**T/C**	**Lu15, 6199067**	**4.42–7.28**	**5.19–8.15**	**18.89–28.69**	**0.28**	**DL18**–**19 (2, 5, 3); DL19**–**20 (5)**
Lu15_15418733	G/T	Lu15, 15418733	3.38–8.90	4.10–9.82	4.92–12.23	0.42	AK19–20 (4, 2, 5, 3)

a Methods: FASTmrEMMA (1), FASTmrMLM (2), ISIS EM-BLASSO (3), mrMLM (4), pLARmEB (5). QTNs, with normal and bold fonts are designated as strong and very strong QTNs, respectively. Underlined QTNs, indicates that it was also identified in DF5 and/or DF95 traits.

**TABLE 4:** Quantitative trait nucleotides (QTNs) identified for days to 95% flowering (DF95).

QTN	Allele	Physical position (bp)	LOD score	−log10(*p*)	*r* ^*2*^ (%)	MAF	Environments (methods^a^)
Lu01_826315	T/C	Lu01, 826315	4.15–13.53	4.91–14.53	7.65–18.06	0.40	DL18–19 (5, 3)
Lu01_6408072	A/G	Lu01, 6408072	6.81–10.22	7.67–11.16	9.54–15.12	0.29	AK19–20 (4, 5, 3)
Lu01_17201820	G/A	Lu01, 17201820	4.27–4.79	5.04–5.58	12.77–21.94	0.19	DL18–19 (4, 2, 5)
Lu01_27680796	G/A	Lu01, 27680796	3.17–5.31	3.87–6.12	7.96–10.55	0.48	DL18–19 (2, 5, 3)
**Lu01_27777416**	**T/G**	**Lu01, 27777416**	**3.87–5.01**	**4.62–5.80**	**4.57–6.84**	**0.42**	**AK18**–**19 (5); DL19**–**20 (5, 3)**
Lu02_5607720	A/T	Lu02, 5607720	5.27–6.59	6.08–7.44	7.66–8.10	0.43	DL19–20 (5, 3)
Lu03_8373065	T/C	Lu03, 8373065	3.52–6.76	4.25–7.61	9.75–13.01	0.25	DL17–18 (4, 3)
Lu03_19174892	T/G	Lu03, 19174892	3.72–5.63	4.46–6.45	10.76–13.11	0.25	DL19–20 (2, 5, 3)
Lu03_19423426	A/G	Lu03, 19423426	4.99–10.28	5.79–11.23	10.57–23.06	0.25	AK19–20 (4, 2, 5, 3)
Lu03_24924140	T/C	Lu03, 24924140	5.25–5.76	6.06–6.59	9.48–12.36	0.30	DL17–18 (5, 3)
Lu04_193453	C/G	Lu04, 193453	4.27–5.02	5.03–5.82	4.58–8.67	0.48	DL18–19 (4, 5)
Lu05_7581214	T/C	Lu05, 7581214	3.72–6.75	4.46–7.61	6.97–17.35	0.32	DL17–18 (4, 2, 1, 3)
Lu06_5661970	C/G	Lu06, 5661970	4.93–6.92	5.72–7.78	7.32–15.07	0.27	AK19–20 (4, 5)
Lu06_17131060	C/A	Lu06, 17131060	3.36–3.83	4.08–4.57	10.74–18.18	0.48	AK18–19 (4, 3)
**Lu07_221399**	**A/G**	**Lu07, 221399**	**3.32–7.01**	**4.04–7.87**	**3.68–16.43**	**0.50**	**AK18**–**19 (4, 3); AK19**–**20 (4, 5, 3)**
Lu09_3277312	C/T	Lu09, 3277312	3.61–7.45	4.34–8.33	5.64–9.26	0.47	DL17–18 (4, 2, 3)
Lu09_16962479	C/T	Lu09, 16962479	5.20–7.03	6.00–7.90	9.16–10.36	0.43	DL19–20 (5, 3)
Lu10_11674762	C/G	Lu10, 11674762	5.48–5.73	6.29–6.56	6.35–8.00	0.31	DL19–20 (5, 3)
Lu11_1592089	T/G	Lu11, 1592089	3.23–4.80	3.93–5.59	10.76–11.26	0.22	DL19–20 (2, 5, 3)
Lu12_2201590	T/C	Lu12, 2201590	3.90–5.13	4.64–5.93	6.62–10.05	0.42	DL19–20 (5, 3)
Lu12_4359290	G/A	Lu12, 4359290	3.16–5.37	3.86–6.18	14.35–17.87	0.29	AK18–19 (4, 2, 5)
Lu15_1756429	A/G	Lu15, 1756429	5.03–5.34	5.83–6.15	9.88–10.04	0.27	DL17–18 (4, 2)
Lu15_4682678	G/A	Lu15, 4682678	3.14–8.39	3.84–9.29	4.83–14.62	0.38	AK19–20 (4, 2, 3)
Lu15_6199067	T/C	Lu15, 6199067	4.37–6.02	5.14–6.85	14.78–22.95	0.28	DL18–19 (2, 5, 3)

a Methods: FASTmrEMMA (1), FASTmrMLM (2), ISIS EM-BLASSO (3), mrMLM (4), pLARmEB (5), QTNs, with normal and bold fonts are designated as strong and very strong QTNs, respectively. Underlined QTNs, indicates that it was also identified in DF5 and/or DF50 traits.

**FIGURE 5:**
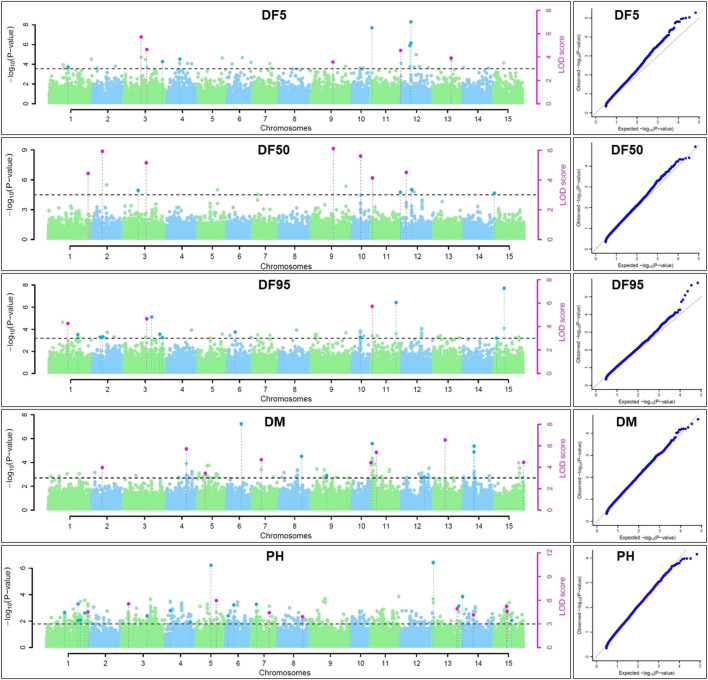
Manhattan plots and quantile-quantile plots for days to 5% (DF5), 50% (DF50), and 95% flowering (DF95), days to maturity (DM), and plant height (PH) using five ML-GWAS methods for environment DL19-20. The dotted lines in Manhattan plots show a threshold at LOD score of ≥3.0. The dots above the threshold depict significant QTNs in the respective chromosome. The pink dots depict significant QTNs identified by ≥2 methods.

**FIGURE 6:**
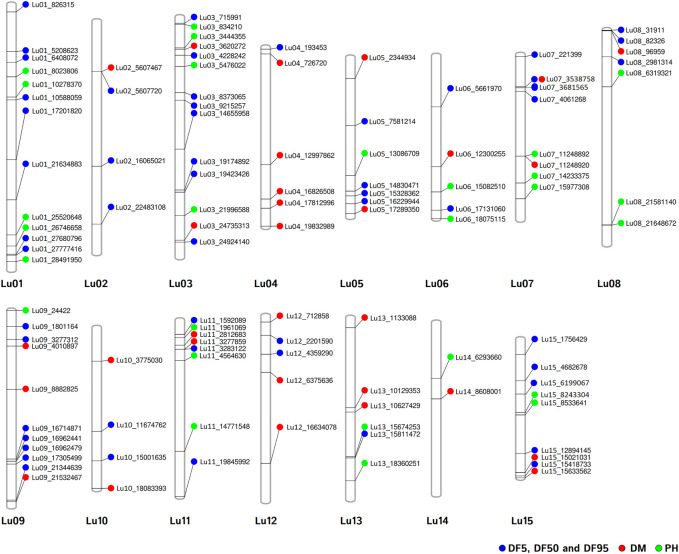
Chromosomal positions of stable QTNs for flowering time (DF5, DF50, and DF95) (blue dots), days to maturity (DM) (red dots), and plant height (PH) (green dots) traits in linseed.

For DM, 30 stable QTNs have been identified, of which 6 and 24 were very strong and strong, respectively (Table 5). For PH, there were 27 stable QTNs comprising 5 very strong and 22 strong QTNs (Table 6). The LOD and −log10(*p*) score of stable QTNs for DM were 3.03–9.17 and 3.73–10.09, respectively, explaining up to 28.78% of variation in days to maturity. For PH, the LOD and -log10(*p*) values of stable QTNs were 3.06–12.15 and 3.76–13.13, respectively. The stable QTNs accounted for phenotypic variation up to 36.6% for PH (Table 6). Manhattan plots showing significant QTNs and respective QQ-plots of ML-GWAS for five traits in five environments are shown (Figure 5; Supplementary Figures S1–S4). Positions of stable QTNs identified for flowering time (DF5, DF50, and DF95), DM, and PH have been depicted on the 15 chromosomes of linseed (Figure 6).

**TABLE 5:** Quantitative trait nucleotides (QTNs) identified for days to maturity (DM).

QTN	Allele	Physical position (bp)	LOD score	−log10(*p*)	*r* ^*2*^ (%)	MAF	Environments (methods^a^)
Lu02_5607467	C/T	Lu02, 5607467	3.20–4.74	3.91–5.53	5.62–7.58	0.30	DL19–20 (4, 5)
Lu03_3620272	C/T	Lu03, 3620272	3.07–4.57	3.77–5.35	7.93–13.57	0.29	DL17–18 (4, 5)
Lu03_24735313	T/A	Lu03, 24735313	3.92–4.09	4.66–4.84	0.81–1.37	0.32	DL17–18 (2, 3)
Lu04_726720	G/A	Lu04, 726720	3.95–6.36	4.70–7.21	7.80–14.35	0.45	AK18–19 (4, 2)
Lu04_12997862	G/A	Lu04, 12997862	3.37–3.68	4.09–4.41	5.94–10.89	0.49	DL18–19 (4, 3)
**Lu04_16826508**	**G/A**	**Lu04, 16826508**	**3.45–6.64**	**4.18–7.50**	**10.99–15.61**	**0.33**	**DL17**–**18 (2, 5); DL19**–**20 (4, 2, 5)**
Lu04_17812996	T/G	Lu04, 17812996	3.95–4.20	4.70–4.97	6.54–9.04	0.27	AK18–19 (4, 5)
Lu04_19832989	A/G	Lu04, 19832989	4.34–4.96	5.11–5.76	4.26–7.85	0.39	AK18–19 (4, 5)
Lu05_2344934	T/C	Lu05, 2344934	3.03–4.40	3.73–5.17	6.43–8.50	0.34	DL19–20 (4, 2, 3)
Lu05_17289350	T/C	Lu05, 17289350	4.23–5.57	4.99–6.38	6.52–12.34	0.40	DL18–19 (4, 5)
**Lu06_12300255**	**C/T**	**Lu06, 12300255**	**4.52–8.04**	**5.29–8.94**	**3.84–4.81**	**0.34**	**DL18**–**19 (3); DL19**–**20 (5)**
Lu07_3538758	T/A	Lu07, 3538758	3.12–6.69	3.82–7.55	7.15–16.42	0.20	DL19–20 (4, 2, 5, 3)
Lu07_11248920	G/A	Lu07, 11248920	3.06–4.78	3.76–5.56	5.29–7.53	0.46	AK19–20 (1, 5, 3)
Lu08_96959	G/T	Lu08, 96959	5.53–6.54	6.35–7.39	5.75–10.83	0.47	DL17–18 (4, 5, 3)
Lu09_4010897	C/T	Lu09, 4010897	3.69–5.28	4.43–6.09	4.42–6.87	0.50	AK18–19 (2, 5, 3)
**Lu09_8882825**	**T/C**	**Lu09, 8882825**	**3.22–6.00**	**3.93–6.84**	**4.94–17.93**	**0.28**	**DL17**–**18 (4, 5, 3); DL19**–**20 (5)**
Lu09_21532467	G/T	Lu09, 21532467	3.93–4.97	4.68–5.77	5.24–6.43	0.40	AK18–19 (4, 5, 3)
Lu10_3775030	T/G	Lu10, 3775030	4.65–4.73	5.43–5.51	11.75–14.21	0.32	AK19–20 (2, 3)
Lu10_18083393	G/T	Lu10, 18083393	3.54–5.36	4.26–6.18	8.89–12.72	0.25	DL19–20 (4, 2, 5, 3)
Lu11_2812683	C/T	Lu11, 2812683	3.61–8.59	4.34–9.49	7.20–21.65	0.23	DL17–18 (4, 5, 3)
**Lu11_3277859**	**C/G**	**Lu11, 3277859**	**3.55–7.23**	**4.28–8.11**	**5.71–7.82**	**0.40**	**DL18**–**19 (5); DL19**–**20 (5, 3)**
**Lu12_712858**	**A/C**	**Lu12, 712858**	**3.49–5.17**	**4.21–5.97**	**12.76–28.78**	**0.25**	**AK19**–**20 (2); DL18**–**19 (4, 5, 3)**
Lu12_6375636	C/T	Lu12, 6375636	3.03–9.17	3.73–10.09	3.07–7.78	0.25	AK18–19 (4, 2, 5)
**Lu12_16634078**	**A/G**	**Lu12, 16634078**	**3.09–3.61**	**3.79–4.34**	**3.56–7.81**	**0.48**	**DL18**–**19 (4); DL19**–**20 (3)**
Lu13_1133088	A/G	Lu13, 1133088	3.15–7.25	3.86–8.12	4.14–7.63	0.27	DL17–18 (2, 5, 3)
Lu13_10129353	A/G	Lu13, 10129353	5.64–7.44	6.46–8.32	10.07–11.77	0.27	DL19–20 (4, 2)
Lu13_10627429	C/T	Lu13, 10627429	3.25–3.56	3.96–4.28	2.36–4.62	0.48	DL17–18 (2, 5)
Lu14_8608001	C/T	Lu14, 8608001	3.16–4.90	3.86–5.69	2.21–4.20	0.28	AK18–19 (4, 2)
Lu15_15021031	G/T	Lu15, 15021031	3.29–3.75	4.01–4.49	2.47–5.53	0.50	AK18–19 (1, 3)
Lu15_15633562	T/A	Lu15, 15633562	4.36–4.54	5.13–5.31	8.79–9.07	0.30	DL19–20 (4, 2)

a Methods: FASTmrEMMA (1), FASTmrMLM (2), ISIS EM-BLASSO (3), mrMLM (4), pLARmEB (5). QTNs, with normal and bold fonts are designated as strong and very strong QTNs, respectively.

**TABLE 6:** Quantitative trait nucleotides (QTNs) identified for plant height (PH).

QTN	Allele	Physical position (bp)	LOD score	−log10(*p*)	*r* ^*2*^ (%)	MAF	Environments (methods^a^)
Lu01_8023806	A/T	Lu01, 8023806	3.39–4.96	4.11–5.75	2.23–4.40	0.47	AK18–19 (5, 3)
**Lu01_10278370**	**T/C**	**Lu01, 10278370**	**4.42–5.90**	**5.19–6.73**	**6.10–11.37**	**0.47**	**AK19**–**20 (1); DL19**–**20 (2)**
**Lu01_25520648**	**A/G**	**Lu01, 25520648**	**3.48–3.97**	**4.21–4.72**	**1.36–6.98**	**0.28**	**DL18**–**19 (5); DL19**–**20 (1)**
**Lu01_26746658**	**C/A**	**Lu01, 26746658**	**3.75–4.37**	**4.48–5.14**	**12.86–36.60**	**0.18**	**AK18**–**19 (4); DL19**–**20 (2)**
**Lu01_28491950**	**A/C**	**Lu01, 28491950**	**3.82–5.94**	**4.56–6.77**	**5.51–7.32**	**0.48**	**DL18**–**19 (5); DL19**–**20 (4, 2)**
Lu03_834210	C/A	Lu03, 834210	5.25–5.33	6.06–6.14	4.59–11.15	0.33	DL17–18 (1, 3)
Lu03_3444355	G/T	Lu03, 3444355	4.17–6.91	4.93–7.77	5.38–7.83	0.34	DL19–20 (4, 3)
Lu03_5476022	T/C	Lu03, 5476022	6.13–6.31	6.97–7.15	9.31–10.24	0.45	AK18–19 (5, 3)
Lu03_21996588	T/C	Lu03, 21996588	11.51–12.15	12.48–13.13	24.36–27.09	0.30	AK19–20 (4, 3)
Lu05_13086709	G/T	Lu05, 13086709	5.77–6.15	6.59–6.99	17.17–19.79	0.19	DL19–20 (4, 2)
Lu06_15082510	G/T	Lu06, 15082510	4.38–5.05	5.15–5.85	5.85–8.45	0.46	DL17–18 (4, 1, 3)
Lu06_18075115	G/A	Lu06, 18075,115	3.57–4.91	4.30–5.71	6.48–14.25	0.37	DL18–19 (4, 3)
Lu07_11248892	T/C	Lu07, 11248892	3.80–4.21	4.54–4.97	3.76–4.98	0.48	AK18–19 (2, 5, 3)
Lu07_14233375	A/G	Lu07, 14233375	3.81–5.00	4.55–5.80	1.83–4.11	0.39	DL19–20 (4, 2)
Lu07_15977308	C/T	Lu07, 15977308	4.43–6.91	5.20–7.77	7.65–15.77	0.48	AK18–19 (5, 3)
Lu08_6319321	G/A	Lu08, 6319321	4.60–4.68	5.38–5.46	4.78–5.60	0.45	AK18–19 (5, 3)
Lu08_21581140	A/G	Lu08, 21581140	3.34–4.48	4.06–5.26	1.33–1.61	0.34	DL19–20 (4, 2)
Lu08_21648672	C/T	Lu08, 21648672	3.55–6.17	4.28–7.01	5.09–12.01	0.47	AK19–20 (5, 3)
Lu09_24422	T/C	Lu09, 24422	4.96–5.80	5.75–6.63	7.02–12.72	0.50	DL17–18 (4, 2, 3)
Lu11_1961069	C/A	Lu11, 1961069	5.08–7.12	5.88–7.99	14.94–20.24	0.34	DL17–18 (2, 3)
Lu11_4564630	A/G	Lu11, 4564630	8.22–9.82	9.12–10.76	18.56–28.47	0.30	DL18–19 (2, 5)
**Lu11_14771548**	**G/T**	**Lu11, 14771548**	**4.65–7.53**	**5.43–8.40**	**10.19–23.40**	**0.34**	**AK18**–**19 (3); AK19**–**20 (5)**
Lu13_15674253	G/T	Lu13, 15674253	3.06–7.59	3.76–8.47	5.74–26.58	0.29	DL17–18 (4, 2, 3)
Lu13_18360251	T/G	Lu13, 18360251	4.36–5.51	5.13–6.32	7.28–8.35	0.38	DL19–20 (4, 2)
Lu14_6293660	A/C	Lu14, 6293660	4.08–4.18	4.84–4.94	3.82–4.66	0.49	DL19–20 (4, 2)
Lu15_8243304	C/T	Lu15, 8243304	4.99–5.95	5.78–6.78	11.29–18.67	0.24	DL19–20 (4, 5, 3)
Lu15_8533641	T/C	Lu15, 8533641	3.97–5.21	4.72–6.01	5.98–9.71	0.27	DL19–20 (5, 3)

a Methods: FASTmrEMMA (1), FASTmrMLM (2), ISIS EM-BLASSO (3), mrMLM (4), pLARmEB (5). QTNs, with normal and bold fonts are designated as strong and very strong QTNs, respectively.

### Identification of Candidate Genes

Genes around the 30 kb region (up and downstream, total 60 kb) of the 109 stable and unique QTNs were considered as the putative candidate genes based on their function, homology with the Arabidopsis ortholog, and pathway analysis. From a total of 201, 273, and 240 genes in the 30 kb region (Supplementary Table S5) of the stable QTNs, 35, 24, and 45 putative candidate genes (Supplementary Table S6) were identified for DF5, DF50, and DF95 traits, respectively. Meanwhile, for DM and PH, 128 and 46 candidate genes were considered as putative candidate genes from the total of 280 and 253 genes, respectively (Supplementary Tables S5, S6). Functional annotation of the putative candidate genes and respective GO prediction to enzyme classes (EC) and pathways (KEGG) have been shown in Supplementary Tables S7–S11. In addition, 70 of the stable QTNs were found within the genic regions (Table 7).

**TABLE 7:** Annotation of candidate genes harboring stable QTNs.

Trait	QTN	Gene	Best Arabidopsis hit	GO biological process/function
DF5	Lu02_16065021	*Lus10006489*	*Abscisic Acid-Insensitive 5- protein*	Abscisic acid-activated signaling
	Lu03_14655958	*Lus10006588*	*Sec-independent protein translocase*	Protein transport
	Lu05_16229944	*Lus10024181*	*Glycosyltransferase-like KOBITO 1*	Cell cycle; cell differentiation
	Lu07_4061268	*Lus10040258*	*Protein DA1-related 2*	Phloem and root development
	Lu08_31911	*Lus10002500*	*Glycerol-3-phosphate 2-O-acyltransferase 6*	Cutin biosynthesis, flower development
	Lu08_2981314	*Lus10002129*	—	—
	Lu09_21344639	*Lus10001785*	*Protein NPGR2*	Calmodulin binding
	Lu10_15001635	*Lus10007230*	—	—
	Lu11_1592089	*Lus10038845*	*Heat shock protein 90–1*	Cellular response to heat
	Lu11_19,845,992	*Lus10001758*	*ABC transporter D family member 2*	Long-chain fatty acid import
	Lu12_2201590	*Lus10023257*	*Small RNA 2′-O-methyltransferase*	Regulation of flower development; specification of floral organ
	Lu15_12894145	*Lus10010329*	*Probable pyruvate Kinase*	Glycolytic process
	Lu15_15418733	*Lus10007858*	*Uncharacterized protein At5g41620*	—
DF50	Lu01_5208623	*Lus10012033*	*RING-H2 finger protein ATL65*	Protein ubiquitination
	Lu01_10588059	*Lus10022584*	*Protein PUTATIVE RECOMBINATION INITIATION DEFECT 1*	Meiotic DNA double-strand break formation
	Lu01_17201820	*Lus10026767*	*Protein FAR1-RELATED SEQUENCE 3*	Regulation of transcription
	Lu01_27777416	*Lus10000368*	*B3 domain- protein REM13*	DNA-binding
	Lu03_4228242	*Lus10019085*	*Transcription factor MYB76*	Glucosinolate biosynthetic process
	Lu03_24924140	*Lus10037684*	*Nuclear pore complex protein*	mRNA export from the nucleus
	Lu05_14830471	*Lus10028400*	*Serine/arginine-rich splicing factor SR45a*	mRNA processing
	Lu07_221399	*Lus10017323*	—	—
	Lu07_3538758	*Lus10023476*	*Adenylate Kinase 1*	Shoot system development
	Lu07_3681565	*Lus10023447*	*Serine carboxypeptidase-like 45*	Probable carboxypeptidase
	Lu08_82326	*Lus10002488*	*LIMR family protein At3g08930*	—
	Lu09_1801164	*Lus10008969*	*Pentatricopeptide repeat- protein*	Embryonic pattern specification
	Lu09_16714871	*Lus10042631*	*Protein LIFEGUARD 4*	—
	Lu09_17305499	*Lus10042564*	—	—
DF95	Lu01_6408072	*Lus10034282*	*Mediator of RNA polymerase II transcription subunit 25*	Positive regulation of flower development
	Lu01_27680796	*Lus10010125*	*Polyubiquitin 10*	Cellular protein modification
	Lu03_8373065	*Lus10000987*	—	—
	Lu04_193453	*Lus10030159*	—	—
	Lu09_3277312	*Lus10007545*	*Phosphoacetylglucosamine mutase*	Carbohydrate metabolic process
	Lu09_16962479	*Lus10042602*	*Increased DNA methylation 1*	Gene silencing
	Lu10_11674762	*Lus10032761*	*Mitogen-activated Protein Kinase Kinase 5*	Activation of MAPK activity
	Lu12_4359290	*Lus10016528*	*3-Ketoacyl-CoA synthase 10*	Fatty acid biosynthetic process
	Lu15_4682678	*Lus10005148*	*Exosome complex component RRP41*	Nuclear mRNA surveillance
DM	Lu03_24735313	*Lus10037719*	*2-component response regulator ARR1*	Regulation of seed growth
	Lu04_726720	*Lus10030282*	*3-phosphoshikimate 1-carboxyvinyltransferase*	Aromatic amino acid biosynthesis
	Lu04_19832989	*Lus10039906*	*3-Ketoacyl-CoA synthase 19*	Fatty acid biosynthetic process
	Lu05_2344934	*Lus10000381*	*AT-hook motif protein 1*	Positioning of chromatin fibers
	Lu06_12300255	*Lus10016021*	*Probable S/T-protein Kinase PBL21*	Defense response
	Lu07_3538758	*Lus10023476*	*Adenylate Kinase 1*	Shoot system development
	Lu08_96959	*Lus10002482*	*Calcium-dependent protein Kinase*	Intracellular signal transduction
	Lu09_4010897	*Lus10040388*	*MLO-like protein 12*	Defense response
	Lu09_21532467	*Lus10004131*	*FRIGIDA-like protein 5*	Flower development
	Lu10_3775030	*Lus10039422*	*LRR receptor-like serine/threonine-protein Kinase GSO2*	Embryo sac development
	Lu11_2812683	*Lus10041958*	*Kinesin-like protein KIN-7D*	Microtubule-based movement
	Lu11_3277859	*Lus10042076*	*Autophagy-related protein 16*	Protein transport
	Lu12_712858	*Lus10006774*	—	—
	Lu12_6375636	*Lus10016836*	*Pumilio homolog 24*	Embryo development ending in seed dormancy
	Lu12_16634078	*Lus10027905*	*Dynamin-related protein 3A*	Cell cycle
	Lu13_1133088	*Lus10010681*	*Protein LURP-one-related 8*	Related to phospholipid scramblase
	Lu13_10627429	*Lus10032833*	*Protein SABRE*	Female organ development
	Lu15_15021031	*Lus10014760*	*Protein DETOXIFICATION 34*	Detoxifying efflux carrier
PH	Lu01_8023806	*Lus10035593*	*Monodehydroascorbate reductase 1*	Oxidizing NADH in the process
	Lu01_26746658	*Lus10000612*	*1-aminocyclopropane-1-carboxylate oxidase homolog 12*	Metal ion binding; oxidoreductase activity
	Lu01_28491950	*Lus10018947*	—	—
	Lu03_834210	*Lus10021899*	*LRR receptor-like S/T- Kinase GSO2*	Embryo sac development; plant organ axis polarity specification
	Lu03_3444355	*Lus10019190*	—	—
	Lu03_5,476,022	*Lus10040542*	—	—
	Lu05_13086709	*Lus10029896*	*Respiratory burst oxidase*	Response to heat; seed germination
	Lu06_15,082,510	*Lus10014399*	*Carotene epsilon-monooxygenase*	Carotenoid biosynthetic process
	Lu08_6319321	*Lus10019044*	*Zinc finger CCCH protein 46*	Possesses RNA-binding and ribonuclease activities *in vitro*
	Lu08_21581140	*Lus10010521*	*Protein phosphatase 2C 70*	Protein dephosphorylation
	Lu08_21648672	*Lus10010533*	*Uclacyanin 1*	Metal ion binding
	Lu09_24422	*Lus10003910*	*C2 & GRAM domain- protein*	Metal ion binding
	Lu11_4564630	*Lus10026287*	*5′-methylthioadenosine/S-adenosylhomocysteine nucleosidase*	Phloem or xylem histogenesis; reproduction
	Lu13_15674253	*Lus10031973*	*PI-PLC X domain-containing protein*	Lipid metabolic process
	Lu13_18360251	*Lus10030871*	*Uncharacterized protein At4g28440*	mRNA binding
	Lu15_8243304	*Lus10005957*	*MAINTENANCE OF MERISTEMS*	Regulation of growth
	Lu15_8533641	*Lus10040926*	*Serpin-Z10*	Endopeptidase activity regulation

For flowering time traits (DF5, DF50, and DF95), the potential candidate genes included *Lus10002500* (*Glycerol-3-phosphate 2-O-acyltransferase 6*), *Lus10023256* (*Protein POLLENLESS 3*), *Lus10024180* (*Protein JINGUBANG*), *Lus10023257* (*Small RNA 2′-O-methyltransferase*), *Lus10022584* (*A PUTATIVE RECOMBINATION INITIATION DEFECT 1*, *PRD1*), *Lus10033883* (*Protein Kinesin Light Chain-Related 2*), *Lus10042078* (*KDEL-tailed cysteine endopeptidase CEP1*), *Lus10040256* (*BEL1-like homeodomain protein 9*), *Lus10013910* (*G-type lectin S-receptor-like serine/threonine-protein kinase RKS1*), *Lus10026767* (*FAR1-related sequence 3*), *Lus10026770* (*F-box protein 2*), *Lus10006489* (*ABA responsive elements-binding factor 2*), *Lus10006587* (*bHLH protein*), *Lus10026766* (*C2H2 zinc-finger protein SERRATE*), *Lus10010121* (*SET domain protein 14*), *Lus10019086* (*MYB domain protein 106*), *Lus10010119* (*Cytochrome P450 family 703*, *subfamily A*, *polypeptide 2*), and *Lus10000989* (*maternal effect embryo arrest 18*) (Table 7, Supplementary Table S6).

For DM, important candidate genes included *Lus10037719* (*Two-component response regulator ARR1*), *Lus10039906* (*3-Ketoacyl-CoA synthase 19*), *Lus10010681* (*Protein LURP-one-related 8*), *Lus10004130* (*Bifunctional* *3-dehydroquinate dehydratase/shikimate dehydrogenase*), *Lus10001717* (*Protein pleiotropic regulatory locus 1*), *Lus10002133* (*CSC1-like protein*), *Lus10001717* (*Protein pleiotropic regulatory locus 1*), *Lus10002492* (*AGAMOUS-like*), *Lus10004131* (*FRIGIDA-like protein*), and *Lus10004132* (*FRIGIDA-like protein*) (Table 7; Supplementary Table S6).

For PH, the potential candidate genes included *Lus10021899* (*LRR receptor-like serine/threonine-protein Kinase GSO2*), *Lus10026287* (*5′-methylthioadenosine/S-adenosylhomocysteine nucleosidase*), *Lus10005957* (*Protein MAINTENANCE OF MERISTEMS*), *Lus10000613* and *Lus10014757* (*lateral organ boundaries domain protein*), *Lus10018946* (*RH39*), *Lus10035590* (*GDSL-like Lipase*), and *Lus10038763* (*Phototropic-responsive NPH3 family protein*) (Table 7; Supplementary Table S6).

### Expression of Candidate Genes

**FIGURE 7:**
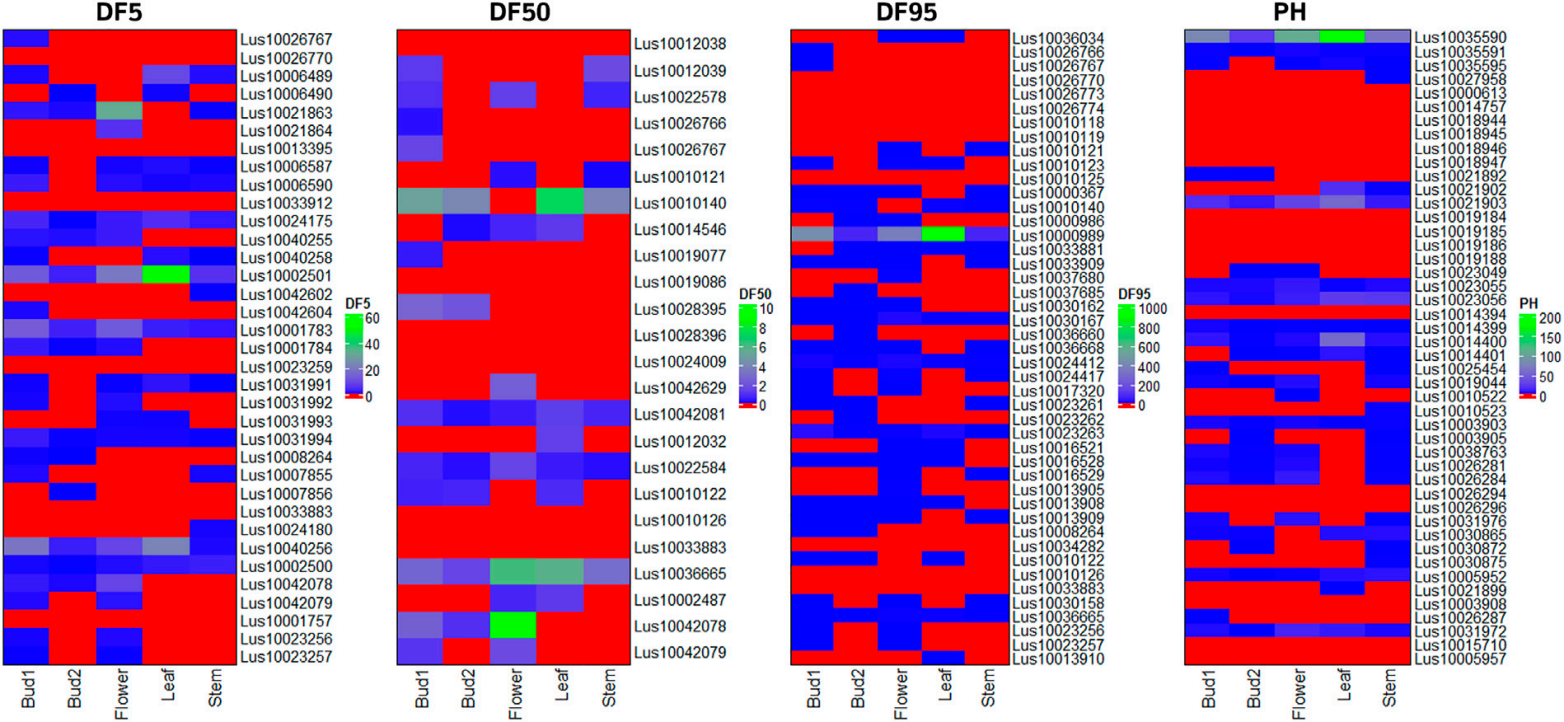
Heat map of expression level of candidate genes for three flowering time traits (DF5, DF50, and DF95) and PH using RNA-seq data of three reproductive tissues (floral buds at two stages of development and flower) and two vegetative tissues (leaf and stem) of early flowering variety *Sharda* (IC0523807).

Putative candidate genes for flowering time (DF5, DF50, and DF95) and PH traits were validated using transcriptome data of floral buds at two developmental stages, flower, leaf, and stem tissues in early flowering and maturing variety of *Sharda* (IC0523807) from the AM panel. Most of the candidate genes for flowering time showed higher expression in at least one of the three floral tissues than in the leaf and/or stem (Figure 7). In case of plant height, 26 of the 46 putative candidate genes showed expression in stem tissue. For DM, expression of candidate genes was studied using *in silico* expression making use of rice RNA-seq data of seeds at 5 and 10 DAP, embryo, and endosperms at 25 DAP. All but 10 of the total candidate genes showed expression in at least one of the four studied tissues (Supplementary Figure S5).

## Discussion

### Trait Variation in Linseed

For the present study, evaluation data of five environments comprising two latitudinally distant geographical locations, New Delhi (28°38′53.7″N 77°09′05.4″E) (years: 2017–18, 2018–19 and 2019–20) and Akola (20°42′03.2″N77°01′53.6″E) (years: 2018–19 and 2019–20), were used. There was significant trait variation recorded within the AM panel for all five traits as evident in the range values (Table 1; Figure 1). Additionally, there was significantly high error variance (environmental factor) for all the studied traits, which impelled us to perform the statistical analysis independently for each individual environment (Table 1). A similar approach has been followed by other researchers for field phenotyping in GWAS studies in linseed (Xie et al., 2018b; Soto-Cerda et al., 2021). The difference in the trait values of both the locations was conspicuous not only for flowering and days to maturity traits but also for plant height (Figure 1). Overall, in Akola, relative to New Delhi, plants were shorter, with earlier flowering and fewer days to maturity. Similar differences in the life cycle of linseed across the latitude in the western Canadian provinces and in southern Chile have also been reported (Soto-Cerda et al., 2021). This could be due to the effect of the photoperiod and temperature on growth and development in linseed (Davidson and Yermanos 1965; Sirohi and Wasnik 2018).

### Importance of Flowering and Maturity Time in Linseed

Different parts of linseed-growing countries have different suitability for early or late flowering and maturity based on prevailing short or long seasons (Sasaki et al., 2018; Raman et al., 2019). Early flowering and maturity are desirable traits in flax-growing regions of Canada and for its expansion in the Canadian Prairies (Miller et al., 2001; Sun et al., 2019). Early maturity is also desirable in south Asian countries, where water is a limiting factor and flowering or maturity coincides with higher temperature so as to avoid adverse effect on seed set and yield (Ford and Zimmerman 1964; Wheeler et al., 2000; Jagadish et al., 2008; Hall et al., 2016). Linseed grown in rice fallows to utilize residual moisture (Kaur et al., 2017; Kaur et al., 2019) also requires early maturing short duration varieties. Cultivation of linseed in rice fallows would not only make efficient use of natural resources but would also bring economic gain for farmers with meager inputs (Singh and Satapathy, 2019). This entails the genetic dissection of flowering time and days to maturity traits to accelerate utilization of genetic and genomic resources for rapid varietal development adapted to different niche areas. Previous study on GWAS in linseed for flowering time trait relied either on days to 5% or 50% flowering (Singh et al., 2019; Soto-Cerda et al., 2021). For further dissection of flowering time, in the present study, GWAS was performed for three flowering time traits, that is, initiation of flowering (days to 5% flowering), days to accomplish 50% flowering, and days to achieve complete flowering (days to 95% flowering). This probably facilitated identification of a higher number of QTNs for flowering time traits than previously reported (You and Cloutier, 2020; Soto-Cerda et al., 2021).

### Comparison of QTNs Identified in Present Study With Previously Identified QTNs/QTLs

In flax/linseed, 14 studies have reported a total of 340 QTLs for 31 quantitative traits (You and Cloutier, 2020; Soto-Cerda et al., 2021). 200 of the QTLs were identified based on genetic maps, the scaffold sequences, or the pre-released chromosome-scale pseudomolecules. The work of You and Cloutier (2020) facilitated the mapping of the SSR and SNP markers from different references onto the recently released chromosome-scale pseudomolecules.

For flowering time, there had been very limited QTLs/associated markers (1 QTL and 2 SNPs) identified until 2020 (Soto-Cerda et al., 2014; Singh et al., 2019; You and Cloutier, 2020). Recently, Soto-Cerda et al. (2021) have reported 27 significant QTLs explaining 2.44%–14.71% of the average phenotypic variation for flowering time in flax in a panel of 200 accessions of the flax core collection using multi-locus GWAS methods. In our study, combining three traits of flowering time (DF5, DF50, and DF95), 53 stable (unique) QTNs on a total of 14 of 15 chromosomes were observed, with the highest number of eight QTNs on chromosomes 1 and 3, followed by seven QTNs on chromosome 9. Comparison of QTNs identified in our study with earlier studies revealed close physical proximity of a number of QTNs with previously reported QTLs. SSR marker Lu943 associated with first flowering time QTL (Soto-Cerda et al., 2014; You and Cloutier, 2020) could be located to 1.02 Mb proximity of QTN Lu01_27777416 identified for DF50 and DF95 in this study. The nearest QTL to Lu943 reported in previous studies was 16 Mb apart on chromosome-1 (Soto-Cerda et al., 2021).

Similarly, a detailed comparison of QTLs identified recently (Soto-Cerda et al., 2021) has revealed that 12 QTNs from the present study were within 2.5 Mb physical distance of 12 of the 27 reported QTLs. A few QTNs, Lu13_15811472, Lu03_715991, Lu05_16229944, and Lu02_22483108, were as close as 0.038 Mb (QTL: Lu13_15849708), 0.07 Mb (QTL: Lu3_637116), 0.6 Mb (QTL: Lu5_16839509), and 0.7 Mb (QTL: Lu2_21773820), respectively, to that of corresponding QTLs. This indicates that these QTNs could be co-located to the respective QTLs. Moreover, additional QTNs have been identified for flowering time traits which could be considered as novel QTNs.

For DM, there have been only three QTLs identified previously, two on chromosome 4, QDTM-Lu4.1 (Coordinates: 13170489–15040682) and QDm.BM.crc-LG4 (Coordinates: 14489225–14489333), and one on chromosome 11, QDTM-Lu11.2 (Coordinates: 14767787) (Kumar et al., 2015; You and Cloutier, 2020). In the present study, for DM, a total of 30 stable QTNs have been identified, of which five were on chromosome 4 (Lu04_16826508, Lu04_726,720, Lu04_12997862, Lu04_17812996, and Lu04_19832989) and two on chromosome 11 (Lu11_2812683 and Lu11_3277859). As could be seen above, two of the identified QTNs appear near to the reported QTL, QDTM-Lu4.1 and QDm.BM.crc-LG4 on chromosome 4, which suggests that they are possibly a part of the respective QTL. Other additional QTNs for days to maturity from our study are novel QTNs for DM.

For PH, there are 30 unique QTLs reported so far on 12 of 15 chromosomes (except on 2, 9, and 10) with the highest number of 9 QTLs on chromosome 1, followed by 4 QTLs on chromosome 3 (Zhang et al., 2018, Soto-Cerda et al., 2014; Wu et al., 2018, Xie et al., 2018b; You and Cloutier, 2020). In our study, a total of 27 stable QTNs have been identified on a total of 11 of 15 chromosomes (except on 2, 4, 10, and 12). On chromosome 1, five QTNs (Table 6) have been identified, of which four QTNs were in close proximity to previously identified QTLs. QTN Lu01_10278370 was localized 0.22, 0.72, and 3.6 Mb close to QTLs scaffold59_572553, scaffold344_309662, and QPLH-Lu1.1, respectively, on the pseudomolecule (You and Cloutier, 2020). Other QTNs, Lu01_8023806, Lu01_28491950, and Lu01_25520648, were close to earlier reported QTLs uq.C1–1, Lu943, and QPLH-Lu1.2 with a distance of 1.4 Mb, 0.3 Mb, and 5.5 Mb, respectively. Similarly, 2 of the 4 QTNs identified in our study were in close proximity to earlier reported QTLs/markers. That includes QTNs Lu03_5476022 (scafold156_641874, 0.43 Mb; Marker4371, 0.54 Mb; scaffold31_1800846, 1.5 Mb) and Lu03_21996588 (uq.C3–1, 3.2 Mb), which suggest that the identified QTNs could be part of earlier reported QTL regions of the chromosomes. So, for PH, the present study has identified QTNs which possibly are part of earlier reported QTLs/markers and some novel QTNs as well; however, we also missed some of the earlier reported QTLs. This could be due to different AM populations and number of SNP markers used.

Overall, in the present study, some of the earlier identified QTLs could be co-located as well as novel QTNs identified for the studied traits. High number of QTNs identified in this study shows the power of ML-GWAS methods for genetic dissection of complex traits over the single locus methods. The previous GWAS studies using single locus methods in linseed had revealed fewer associated loci than GWAS using multi-locus methods (Xie et al., 2018a, b; Singh et al., 2019; Soto-Cerda et al., 2014).

### Identification of Candidate Genes

From the putative candidate genes for flowering time traits, notable genes included *Lus10023257 (small RNA 2′-O-methyltransferase, HEN1, HUA ENHANCER 1)* which carried QTN Lu12_2201590 within the gene sequence and functions like AGAMOUS in organ identity specifications in the flower. It also functions in controlling floral determinacy (Chen et al., 2002). Another important gene near the locus was *Lus10023256 (Protein POLLENLESS 3)* which is essential for male fertility, especially for microspore and pollen grain production in Arabidopsis (Glover et al., 1998). It is specifically involved in the regulation of cell division after male meiosis I and II to facilitate exit from meiosis and transition to G1 (Bulankova et al., 2010).

*Lus10022584 (A PUTATIVE RECOMBINATION INITIATION DEFECT 1, PRD1)* with QTN, Lu01_10588059 in its genic region, and its homologue in Arabidopsis have roles in initiating meiotic recombination by the mechanism of DNA cleavage that forms the double-strand breaks (DSB) to facilitate recombination (De Muyt et al., 2007).

The ortholog of *Lus10026770 (Photo-responsive gene F-box of flowering 2, FOF2)* in Arabidopsis negatively regulates flowering as its overexpression results in late flowering both in long-day and short-day photoperiods whereas mutants show early flowering phenotypes (He et al., 2017).

Interestingly, there were several candidate genes which showed potential function related to pollen or pollen tube development, such as *Lus10033883* (*KINESIN LIGHT CHAIN-RELATED-2*, QTN locus: Lu03_19174892), which showed a role in pollen tube growth, and *Lus10024180* (*Protein JINGUBANG,* QTN locus: Lu05_16229944), a negative regulator of pollen germination functions in stabilizing pollen tube growth that also plays a role in preventing pollination in moist environments by inhibiting jasmonic acid synthesis (Ju et al., 2016). *Lus10042078* (*KDEL-tailed cysteine endopeptidase—CEP1*, QTN locus: Lu11_3283122) plays a role in anther wall tapetum and pollen development (Zhang et al., 2014). Other candidate genes which showed roles related to pollen/pollen tube development were *Ras-related protein RABA4d* (Szumlanski and Nielsen, 2009), *F-box protein* (Gusti et al., 2009), *E3 ubiquitin-protein ligase APD2* (Luo et al., 2012) and among others *LOB domain-containing protein 27, Beclin-1-like protein, Delta-1-pyrroline-5-carboxylate synthase A*, and *G-type lectin S-receptor-like serine/threonine-protein kinase RKS1*.

Among the other notable candidate genes for flowering time, *Lus10042079 (POOR HOMOLOGOUS SYNAPSIS 1)* (QTN locus: Lu11_3283122) plays a role in pairing between homologous chromosomes and accurate chromosome segregation in meiosis (Ronceret et al., 2009). *Lus10002500*, which harbored the QTN Lu08_31911 within the gene, encodes for *glycerol-3-phosphate 2-O-acyltransferase 6*. In Arabdopsis, the *glycerol-3-phosphate acyltransferase 6* genes are expressed in flowering tissues and have a role in the synthesis of the cutin and floral nano-ridges (Li-Beisson et al., 2009).

Interestingly, some other known flowering-related genes such as *FRIGIDA-like protein, AGAMOUS-like*, *Protein ULTRAPETALA 1*, and *Chromatin remodeling protein SHL* were observed near the QTNs associated with DM. Since the flowering time and days to maturity show positive phenotypic correlation (Saroha et al., 2022), it is possible that the QTLs also harbor underlying genes for both the traits.

For DM, important candidate genes include *2-component response regulator ARR1* which showed a role in the regulation of seed growth (Hill et al., 2013) and *Pumilio homolog 24* which is involved in embryogenesis and embryo development ending in seed dormancy (Shanmugam et al., 2017). *Bifunctional 3-dehydroquinate dehydratase/shikimate* functions in embryo development ending in seed dormancy and shikimate pathways (Pagnussat et al., 2005). Soto-Cerda et al. (2021) have reported candidate genes associated with flowering time such as *SUMO activating enzymes, GEM like protein 5*, and *Mannose-6-phosphate isomerase 1* which were involved in embryo development ending in seed dormancy. It is possible that these genes have pleiotropic effect on both the traits. *Protein pleiotropic regulatory locus 1* has a role in cotyledon development and fruit development (Lee et al., 2008). Genes involved in amino acid biosynthesis (*3-phosphoshikimate 1-carboxyvinyltransferase*), fatty acid biosynthesis (*3-Ketoacyl-CoA synthase 19; Probable fructokinase-6*), and carbohydrate metabolism (*UDP-glucuronate 4-epimerase 3*) were also the notable candidate genes for DM.

For PH, candidate genes having a role in phloem or xylem histogenesis (gene: *Lus10021899 - LRR receptor-like serine/threonine-protein Kinase GSO2*), meristem development and regulation of growth (gene: *Lus10026287–5-methylthioadenosine/S-adenosylhomocysteine nucleosidase*), plant-type cell wall biogenesis and xylem and phloem pattern formation (gene: *Lus10003908 - Microtubule-associated protein 70–5*), and cellulose microfibril organization and plant-type secondary cell wall biogenesis (gene: *Lus10031972 - COBRA-like protein 4*) have been identified. Also, a gene, *Lus10015710 (FT-interacting protein 1)*, involved in long-day photoperiodism and positive regulation of flower development has been found near to QTN, Lu14_6293660 associated with PH, indicating a possible pleiotropic effect on both the traits. Interestingly, the two traits showed a positive correction in linseed, as taller plants showed relatively delayed maturity (Saroha et al., 2022). It has been observed that the FT ortholog in tomato (SFT) can also target growth and termination of vegetative apical meristems. The constitutive expression of the *35S:SFT* gene had resulted in shorter internodes, thinner stems, and arrested apices (Lifschitz et al., 2006). The overexpression of *SFT* had also shown attenuation of intercalary meristems of the stems even before and independent of flower formation. Since *SFT/FT* orthologs have pleiotropic effect on flowering and growth, it was suggested that the floral transition and growth attenuation were the two facets of the same cellular responses (Lifschitz et al., 2006). The pleotropic effect of major QTL (comprising a cluster of *FT* genes) for flowering time also had a strong association with growth habit in chickpea (Ortega et al., 2019).

Gene expression profile study showed that most of the candidate genes for flowering time traits and plant height were expressing/showed up-regulation in either the flowering tissues or stem, respectively. Although, the expression profile of candidate genes in the studied tissues gives additional evidence of their role in respective traits, the low or no expression of the candidate genes should not be restrictive at this juncture in terms of their possible role in respective traits as the studied tissues could capture only a limited range of flower/shoot development.

## Conclusion

In the present study, GWAS was performed for three flowering time (DF5, DF50, and DF95) traits, days to maturity, and plant height in linseed using 68,925 SNPs and field evaluation data of five environments by employing five ML-GWAS methods. A total of 335 unique QTNs have been identified, of which 109 were stable QTNs comprising 88 strong and 21 very strong QTNs. For three flowering time traits (DF5, DF50, and DF95), a total of 53 stable QTNs have been identified, whereas for days to maturity and plant height, 30 and 27 stable QTNs have been identified, respectively. Several candidate genes having a role in flower/reproductive system development, seed and fruit development, phloem/xylem histogenesis and pattern formation, regulation of growth, and embryo development ending in seed dormancy have been identified. The study could co-locate the known QTLs as well as identify novel QTNs associated with the studied traits. The present study helps in elucidating QTNs and candidate genes underlying flowering time, days to maturity, and plant height in linseed and improves our understanding of genetic associations of these traits in linseed.

## Data Availability

The datasets presented in this study can be found in online repositories. The names of the repository/repositories and accession number(s) can be found below: https://www.ncbi.nlm.nih.gov/, PRJNA706105.

## Supplementary Material

Supplementary Figure S1 Manhattan plots and quantile-quantile plots for five traits using five ML-GWA methods for environment AK18-19.

Supplementary Figure S2 Manhattan plots and quantile-quantile plots for five traits using five ML-GWA methods for environment AK19-20.

Supplementary Figure S3 Manhattan plots and quantile-quantile plots for five traits using five ML-GWA methods for environment DL17-18.

Supplementary Figure S4 Manhattan plots and quantile-quantile plots for five traits using five ML-GWA methods for environment DL18-19.

Supplementary Figure S5 Heatmap showing *in silico* gene expression of putative candidate genes for DM in flax and their rice (*Oryza sativa*) orthologs based on RNAseq data of four seed developmental stages/tissues of rice obtained from The Rice Genome Annotation Project. DAP, days after pollination.
