# Supplementary material for: Identification of QTNs Associated With Flowering Time, Maturity, and Plant Height Traits in Linum usitatissimum L. Using Genome-Wide Association Study
Source: Front Genet. 2022 Jun 14;13:811924. doi: 10.3389/fgene.2022.811924 (PMC9237403; doi:10.3389/fgene.2022.811924)
Supplement: Supplementary file 1 [file DataSheet2.PDF]

Functional annotation of putative candidate genes for DF50

Query header	Gene name<br>Estimated PPV, Description	Biological process<br>Estimated PPV, GO-id, Description	Molecular function<br>Estimated PPV, GO-id, Description	Cellular component<br>Estimated PPV, GO-id, Description	Inverse EC2GO, Kegg2GO
Lus10024009	0.0 Uncharacterized protein				
Lus10019086	0.69 Myb domain protein 106				
Lus10019077	0.96 two-component response regulator-like APRR1	0.78 GO:0048511 rhythmic process<br>0.63 GO:0000160 phosphorelay signal transduction system<br>0.52 GO:0016310 phosphorylation<br>0.34 GO:2000112 regulation of cellular macromolecule biosynthetic process<br>0.33 GO:0006355 regulation of transcription, DNA-templated	0.53 GO:0016301 kinase activity<br>0.34 GO:0003700 DNA-binding transcription factor activity<br>0.33 GO:0003677 DNA binding	0.60 GO:0005634 nucleus	
Lus10042079	0.0 Uncharacterized protein				
Lus10042081	0.0 Uncharacterized protein				
Lus10042078	0.80 Vignain	0.60 GO:0006508 proteolysis<br>0.48 GO:0044257 cellular protein catabolic process	0.72 GO:0008234 cysteine-type peptidase activity<br>0.46 GO:0004175 endopeptidase activity<br>0.36 GO:0032440 2-alkenal reductase [NAD(P)+] activity	0.50 GO:0005764 lysosome<br>0.49 GO:0005615 extracellular space<br>0.37 GO:0005788 endoplasmic reticulum lumen<br>0.37 GO:0033095 aleurone grain<br>0.32 GO:0016021 integral component of membrane	0.46 EC:3.4.99.- GO:0004175
Lus10010140	0.79 desiccation protectant protein Lea14 homolog	0.84 GO:0009269 response to desiccation		0.34 GO:0016021 integral component of membrane	
Lus10010126	0.93 beclin-1-like protein isoform X1	0.76 GO:0006914 autophagy<br>0.49 GO:0006995 cellular response to nitrogen starvation<br>0.47 GO:0045324 late endosome to vacuole transport<br>0.46 GO:0007033 vacuole organization<br>0.43 GO:0070925 organelle	0.37 GO:0008289 lipid binding	0.50 GO:0034271 phosphatidylinositol 3-kinase complex, class III, type I<br>0.50 GO:0034272 phosphatidylinositol 3-kinase complex, class III, type II<br>0.46 GO:0000407 phagophore assembly site<br>0.32 GO:0016021 integral component of membrane	

			 0.37	 GO:0006869	assembly<br>lipid transport									
Lus10010122	0.0	Uncharacterized protein	0.79	GO:0061458	reproductive system development				0.60	GO:0005634	nucleus			
Lus10010121	0.89	Histone-lysine N-methyltransferase ATX3	0.77<br>0.47<br>0.38	GO:0034968<br>GO:0060255<br>GO:0035556	histone lysine methylation<br>regulation of macromolecule metabolic process<br>intracellular signal transduction	0.78<br>0.54<br>0.37	GO:0018024<br>GO:0046872<br>GO:0003677	histone-lysine N-methyltransferase activity<br>metal ion binding<br>DNA binding	0.59<br>0.32	GO:0005634<br>GO:0016021	nucleus<br>integral component of membrane	0.78	EC:2.1.1.354	GO:0018024
Lus10012039	0.0	Uncharacterized protein												
Lus10012038	0.0	Uncharacterized protein												
Lus10012032	0.71	RAB GTPase homolog A4D				0.68<br>0.66	GO:0003924<br>GO:0005525	GTPase activity<br>GTP binding	0.49<br>0.47	GO:0005768<br>GO:0005794	endosome<br>Golgi apparatus			
Lus10028395	0.58	basic blue protein-like	0.61	GO:0022900	electron transport chain	0.63	GO:0009055	electron transfer activity	0.36	GO:0016021	integral component of membrane			
Lus10028396	0.55	Phytocyanin domain-containing protein	0.61	GO:0022900	electron transport chain	0.63	GO:0009055	electron transfer activity	0.34	GO:0016021	integral component of membrane			
Lus10002487	0.45	RING/U-box superfamily protein isoform 1	0.71	GO:0016567	protein ubiquitination	0.73<br>0.35	GO:0004842<br>GO:0016874	ubiquitin-protein transferase activity<br>ligase activity	0.44	GO:0016021	integral component of membrane	0.35<br>0.73	EC:6.-.-.-<br>KEGG:R03876	GO:0016874<br>GO:0004842
Lus10014546	0.12	PWWP domain-containing protein												
Lus10033883	0.72	Kinesin light chain 3				0.52<br>0.43	GO:0016740<br>GO:0003677	transferase activity<br>DNA binding				0.52	EC:2.-.-.-	GO:0016740
Lus10036665	0.66	LOB domain-containing protein							0.44	GO:0016021	integral component of membrane			
Lus10026767	0.0	Uncharacterized protein												
Lus10026766	0.83	Serrate RNA effector molecule	0.69<br>0.54<br>0.45	GO:0006397<br>GO:0031053<br>GO:2000011	mRNA processing<br>primary miRNA processing<br>regulation of adaxial/abaxial pattern formation				0.60<br>0.45<br>0.43	GO:0005634<br>GO:0070013<br>GO:0005846	nucleus<br>intracellular organelle lumen<br>nuclear cap binding complex			

		0.44 GO:0010267 primary ta-siRNA processing<br>0.44 GO:0031050 ncRNA processing<br>0.44 GO:0048509 regulation of meristem development<br>0.42 GO:0000381 regulation of alternative mRNA splicing, via spliceosome<br>0.42 GO:0048367 shoot system development<br>0.40 GO:0008380 RNA splicing			
Lus10022584	0.0 Uncharacterized protein	0.85 GO:0042138 meiotic DNA double-strand break formation			
Lus10022578	0.10 Beta-galactosidase 8-like				
Lus10042629	0.60 U5 small nuclear ribonucleoprotein 200 kDa helicase-like protein		0.67 GO:0004386 helicase activity<br>0.44 GO:0016787 hydrolase activity		0.44 EC:3.-.-.- GO:0016787
