# Supplementary material for: Identification of QTNs Associated With Flowering Time, Maturity, and Plant Height Traits in Linum usitatissimum L. Using Genome-Wide Association Study
Source: Front Genet. 2022 Jun 14;13:811924. doi: 10.3389/fgene.2022.811924 (PMC9237403; doi:10.3389/fgene.2022.811924)
Supplement: Supplementary file 2 [file DataSheet4.PDF]

Functional annotation of putative candidate genes for DM

Query header	Gene name<br>Estimated PPV, Description	Biological process<br>Estimated PPV, GO-id, Description	Molecular function<br>Estimated PPV, GO-id, Description	Cellular component<br>Estimated PPV, GO-id, Description	Inverse EC2GO, Kegg2GO
Lus10020676	0.0 Uncharacterized protein				
Lus10020681	0.0 Uncharacterized protein				
Lus10020680	0.0 Uncharacterized protein				
Lus10020684	0.47 GATA transcription factor 16	0.67 GO:2000112 regulation of cellular macromolecule biosynthetic process<br>0.58 GO:0006355 regulation of transcription, DNA-templated	0.67 GO:0043565 sequence-specific DNA binding<br>0.64 GO:0008270 zinc ion binding		
Lus10020679	0.0 Uncharacterized protein				
Lus10020677	0.60 Pectinesterase	0.80 GO:0042545 cell wall modification<br>0.79 GO:0045490 pectin catabolic process<br>0.72 GO:0043086 negative regulation of catalytic activity	0.81 GO:0045330 aspartyl esterase activity<br>0.81 GO:0030599 pectinesterase activity<br>0.73 GO:0004857 enzyme inhibitor activity	0.62 GO:0005576 extracellular region<br>0.32 GO:0016021 integral component of membrane	0.81 EC:3.1.1.11 GO:0030599
Lus10020672	0.57 Phospholipase	0.82 GO:0006654 phosphatidic acid biosynthetic process<br>0.80 GO:0048017 inositol lipid-mediated signaling<br>0.72 GO:0016042 lipid catabolic process<br>0.43 GO:0046434 organophosphate catabolic process<br>0.40 GO:0044248 cellular catabolic process	0.84 GO:0004630 phospholipase D activity<br>0.82 GO:0070290 N-acylphosphatidylethanolamine-specific phospholipase D activity<br>0.33 GO:0016491 oxidoreductase activity	0.38 GO:0005886 plasma membrane	0.84 EC:3.1.4.4 GO:0004630
Lus10020671	0.35 PPM-type phosphatase domain-containing protein	0.67 GO:0016311 dephosphorylation	0.68 GO:0016791 phosphatase activity		0.68 EC:3.1.3.- GO:0016791
Lus10020678	0.0 Uncharacterized protein				
Lus10010679	0.60 Serine O-acetyltransferase	0.76 GO:0006535 cysteine biosynthetic process from serine	0.80 GO:0009001 serine O-acetyltransferase activity	0.51 GO:0005737 cytoplasm<br>0.35 GO:0043231 intracellular membrane-bounded organelle<br>0.32 GO:0016021 integral component of membrane	0.80 EC:2.3.1.30 GO:0009001<br>0.80 KEGG:R00586 GO:0009001
Lus10010681	0.81 LURP1-related protein domain containing protein				
Lus10010687	0.0 Uncharacterized protein	0.72 GO:0000398 mRNA splicing, via spliceosome	0.85 GO:0030628 pre-mRNA 3'-splice site binding<br>0.54 GO:0046872 metal ion binding	0.84 GO:0089701 U2AF complex<br>0.74 GO:0005681 spliceosomal complex	
Lus10001711	0.45 Fe2OG dioxygenase domain-containing protein	0.45 GO:0009805 coumarin biosynthetic process<br>0.45 GO:0002238 response to molecule of fungal origin<br>0.37 GO:0051555 flavonol biosynthetic process	0.55 GO:0016491 oxidoreductase activity<br>0.53 GO:0046872 metal ion binding<br>0.39 GO:0031418 L-ascorbic acid binding		0.55 EC:1.-.-.- GO:0016491
Lus10001719	0.21 Superoxide dismutase [Cu-Zn]	0.76 GO:0019430 removal of superoxide radicals	0.78 GO:0004784 superoxide dismutase activity<br>0.54 GO:0046872 metal ion binding		0.78 EC:1.15.1.1 GO:0004784
Lus10001713	0.83 Sterol 14-demethylase	0.50 GO:0016126 sterol biosynthetic process<br>0.44 GO:0032259 methylation<br>0.39 GO:0033075 isoquinoline alkaloid biosynthetic process	0.68 GO:0016705 oxidoreductase activity, acting on paired donors, with incorporation or reduction of molecular oxygen<br>0.68 GO:0004497 monooxygenase activity	0.43 GO:0016021 integral component of membrane	0.68 EC:1.14.-.- GO:0016705

			0.35 GO:0070988 demethylation	0.67 GO:0005506 iron ion binding<br>0.64 GO:0020037 heme binding<br>0.45 GO:0008168 methyltransferase activity<br>0.36 GO:0032451 demethylase activity		
Lus10001714	0.58	Exportin	0.83 GO:0006611 protein export from nucleus<br>0.48 GO:0006405 RNA export from nucleus<br>0.35 GO:0006468 protein phosphorylation	0.83 GO:0005049 nuclear export signal receptor activity<br>0.40 GO:0003723 RNA binding<br>0.39 GO:0031267 small GTPase binding<br>0.35 GO:0004672 protein kinase activity<br>0.34 GO:0005524 ATP binding	0.53 GO:0042565 RNA nuclear export complex<br>0.41 GO:0005634 nucleus<br>0.38 GO:0005737 cytoplasm<br>0.33 GO:0016021 integral component of membrane	
Lus10001716	0.59	probable nucleolar protein 5-2	0.66 GO:0042254 ribosome biogenesis<br>0.36 GO:0002182 cytoplasmic translational elongation	0.81 GO:0030515 snoRNA binding<br>0.33 GO:0003735 structural constituent of ribosome	0.83 GO:0031428 box C/D RNP complex<br>0.79 GO:0032040 small-subunit processome<br>0.70 GO:0005730 nucleolus<br>0.35 GO:0022625 cytosolic large ribosomal subunit	
Lus10001717	0.59	WD repeat-containing protein 48	0.73 GO:0000724 double-strand break repair via homologous recombination<br>0.71 GO:0016579 protein deubiquitination<br>0.45 GO:0010311 lateral root formation<br>0.42 GO:0006468 protein phosphorylation<br>0.42 GO:0006535 cysteine biosynthetic process from serine	0.74 GO:0043130 ubiquitin binding<br>0.52 GO:0016905 myosin heavy chain kinase activity<br>0.42 GO:0004124 cysteine synthase activity		0.52 EC:2.7.11.7 GO:0016905
Lus10001718	0.85	Na+/H+ antiporter family protein 3	0.79 GO:0006885 regulation of pH<br>0.76 GO:0035725 sodium ion transmembrane transport<br>0.63 GO:1902600 proton transmembrane transport<br>0.50 GO:0090333 regulation of stomatal closure<br>0.48 GO:0098659 inorganic cation import across plasma membrane<br>0.48 GO:0030004 cellular monovalent inorganic cation homeostasis<br>0.44 GO:0071805 potassium ion transmembrane transport<br>0.44 GO:0098656 anion transmembrane transport	0.82 GO:0015385 sodium:proton antiporter activity<br>0.49 GO:0015386 potassium:proton antiporter activity	0.44 GO:0016021 integral component of membrane<br>0.38 GO:0005886 plasma membrane	
Lus10009203	0.59	Polyneuridine-aldehyde esterase	0.83 GO:0009696 salicylic acid metabolic process<br>0.83 GO:0009694 jasmonic acid metabolic process<br>0.42 GO:0010224 response to UV-B<br>0.40 GO:0009409 response to cold	0.85 GO:0080032 methyl jasmonate esterase activity<br>0.85 GO:0080031 methyl salicylate esterase activity<br>0.81 GO:0080030 methyl indole-3-acetate esterase activity<br>0.57 GO:0102577 3-oxo-palmitate decarboxylase activity<br>0.57 GO:0050410 3-oxolaurate decarboxylase activity<br>0.57 GO:0102151 3-oxo-myristate decarboxylase activity<br>0.49 GO:0050529 polyneuridine-aldehyde esterase activity<br>0.37 GO:0016746 acyltransferase activity		0.81 EC:3.1.1.1 GO:0080030<br>0.57 KEGG:R03747 GO:0050410

Lus10009205	0.64	Polyneuridine-aldehyde esterase	0.75<br>0.75<br>0.49	GO:0009696<br>GO:0009694<br>GO:0009820	salicylic acid metabolic process<br>jasmonic acid metabolic process<br>alkaloid metabolic process	0.79<br>0.79<br>0.74<br>0.62<br>0.62<br>0.62<br>0.50	GO:0080032<br>GO:0080031<br>GO:0080030<br>GO:0102577<br>GO:0050410<br>GO:0102151<br>GO:0050529	methyl jasmonate esterase activity<br>methyl salicylate esterase activity<br>methyl indole-3-acetate esterase activity<br>3-oxo-palmitate decarboxylase activity<br>3-oxolaurate decarboxylase activity<br>3-oxo-myristate decarboxylase activity<br>polyneuridine-aldehyde esterase activity	0.34	GO:0016021	integral component of membrane	0.74<br>0.62	EC:3.1.1.1<br>KEGG:R03747	GO:0080030<br>GO:0050410
Lus10014764	0.76	ATP-dependent helicase BRM isoform X1	0.80<br>0.67<br>0.67<br>0.59	GO:0040029<br>GO:0048856<br>GO:2000112<br>GO:0045944	regulation of gene expression, epigenetic<br>anatomical structure development<br>regulation of cellular macromolecule biosynthetic process<br>positive regulation of transcription by RNA polymerase II	0.68<br>0.67<br>0.56<br>0.48	GO:0008094<br>GO:0004386<br>GO:0005524<br>GO:0003677	ATP-dependent activity, acting on DNA<br>helicase activity<br>ATP binding<br>DNA binding	0.60	GO:0005634	nucleus			
Lus10014762	0.54	AT-rich interactive domain-containing protein 1				0.53	GO:0003677	DNA binding	0.56	GO:0005634	nucleus			
Lus10014760	0.0	Uncharacterized protein												
Lus10014759	0.0	Uncharacterized protein												
Lus10014763	0.51	Ubiquitin-conjugating enzyme E2 4	0.54<br>0.50	GO:0000209<br>GO:0006511	protein polyubiquitination<br>ubiquitin-dependent protein catabolic process	0.58<br>0.56<br>0.37<br>0.36	GO:0061631<br>GO:0005524<br>GO:0016746<br>GO:0004839	ubiquitin conjugating enzyme activity<br>ATP binding<br>acyltransferase activity<br>ubiquitin activating enzyme activity	0.44<br>0.32	GO:0005634<br>GO:0016021	nucleus<br>integral component of membrane	0.58	EC:2.3.2.23	GO:0061631
Lus10014765	0.58	bifunctional L-3-cyanoalanine synthase/cysteine synthase 1, mitochondrial	0.86<br>0.76<br>0.39<br>0.35	GO:0019499<br>GO:0006535<br>GO:0009836<br>GO:0009611	cyanide metabolic process<br>cysteine biosynthetic process from serine<br>fruit ripening, climacteric<br>response to wounding	0.87<br>0.79<br>0.34	GO:0050017<br>GO:0004124<br>GO:0030170	L-3-cyanoalanine synthase activity<br>cysteine synthase activity<br>pyridoxal phosphate binding	0.62	GO:0005739	mitochondrion	0.87	EC:4.4.1.9	GO:0050017
Lus10011626	0.58	F-box/LRR-repeat protein 4-like	0.72<br>0.51<br>0.50	GO:0031146<br>GO:0006955<br>GO:0098542	SCF-dependent proteasomal ubiquitin-dependent protein catabolic process<br>immune response<br>defense response to other organism				0.72<br>0.40	GO:0019005<br>GO:0005737	SCF ubiquitin ligase complex<br>cytoplasm			
Lus10006663	0.46	Clp R domain-containing protein	0.54	GO:0006508	proteolysis	0.64<br>0.56<br>0.46	GO:0043424<br>GO:0008233<br>GO:0005524	protein histidine kinase binding<br>peptidase activity<br>ATP binding	0.59	GO:0009532	plastid stroma	0.56	EC:3.4.-.-	GO:0008233
Lus10030275	0.94	transducin beta-like protein 2				0.35	GO:0016757	glycosyltransferase activity	0.44	GO:0016021	integral component of membrane	0.35	EC:2.4.-.-	GO:0016757
Lus10030287	0.56	Peptide transporter, putative	0.55<br>0.42<br>0.40<br>0.40<br>0.40	GO:0055085<br>GO:0015698<br>GO:0010167<br>GO:0010150<br>GO:0055075	transmembrane transport<br>inorganic anion transport<br>response to nitrate<br>leaf senescence<br>potassium ion homeostasis	0.57	GO:0022857	transmembrane transporter activity	0.44<br>0.34	GO:0016021<br>GO:0005886	integral component of membrane<br>plasma membrane			

			0.37<br>0.37<br>0.34	GO:0006813<br>GO:0071705<br>GO:0071702	potassium ion transport<br>nitrogen compound transport<br>organic substance transport									
Lus10030288	0.56	Ureohydrolase	0.55	GO:0033389	putrescine biosynthetic process from arginine, using agmatinase	0.76<br>0.54	GO:0016813<br>GO:0046872	hydrolase activity, acting on carbon-nitrogen (but not peptide) bonds, in linear amidines<br>metal ion binding				0.76	EC:3.5.3.-	GO:0016813
Lus10030279	0.43	Protein kinase domain-containing protein	0.64<br>0.37<br>0.36<br>0.36	GO:0006468<br>GO:0006955<br>GO:0098542<br>GO:0007178	protein phosphorylation<br>immune response<br>defense response to other organism<br>transmembrane receptor protein serine/threonine kinase signaling pathway	0.64<br>0.56<br>0.35<br>0.34	GO:0004672<br>GO:0005524<br>GO:0004888<br>GO:0005515	protein kinase activity<br>ATP binding<br>transmembrane signaling receptor activity<br>protein binding	0.44<br>0.38	GO:0016021<br>GO:0005886	integral component of membrane<br>plasma membrane			
Lus10030283	0.61	Transketolase	0.44	GO:0006098	pentose-phosphate shunt	0.80<br>0.54	GO:0004802<br>GO:0046872	transketolase activity<br>metal ion binding	0.42<br>0.36	GO:0005829<br>GO:0009507	cytosol<br>chloroplast	0.80	EC:2.2.1.1	GO:0004802
Lus10030286	0.51	Solute carrier family 15 (Peptide/histidine transporter), member 3/4	0.55<br>0.54<br>0.46<br>0.40<br>0.40<br>0.39<br>0.37<br>0.35<br>0.35	GO:0055085<br>GO:0006817<br>GO:0050896<br>GO:0010150<br>GO:0055075<br>GO:0015706<br>GO:0006813<br>GO:0006857<br>GO:0042128	transmembrane transport<br>phosphate ion transport<br>response to stimulus<br>leaf senescence<br>potassium ion homeostasis<br>nitrate transport<br>potassium ion transport<br>oligopeptide transport<br>nitrate assimilation	0.57	GO:0022857	transmembrane transporter activity	0.44<br>0.34	GO:0016021<br>GO:0005886	integral component of membrane<br>plasma membrane			
Lus10030285	0.91	Probable tRNA N6-adenosine threonylcarbamoyltransferase, mitochondrial	0.75<br>0.53<br>0.34<br>0.33	GO:0002949<br>GO:0009793<br>GO:0006281<br>GO:0006508	tRNA threonylcarbamoyladenosine modification<br>embryo development ending in seed dormancy<br>DNA repair<br>proteolysis	0.79<br>0.54<br>0.34<br>0.34<br>0.33<br>0.33<br>0.33	GO:0061711<br>GO:0046872<br>GO:0016787<br>GO:0008094<br>GO:0140096<br>GO:0003677<br>GO:0005524	N(6)-L-threonylcarbamoyladenine synthase activity<br>metal ion binding<br>hydrolase activity<br>ATP-dependent activity, acting on DNA<br>catalytic activity, acting on a protein<br>DNA binding<br>ATP binding	0.61<br>0.44<br>0.32	GO:0005739<br>GO:0019866<br>GO:0016021	mitochondrion<br>organelle inner membrane<br>integral component of membrane	0.79	EC:2.3.1.234	GO:0061711
Lus10042074	0.63	Protein DETOXIFICATION	0.73<br>0.55<br>0.39<br>0.39<br>0.37<br>0.37<br>0.37	GO:0042908<br>GO:0055085<br>GO:0009697<br>GO:0009751<br>GO:0042742<br>GO:0045087<br>GO:0031348	xenobiotic transport<br>transmembrane transport<br>salicylic acid biosynthetic process<br>response to salicylic acid<br>defense response to bacterium<br>innate immune response<br>negative regulation of defense response	0.74<br>0.71	GO:0042910<br>GO:0015297	xenobiotic transmembrane transporter activity<br>antiporter activity	0.44<br>0.40<br>0.34	GO:0016021<br>GO:0009941<br>GO:0042170	integral component of membrane<br>chloroplast envelope<br>plastid membrane			
Lus10041959	0.59	Purple acid phosphatase	0.67<br>0.38<br>0.35	GO:0016311<br>GO:0006950<br>GO:0031669	dephosphorylation<br>response to stress<br>cellular response to nutrient levels	0.79<br>0.54<br>0.35	GO:0003993<br>GO:0046872<br>GO:0016746	acid phosphatase activity<br>metal ion binding<br>acyltransferase activity	0.36<br>0.36<br>0.36<br>0.34<br>0.33	GO:0009506<br>GO:0009505<br>GO:0005576<br>GO:0016021<br>GO:0005737	plasmodesma<br>plant-type cell wall<br>extracellular region<br>integral component of membrane<br>cytoplasm	0.79	EC:3.1.3.2	GO:0003993

Lus10042080	0.75	MA3 DOMAIN-CONTAINING TRANSLATION REGULATORY FACTOR 1-like	0.74 GO:2000113 negative regulation of cellular macromolecule biosynthetic process<br>0.71 GO:0045892 negative regulation of transcription, DNA-templated<br>0.71 GO:0006417 regulation of translation<br>0.37 GO:0006413 translational initiation	0.37 GO:0003743 translation initiation factor activity	0.51 GO:0005737 cytoplasm	
Lus10041963	0.90	SNF2 domain-containing protein / helicase domain-containing protein	0.85 GO:0080188 gene silencing by RNA-directed DNA methylation<br>0.36 GO:0032508 DNA duplex unwinding<br>0.33 GO:0016310 phosphorylation	0.56 GO:0005524 ATP binding<br>0.42 GO:0004386 helicase activity<br>0.36 GO:0008094 ATP-dependent activity, acting on DNA<br>0.34 GO:0016787 hydrolase activity<br>0.34 GO:0016301 kinase activity	0.32 GO:0016021 integral component of membrane	0.34 EC:3.-.-.- GO:0016787
Lus10041965	0.45	Nudix hydrolase domain-containing protein	0.55 GO:0015938 coenzyme A catabolic process<br>0.36 GO:0006104 succinyl-CoA metabolic process<br>0.35 GO:0015937 coenzyme A biosynthetic process	0.81 GO:0010945 CoA pyrophosphatase activity<br>0.51 GO:0003986 acetyl-CoA hydrolase activity<br>0.36 GO:0008893 guanosine-3',5'-bis(diphosphate) 3'-diphosphatase activity<br>0.34 GO:0046872 metal ion binding	0.34 GO:0009507 chloroplast<br>0.34 GO:0005739 mitochondrion<br>0.32 GO:0016021 integral component of membrane	0.51 EC:3.1.2.1 GO:0003986<br>0.51 KEGG:R00227 GO:0003986
Lus10042076	0.75	autophagy-related protein 16	0.81 GO:0000045 autophagosome assembly<br>0.40 GO:0006468 protein phosphorylation	0.49 GO:0016905 myosin heavy chain kinase activity	0.84 GO:0000421 autophagosome membrane	0.49 EC:2.7.11.7 GO:0016905
Lus10041951	0.0	Uncharacterized protein	0.40 GO:0051762 sesquiterpene biosynthetic process<br>0.35 GO:0019438 aromatic compound biosynthetic process<br>0.35 GO:1901362 organic cyclic compound biosynthetic process<br>0.35 GO:0016114 terpenoid biosynthetic process	0.69 GO:0016705 oxidoreductase activity, acting on paired donors, with incorporation or reduction of molecular oxygen<br>0.68 GO:0004497 monooxygenase activity<br>0.67 GO:0005506 iron ion binding<br>0.64 GO:0020037 heme binding	0.42 GO:0016021 integral component of membrane	0.69 EC:1.14.-.- GO:0016705
Lus10041964	0.65	26S protease regulatory subunit 10B A	0.79 GO:1901800 positive regulation of proteasomal protein catabolic process<br>0.70 GO:0030163 protein catabolic process<br>0.54 GO:0045899 positive regulation of RNA polymerase II transcription preinitiation complex assembly<br>0.50 GO:0034976 response to endoplasmic reticulum stress<br>0.49 GO:0010243 response to organonitrogen compound<br>0.49 GO:0006508 proteolysis<br>0.47 GO:0043632 modification-dependent macromolecule catabolic process<br>0.45 GO:2000112 regulation of cellular macromolecule biosynthetic process<br>0.40 GO:0044267 cellular protein metabolic process	0.80 GO:0036402 proteasome-activating activity<br>0.65 GO:0016887 ATP hydrolysis activity<br>0.56 GO:0005524 ATP binding<br>0.47 GO:0008233 peptidase activity	0.73 GO:0000502 proteasome complex<br>0.56 GO:0005634 nucleus<br>0.51 GO:0005737 cytoplasm	0.80 EC:5.6.1.5 GO:0036402
Lus10041958	0.89	kinesin-like protein KIN-7D, mitochondrial	0.74 GO:0007018 microtubule-based movement	0.76 GO:0003777 microtubule motor activity<br>0.75 GO:0008017 microtubule binding<br>0.56 GO:0005524 ATP binding<br>0.41 GO:0016887 ATP hydrolysis activity	0.45 GO:0005871 kinesin complex<br>0.43 GO:0005874 microtubule<br>0.32 GO:0016021 integral component of membrane	
Lus10042079	0.0	Uncharacterized protein				

Lus10042081	0.0	Uncharacterized protein				
Lus10041953	0.78	leucine-rich repeat receptor-like serine/threonine/tyrosine-protein kinase SOBIR1	0.63 GO:0006468 protein phosphorylation<br>0.38 GO:0006955 immune response<br>0.38 GO:0098542 defense response to other organism<br>0.35 GO:0018212 peptidyl-tyrosine modification	0.64 GO:0004672 protein kinase activity<br>0.55 GO:0005524 ATP binding<br>0.34 GO:0004888 transmembrane signaling receptor activity	0.44 GO:0016021 integral component of membrane<br>0.40 GO:0005886 plasma membrane	
Lus10041960	0.97	Chromatin remodeling protein SHL	0.71 GO:0006325 chromatin organization<br>0.39 GO:0035067 negative regulation of histone acetylation<br>0.39 GO:0010228 vegetative to reproductive phase transition of meristem<br>0.38 GO:2000028 regulation of photoperiodism, flowering<br>0.36 GO:0070544 histone H3-K36 demethylation<br>0.36 GO:0032259 methylation<br>0.36 GO:2000112 regulation of cellular macromolecule biosynthetic process<br>0.34 GO:0006355 regulation of transcription, DNA-templated	0.77 GO:0003682 chromatin binding<br>0.54 GO:0046872 metal ion binding<br>0.38 GO:0140034 methylation-dependent protein binding<br>0.37 GO:0042393 histone binding<br>0.37 GO:0000976 transcription cis-regulatory region binding<br>0.36 GO:0140680 histone H3-di/monomethyl-lysine-36 demethylase activity<br>0.36 GO:0008168 methyltransferase activity	0.35 GO:0005634 nucleus<br>0.32 GO:0016021 integral component of membrane	0.36 EC:1.14.11.27 GO:0140680
Lus10042078	0.80	Vignain	0.60 GO:0006508 proteolysis<br>0.48 GO:0044257 cellular protein catabolic process	0.72 GO:0008234 cysteine-type peptidase activity<br>0.46 GO:0004175 endopeptidase activity<br>0.36 GO:0032440 2-alkenal reductase [NAD(P)+] activity	0.50 GO:0005764 lysosome<br>0.49 GO:0005615 extracellular space<br>0.37 GO:0005788 endoplasmic reticulum lumen<br>0.37 GO:0033095 aleurone grain<br>0.32 GO:0016021 integral component of membrane	0.46 EC:3.4.99.- GO:0004175
Lus10041955	0.63	Protein DETOXIFICATION	0.73 GO:0042908 xenobiotic transport<br>0.55 GO:0055085 transmembrane transport	0.74 GO:0042910 xenobiotic transmembrane transporter activity<br>0.71 GO:0015297 antiporter activity	0.44 GO:0016021 integral component of membrane<br>0.35 GO:0009941 chloroplast envelope	
Lus10042082	0.92	BTB/POZ and TAZ domain-containing protein 1-like	0.85 GO:0009751 response to salicylic acid<br>0.84 GO:0042542 response to hydrogen peroxide<br>0.74 GO:0009725 response to hormone<br>0.50 GO:0016567 protein ubiquitination	0.77 GO:0005516 calmodulin binding<br>0.54 GO:0046872 metal ion binding	0.43 GO:0005634 nucleus	
Lus10041962	0.58	Nuclear fusion defective	0.54 GO:0006817 phosphate ion transport<br>0.45 GO:0050896 response to stimulus<br>0.35 GO:0055085 transmembrane transport	0.35 GO:0022857 transmembrane transporter activity	0.44 GO:0016021 integral component of membrane	
Lus10041961	0.0	Uncharacterized protein				
Lus10042075	0.56	alpha/beta hydrolase domain-containing protein 17C-like isoform X2	0.47 GO:0006508 proteolysis<br>0.39 GO:0009820 alkaloid metabolic process	0.53 GO:0016787 hydrolase activity<br>0.46 GO:0140096 catalytic activity, acting on a protein		0.53 EC:3.-.-.- GO:0016787
Lus10016026	0.56	Homeodomain protein HB1	0.67 GO:2000112 regulation of cellular macromolecule biosynthetic process<br>0.57 GO:0006355 regulation of transcription, DNA-templated	0.62 GO:0003700 DNA-binding transcription factor activity<br>0.57 GO:0003677 DNA binding	0.59 GO:0005634 nucleus	
Lus10016018	0.76	Plant regulator RWP-RK family protein	0.67 GO:2000112 regulation of cellular macromolecule biosynthetic process<br>0.58 GO:0006355 regulation of transcription,	0.62 GO:0003700 DNA-binding transcription factor activity<br>0.37 GO:0000976 transcription cis-regulatory region binding	0.35 GO:0005634 nucleus	

					DNA-templated									
Lus10027899	0.78	Glycoprotein 3-alpha-L-fucosyltransferase A	0.80<br>0.73	GO:0036065<br>GO:0006486	fucosylation<br>protein glycosylation	0.81<br>0.40	GO:0008417<br>GO:0140103	fucosyltransferase activity<br>catalytic activity, acting on a glycoprotein	0.79<br>0.44	GO:0032580<br>GO:0016021	Golgi cisterna membrane<br>integral component of membrane			
Lus10027909	0.48	CXE carboxylesterase	0.34	GO:0006260	DNA replication	0.53	GO:0016787	hydrolase activity	0.36<br>0.34	GO:0000808<br>GO:0005634	origin recognition complex<br>nucleus	0.53	EC:3.-.-.-	GO:0016787
Lus10027904	0.73	prostaglandin reductase-3	0.41	GO:0006979	response to oxidative stress	0.61<br>0.55	GO:0008270<br>GO:0016491	zinc ion binding<br>oxidoreductase activity				0.55	EC:1.-.-.-	GO:0016491
Lus10027903	0.62	Leucine-rich repeat receptor-like serine/threonine-protein kinase	0.56<br>0.47<br>0.46<br>0.38	GO:0016310<br>GO:0006955<br>GO:0098542<br>GO:0018212	phosphorylation<br>immune response<br>defense response to other organism<br>peptidyl-tyrosine modification	0.58<br>0.37<br>0.36<br>0.36<br>0.35	GO:0016301<br>GO:0004888<br>GO:0016853<br>GO:0016773<br>GO:0140096	kinase activity<br>transmembrane signaling receptor activity<br>isomerase activity<br>phosphotransferase activity, alcohol group as acceptor<br>catalytic activity, acting on a protein	0.33	GO:0016021	integral component of membrane	0.36	EC:5.-.-.-	GO:0016853
Lus10004131	0.0	Uncharacterized protein												
Lus10004130	0.97	bifunctional 3-dehydroquinate dehydratase/shikimate dehydrogenase, chloroplastic-like	0.67<br>0.60<br>0.50<br>0.48	GO:0009073<br>GO:0008652<br>GO:0019632<br>GO:0009423	aromatic amino acid family biosynthetic process<br>cellular amino acid biosynthetic process<br>shikimate metabolic process<br>chorismate biosynthetic process	0.79<br>0.79	GO:0003855<br>GO:0004764	3-dehydroquinate dehydratase activity<br>shikimate 3-dehydrogenase (NADP+) activity				0.79<br>0.79	EC:4.2.1.10<br>KEGG:R03084	GO:0003855<br>GO:0003855
Lus10004139	0.47	Superoxide dismutase [Cu-Zn]	0.76	GO:0019430	removal of superoxide radicals	0.78<br>0.54	GO:0004784<br>GO:0046872	superoxide dismutase activity<br>metal ion binding	0.35	GO:0005737	cytoplasm	0.78	EC:1.15.1.1	GO:0004784
Lus10004132	0.0	Uncharacterized protein												
Lus10004140	0.0	Uncharacterized protein												
Lus10000384	0.80	Cornichon domain-containing protein	0.68<br>0.39	GO:0016192<br>GO:0009987	vesicle-mediated transport<br>cellular process				0.44	GO:0016021	integral component of membrane			
Lus10000382	0.41	50S ribosomal protein L17, chloroplastic	0.57	GO:0006412	translation	0.58	GO:0003735	structural constituent of ribosome	0.56<br>0.45<br>0.45<br>0.33	GO:0005840<br>GO:0005829<br>GO:1990904<br>GO:0016021	ribosome<br>cytosol<br>ribonucleoprotein complex<br>integral component of membrane			
Lus10000387	0.0	Uncharacterized protein												
Lus10000381	0.65	AT-hook motif nuclear-localized protein				0.84<br>0.36	GO:0003680<br>GO:0043565	minor groove of adenine-thymine-rich DNA binding<br>sequence-specific DNA binding	0.60<br>0.38<br>0.36<br>0.34	GO:0005634<br>GO:0098687<br>GO:0070013<br>GO:0005739	nucleus<br>chromosomal region<br>intracellular organelle lumen<br>mitochondrion			
Lus10023470	0.57	Protein-serine/threonine phosphatase	0.71<br>0.39	GO:0006470<br>GO:1900425	protein dephosphorylation<br>negative regulation of defense response to bacterium	0.75<br>0.75<br>0.54<br>0.34	GO:0106307<br>GO:0106306<br>GO:0046872<br>GO:0005515	protein serine/threonine phosphatase activity<br>protein serine/threonine phosphatase activity<br>metal ion binding<br>protein binding	0.40<br>0.34	GO:0016021<br>GO:0005886	integral component of membrane<br>plasma membrane			

Lus10023469	0.56	Rubis-subs-bind domain-containing protein	0.71	GO:0018026	peptidyl-lysine monomethylation	0.65	GO:0016279	protein-lysine N-methyltransferase activity						
Lus10023475	0.78	Elongator complex protein 6	0.76<br>0.53<br>0.52<br>0.52<br>0.50<br>0.50<br>0.48<br>0.45	GO:0002098<br>GO:0031538<br>GO:2000024<br>GO:0009926<br>GO:0010015<br>GO:0010016<br>GO:0008284<br>GO:0006979	tRNA wobble uridine modification<br>negative regulation of anthocyanin metabolic process<br>regulation of leaf development<br>auxin polar transport<br>root morphogenesis<br>shoot system morphogenesis<br>positive regulation of cell population proliferation<br>response to oxidative stress	0.34<br>0.34	GO:0005515<br>GO:0016746	protein binding<br>acyltransferase activity	0.82<br>0.34<br>0.33	GO:0033588<br>GO:0009507<br>GO:0005634	elongator holoenzyme complex<br>chloroplast<br>nucleus	0.34	EC:2.3.-.-	GO:0016746
Lus10023479	0.0	Uncharacterized protein												
Lus10023471	0.79	CDP-diacylglycerol--glycerol-3-phosphate 3-phosphatidyltransferase 2	0.67<br>0.49<br>0.48<br>0.36	GO:0008654<br>GO:0045017<br>GO:0006650<br>GO:0010027	phospholipid biosynthetic process<br>glycerolipid biosynthetic process<br>glycerophospholipid metabolic process<br>thylakoid membrane organization	0.79<br>0.37<br>0.35	GO:0008444<br>GO:0043337<br>GO:0030145	CDP-diacylglycerol-glycerol-3-phosphate 3-phosphatidyltransferase activity<br>CDP-diacylglycerol-phosphatidylglycerol phosphatidyltransferase activity<br>manganese ion binding	0.44<br>0.35<br>0.35<br>0.34<br>0.34	GO:0016021<br>GO:0031969<br>GO:0005739<br>GO:0005794<br>GO:0019866	integral component of membrane<br>chloroplast membrane<br>mitochondrion<br>Golgi apparatus<br>organelle inner membrane	0.79<br>0.79	EC:2.7.8.5<br>KEGG:R01801	GO:0008444<br>GO:0008444
Lus10023477	0.65	ERAD-associated E3 ubiquitin-protein ligase HRD1B-like	0.44<br>0.44	GO:0006511<br>GO:0016567	ubiquitin-dependent protein catabolic process<br>protein ubiquitination	0.45<br>0.37<br>0.37	GO:0061630<br>GO:0016746<br>GO:0016874	ubiquitin protein ligase activity<br>acyltransferase activity<br>ligase activity	0.44	GO:0016021	integral component of membrane	0.37	EC:2.3.-.-	GO:0016746
Lus10006771	0.71	Derlin	0.62<br>0.49<br>0.49<br>0.47<br>0.42	GO:0006950<br>GO:0010243<br>GO:0043161<br>GO:0071310<br>GO:0007165	response to stress<br>response to organonitrogen compound<br>proteasome-mediated ubiquitin-dependent protein catabolic process<br>cellular response to organic substance<br>signal transduction	0.55<br>0.54	GO:1990381<br>GO:0051787	ubiquitin-specific protease binding<br>misfolded protein binding	0.70<br>0.53<br>0.49<br>0.48<br>0.44	GO:0005789<br>GO:0000153<br>GO:0140534<br>GO:0031301<br>GO:0098796	endoplasmic reticulum membrane<br>cytoplasmic ubiquitin ligase complex<br>endoplasmic reticulum protein-containing complex<br>integral component of organelle membrane<br>membrane protein complex			
Lus10006766	0.55	Polyadenylation and cleavage factor homolog 4	0.85<br>0.82<br>0.81<br>0.55	GO:0006369<br>GO:0006379<br>GO:0006378<br>GO:0034645	termination of RNA polymerase II transcription<br>mRNA cleavage<br>mRNA polyadenylation<br>cellular macromolecule biosynthetic process	0.84<br>0.63	GO:0000993<br>GO:0003729	RNA polymerase II complex binding<br>mRNA binding	0.57<br>0.41	GO:0005849<br>GO:0005737	mRNA cleavage factor complex<br>cytoplasm			
Lus10038081	0.76	Polyadenylate-binding protein-interacting protein 7				0.51<br>0.40	GO:0043130<br>GO:0016787	ubiquitin binding<br>hydrolase activity	0.41	GO:0016021	integral component of membrane	0.40	EC:3.-.-.-	GO:0016787
Lus10038082	0.66	Ethylene-responsive transcription factor ERF018	0.67<br>0.58	GO:2000112<br>GO:0006355	regulation of cellular macromolecule biosynthetic process<br>regulation of transcription,	0.62<br>0.57	GO:0003700<br>GO:0003677	DNA-binding transcription factor activity<br>DNA binding	0.60	GO:0005634	nucleus			

			DNA-templated<br>0.43 GO:0009873 ethylene-activated signaling pathway			
Lus10038083	0.56	Myb-like protein I	0.59 GO:0016310 phosphorylation	0.61 GO:0016301 kinase activity		
Lus10032808	0.79	Inositol-tetrakisphosphate 1-kinase	0.85 GO:0032957 inositol trisphosphate metabolic process<br>0.59 GO:0016310 phosphorylation<br>0.46 GO:0006020 inositol metabolic process<br>0.39 GO:0009611 response to wounding	0.85 GO:0052726 inositol-1,3,4-trisphosphate 5-kinase activity<br>0.85 GO:0047325 inositol tetrakisphosphate 1-kinase activity<br>0.85 GO:0052725 inositol-1,3,4-trisphosphate 6-kinase activity<br>0.65 GO:0000287 magnesium ion binding<br>0.56 GO:0005524 ATP binding		0.85 EC:2.7.1.159 GO:0052726<br>0.85 KEGG:R03428 GO:0052726
Lus10032809	0.53	Putative Oxalate oxidase 2		0.43 GO:0016787 hydrolase activity	0.44 GO:0016021 integral component of membrane	0.43 EC:3.-.-.- GO:0016787
Lus10032830	0.52	HECT-type E3 ubiquitin transferase	0.71 GO:0016567 protein ubiquitination<br>0.50 GO:0045732 positive regulation of protein catabolic process<br>0.49 GO:0043161 proteasome-mediated ubiquitin-dependent protein catabolic process<br>0.34 GO:0006414 translational elongation	0.73 GO:0004842 ubiquitin-protein transferase activity<br>0.49 GO:0061659 ubiquitin-like protein ligase activity<br>0.39 GO:0016746 acyltransferase activity<br>0.36 GO:0016874 ligase activity<br>0.34 GO:0003746 translation elongation factor activity	0.39 GO:0005737 cytoplasm	0.39 EC:2.3.-.- GO:0016746<br>0.73 KEGG:R03876 GO:0004842
Lus10039905	0.0	Uncharacterized protein				
Lus10039900	0.97	protein GAMETE EXPRESSED 1	0.43 GO:0009553 embryo sac development<br>0.42 GO:0009555 pollen development<br>0.42 GO:0009793 embryo development ending in seed dormancy	0.40 GO:0042802 identical protein binding<br>0.34 GO:0008270 zinc ion binding<br>0.33 GO:0003676 nucleic acid binding	0.44 GO:0016021 integral component of membrane<br>0.36 GO:0005886 plasma membrane	
Lus10039904	0.56	Chalcone synthase	0.57 GO:0009813 flavonoid biosynthetic process<br>0.52 GO:0030639 polyketide biosynthetic process<br>0.37 GO:0080110 sporopollenin biosynthetic process	0.65 GO:0016747 acyltransferase activity, transferring groups other than amino-acyl groups<br>0.34 GO:0016853 isomerase activity	0.34 GO:0005783 endoplasmic reticulum	0.65 EC:2.3.1.- GO:0016747
Lus10039906	0.42	3-ketoacyl-CoA synthase	0.69 GO:0006633 fatty acid biosynthetic process<br>0.60 GO:0009409 response to cold<br>0.57 GO:0009416 response to light stimulus	0.78 GO:0102756 very-long-chain 3-ketoacyl-CoA synthase activity	0.43 GO:0016021 integral component of membrane	0.78 EC:2.3.1.199 GO:0102756
Lus10039902	0.0	Uncharacterized protein				
Lus10000463	0.64	TCP domain-containing protein	0.67 GO:2000112 regulation of cellular macromolecule biosynthetic process<br>0.58 GO:0006355 regulation of transcription, DNA-templated<br>0.44 GO:2000032 regulation of secondary shoot formation	0.62 GO:0003700 DNA-binding transcription factor activity<br>0.45 GO:0043565 sequence-specific DNA binding	0.42 GO:0005634 nucleus	
Lus10028516	0.80	probable calcium-binding protein CML23	0.38 GO:0080164 regulation of nitric oxide metabolic process<br>0.38 GO:0009909 regulation of flower development<br>0.33 GO:0016310 phosphorylation	0.69 GO:0005509 calcium ion binding<br>0.33 GO:0016301 kinase activity		
Lus10028520	0.48	pentatricopeptide repeat-containing protein At1g77360, mitochondrial-like		0.47 GO:0008270 zinc ion binding<br>0.41 GO:0003676 nucleic acid binding	0.44 GO:0016021 integral component of membrane	
Lus10028515	0.64	TFIIS N-terminal domain-	0.38 GO:0006414 translational elongation	0.38 GO:0003746 translation elongation factor	0.60 GO:0005634 nucleus	

		containing protein						activity						
Lus10028519	0.97	Polcalcin Nic t 1				0.69	GO:0005509	calcium ion binding	0.34<br>0.34<br>0.32	GO:0005737<br>GO:0012505<br>GO:0016020	cytoplasm<br>endomembrane system<br>membrane			
Lus10037721	0.59	GATA transcription factor 15	0.67<br>0.58	GO:2000112<br>GO:0006355	regulation of cellular macromolecule biosynthetic process<br>regulation of transcription, DNA-templated	0.67<br>0.64	GO:0043565<br>GO:0008270	sequence-specific DNA binding<br>zinc ion binding	0.32	GO:0016021	integral component of membrane			
Lus10037715	0.97	Pathogenesis-related protein class 10 (Fragment)	0.83<br>0.72<br>0.70<br>0.62<br>0.45<br>0.38<br>0.34	GO:0009738<br>GO:0043086<br>GO:0006952<br>GO:0009607<br>GO:0080163<br>GO:0090305<br>GO:0016070	abscisic acid-activated signaling pathway<br>negative regulation of catalytic activity<br>defense response<br>response to biotic stimulus<br>regulation of protein serine/threonine phosphatase activity<br>nucleic acid phosphodiester bond hydrolysis<br>RNA metabolic process	0.85<br>0.81<br>0.68<br>0.39<br>0.38<br>0.38<br>0.35<br>0.34	GO:0010427<br>GO:0004864<br>GO:0038023<br>GO:0044373<br>GO:0004518<br>GO:1904408<br>GO:0005509<br>GO:0140098	abscisic acid binding<br>protein phosphatase inhibitor activity<br>signaling receptor activity<br>cytokinin binding<br>nuclease activity<br>melatonin binding<br>calcium ion binding<br>catalytic activity, acting on RNA	0.39<br>0.38	GO:0005829<br>GO:0005634	cytosol<br>nucleus			
Lus10037719	0.45	Two-component response regulator	0.81<br>0.67<br>0.63<br>0.58<br>0.53<br>0.49<br>0.48<br>0.47<br>0.47<br>0.47	GO:0009736<br>GO:2000112<br>GO:0000160<br>GO:0006355<br>GO:0016310<br>GO:0090506<br>GO:1990110<br>GO:0080022<br>GO:0010380<br>GO:0010492	cytokinin-activated signaling pathway<br>regulation of cellular macromolecule biosynthetic process<br>phosphorelay signal transduction system<br>regulation of transcription, DNA-templated<br>phosphorylation<br>axillary shoot meristem initiation<br>callus formation<br>primary root development<br>regulation of chlorophyll biosynthetic process<br>maintenance of shoot apical meristem identity	0.62<br>0.57<br>0.54<br>0.43	GO:0003700<br>GO:0003677<br>GO:0016301<br>GO:0001067	DNA-binding transcription factor activity<br>DNA binding<br>kinase activity<br>transcription regulatory region nucleic acid binding	0.60	GO:0005634	nucleus			
Lus10037722	0.62	Importin subunit alpha	0.79	GO:0006606	protein import into nucleus	0.83<br>0.52<br>0.34	GO:0061608<br>GO:0008139<br>GO:0016746	nuclear import signal receptor activity<br>nuclear localization sequence binding<br>acyltransferase activity	0.51<br>0.42	GO:0005737<br>GO:0005634	cytoplasm<br>nucleus	0.34	EC:2.3.-.-	GO:0016746
Lus10015788	0.66	Cytochrome P450, family 78, subfamily A, polypeptide 5	0.34<br>0.34<br>0.33	GO:1901576<br>GO:0019438<br>GO:1901360	organic substance biosynthetic process<br>aromatic compound biosynthetic process<br>organic cyclic compound metabolic process	0.69<br>0.68<br>0.67<br>0.64	GO:0016705<br>GO:0004497<br>GO:0005506<br>GO:0020037	oxidoreductase activity, acting on paired donors, with incorporation or reduction of molecular oxygen<br>monooxygenase activity<br>iron ion binding<br>heme binding	0.42	GO:0016021	integral component of membrane	0.69	EC:1.14.-.-	GO:0016705
Lus10032193	0.76	methylsterol monooxygenase 1-1-like	0.63<br>0.49<br>0.46<br>0.41	GO:0008610<br>GO:0016125<br>GO:1901617<br>GO:1901362	lipid biosynthetic process<br>sterol metabolic process<br>organic hydroxy compound biosynthetic process<br>organic cyclic compound biosynthetic process	0.66<br>0.57<br>0.47	GO:0005506<br>GO:0004497<br>GO:0016705	iron ion binding<br>monooxygenase activity<br>oxidoreductase activity, acting on paired donors, with incorporation or reduction of molecular oxygen	0.46<br>0.44	GO:0005789<br>GO:0016021	endoplasmic reticulum membrane<br>integral component of membrane	0.47	EC:1.14.-.-	GO:0016705

Lus10002481	0.0	Uncharacterized protein				
Lus10002482	0.44	Calmodulin-domain kinase CDPK protein	0.64 GO:0006468 protein phosphorylation<br>0.49 GO:0018209 peptidyl-serine modification<br>0.42 GO:0035556 intracellular signal transduction	0.69 GO:0005509 calcium ion binding<br>0.64 GO:0004672 protein kinase activity<br>0.56 GO:0005524 ATP binding<br>0.48 GO:0005516 calmodulin binding	0.41 GO:0005634 nucleus<br>0.39 GO:0005886 plasma membrane<br>0.38 GO:0005737 cytoplasm	
Lus10002487	0.45	RING/U-box superfamily protein isoform 1	0.71 GO:0016567 protein ubiquitination	0.73 GO:0004842 ubiquitin-protein transferase activity<br>0.35 GO:0016874 ligase activity	0.44 GO:0016021 integral component of membrane	0.35 EC:6.-.-.- GO:0016874<br>0.73 KEGG:R03876 GO:0004842
Lus10002480	0.15	VARLMGL domain-containing protein				
Lus10002483	0.55	Octamer-binding transcription factor	0.67 GO:2000112 regulation of cellular macromolecule biosynthetic process<br>0.58 GO:0006355 regulation of transcription, DNA-templated	0.62 GO:0003700 DNA-binding transcription factor activity<br>0.56 GO:0003677 DNA binding<br>0.36 GO:0034256 chlorophyll(ide) b reductase activity	0.60 GO:0005634 nucleus	0.36 EC:1.1.1.294 GO:0034256
Lus10002491	0.41	peroxisome biogenesis factor 10 isoform X1	0.54 GO:0009789 positive regulation of abscisic acid-activated signaling pathway<br>0.51 GO:0016567 protein ubiquitination<br>0.50 GO:0009651 response to salt stress<br>0.50 GO:0009737 response to abscisic acid<br>0.46 GO:0006511 ubiquitin-dependent protein catabolic process	0.54 GO:0046872 metal ion binding<br>0.53 GO:0061630 ubiquitin protein ligase activity<br>0.36 GO:0016874 ligase activity<br>0.34 GO:0005515 protein binding	0.49 GO:0005829 cytosol<br>0.35 GO:0016021 integral component of membrane	0.36 EC:6.-.-.- GO:0016874
Lus10002478	0.0	Uncharacterized protein				
Lus10002492	0.85	Mitochondrial inner membrane protease subunit 2	0.84 GO:0006627 protein processing involved in protein targeting to mitochondrion<br>0.76 GO:0006465 signal peptide processing<br>0.50 GO:0033108 mitochondrial respiratory chain complex assembly<br>0.45 GO:0034622 protein-containing complex assembly	0.69 GO:0004252 serine-type endopeptidase activity	0.85 GO:0042720 mitochondrial inner membrane peptidase complex<br>0.34 GO:0016021 integral component of membrane	0.69 EC:3.4.19.1 EC:3.4.21.- EC:3.4.21.1 EC:3.4.21.10 EC:3.4.21.102 EC:3.4.21.20 EC:3.4.21.21 EC:3.4.21.22 EC:3.4.21.26 EC:3.4.21.27 EC:3.4.21.34 EC:3.4.21.35 EC:3.4.21.36 EC:3.4.21.38 EC:3.4.21.39 EC:3.4.21.4 EC:3.4.21.41 EC:3.4.21.42 EC:3.4.21.43 EC:3.4.21.45 EC:3.4.21.46 EC:3.4.21.47 EC:3.4.21.48 EC:3.4.21.5 EC:3.4.21.53 EC:3.4.21.54 EC:3.4.21.59 EC:3.4.21.6 EC:3.4.21.61 EC:3.4.21.62 EC:3.4.21.68 EC:3.4.21.69 EC:3.4.21.7 EC:3.4.21.71 EC:3.4.21.73 EC:3.4.21.75 EC:3.4.21.78 GO:0004252

													EC:3.4.21.79<br>EC:3.4.21.83<br>EC:3.4.21.87<br>EC:3.4.21.88<br>EC:3.4.21.89<br>EC:3.4.21.9<br>EC:3.4.21.92<br>EC:3.4.21.93<br>EC:3.4.21.94<br>EC:3.4.24.3<br>EC:3.4.24.34<br>EC:3.4.24.7	
Lus10002476	0.46	Pentatricopeptide repeat-containing protein												
Lus10036978	0.66	Trihelix transcription factor GT-1				0.57	GO:0003677	DNA binding						
Lus10036975	0.46	Pentatricopeptide repeat-containing protein, mitochondrial				0.55	GO:0016747	acyltransferase activity, transferring groups other than amino-acyl groups	0.40	GO:0016021	integral component of membrane	0.55	EC:2.3.1.-	GO:0016747
Lus10036977	0.81	NADH-ubiquinone reductase complex 1 MLRQ subunit							0.44<br>0.37<br>0.35	GO:0016021<br>GO:0042579<br>GO:0005829	integral component of membrane<br>microbody<br>cytosol			
Lus10040388	0.0	Uncharacterized protein	0.70<br>0.68<br>0.37<br>0.37<br>0.36<br>0.36	GO:0006952<br>GO:0009607<br>GO:0031348<br>GO:0009605<br>GO:0044419<br>GO:0010150	defense response<br>response to biotic stimulus<br>negative regulation of defense response<br>response to external stimulus<br>biological process involved in interspecies interaction between organisms<br>leaf senescence	0.77	GO:0005516	calmodulin binding	0.44<br>0.36<br>0.34<br>0.34<br>0.34	GO:0016021<br>GO:0009506<br>GO:0005794<br>GO:0005576<br>GO:0005886	integral component of membrane<br>plasmodesma<br>Golgi apparatus<br>extracellular region<br>plasma membrane			
Lus10040389	0.56	RING/U-box domain-containing protein, putative isoform 1	0.71	GO:0016567	protein ubiquitination	0.75<br>0.56	GO:0061630<br>GO:0008270	ubiquitin protein ligase activity<br>zinc ion binding						
Lus10040386	0.81	Serine carboxypeptidase-like 27 isoform 1	0.60	GO:0006508	proteolysis	0.71	GO:0004180	carboxypeptidase activity				0.71	EC:3.4.17.11	GO:0004180
Lus10040387	0.58	Carboxypeptidase	0.60<br>0.38	GO:0006508<br>GO:0009820	proteolysis<br>alkaloid metabolic process	0.74	GO:0004185	serine-type carboxypeptidase activity	0.65<br>0.33	GO:0005576<br>GO:0016021	extracellular region<br>integral component of membrane	0.74	EC:3.4.16.-<br>EC:3.4.16.2<br>EC:3.4.16.5<br>EC:3.4.16.6	GO:0004185
Lus10039422	0.58	Putative inactive receptor kinase	0.64<br>0.37<br>0.35<br>0.34<br>0.34<br>0.34	GO:0006468<br>GO:0050896<br>GO:0018212<br>GO:0050789<br>GO:0023052<br>GO:0007154	protein phosphorylation<br>response to stimulus<br>peptidyl-tyrosine modification<br>regulation of biological process<br>signaling<br>cell communication	0.64<br>0.56<br>0.35	GO:0004672<br>GO:0005524<br>GO:0004888	protein kinase activity<br>ATP binding<br>transmembrane signaling receptor activity	0.44	GO:0016021	integral component of membrane			
Lus10039419	0.47	HMA domain-containing protein				0.54	GO:0046872	metal ion binding	0.32	GO:0016021	integral component of membrane			
Lus10016837	0.54	Polygalacturonase	0.60	GO:0005975	carbohydrate metabolic process	0.80<br>0.37	GO:0004650<br>GO:0047911	polygalacturonase activity<br>galacturan 1,4-alpha-galacturonidase activity	0.34	GO:0016021	integral component of membrane	0.80	EC:3.2.1.15	GO:0004650

			0.36 GO:0016829 lyase activity		
Lus10016831	0.43 PPM-type phosphatase domain-containing protein	0.67 GO:0016311 dephosphorylation<br>0.35 GO:0006464 cellular protein modification process<br>0.35 GO:0016310 phosphorylation	0.68 GO:0016791 phosphatase activity<br>0.35 GO:0016301 kinase activity<br>0.34 GO:0140096 catalytic activity, acting on a protein	0.44 GO:0005829 cytosol<br>0.41 GO:0005634 nucleus	0.68 EC:3.1.3.- GO:0016791
Lus10016836	0.0 Uncharacterized protein				
Lus10016840	0.66 Major allergen Mal d 1	0.83 GO:0009738 abscisic acid-activated signaling pathway<br>0.72 GO:0043086 negative regulation of catalytic activity<br>0.70 GO:0006952 defense response<br>0.58 GO:0009607 response to biotic stimulus<br>0.56 GO:0080163 regulation of protein serine/threonine phosphatase activity	0.85 GO:0010427 abscisic acid binding<br>0.81 GO:0004864 protein phosphatase inhibitor activity<br>0.68 GO:0038023 signaling receptor activity	0.44 GO:0005634 nucleus<br>0.40 GO:0005737 cytoplasm	
Lus10018554	0.56 Hexosyltransferase	0.84 GO:0045489 pectin biosynthetic process<br>0.68 GO:0071555 cell wall organization<br>0.37 GO:0048363 mucilage pectin metabolic process<br>0.37 GO:0010192 mucilage biosynthetic process	0.84 GO:0047262 polygalacturonate 4-alpha-galacturonosyltransferase activity	0.72 GO:0000139 Golgi membrane<br>0.43 GO:0016021 integral component of membrane<br>0.37 GO:0000137 Golgi cis cisterna<br>0.35 GO:0009706 chloroplast inner membrane	0.84 EC:2.4.1.43 GO:0047262
Lus10018553	0.51 Major facilitator superfamily protein isoform 2	0.77 GO:0006857 oligopeptide transport<br>0.55 GO:0055085 transmembrane transport<br>0.44 GO:0006817 phosphate ion transport<br>0.41 GO:0009753 response to jasmonic acid<br>0.39 GO:0009611 response to wounding<br>0.35 GO:0042128 nitrate assimilation	0.57 GO:0022857 transmembrane transporter activity	0.44 GO:0016021 integral component of membrane	
Lus10018560	0.87 SAND domain-containing protein		0.57 GO:0003677 DNA binding<br>0.34 GO:0008270 zinc ion binding	0.46 GO:0005829 cytosol<br>0.43 GO:0005634 nucleus	
Lus10018563	0.62 Protein STRUBBELIG-RECEPTOR FAMILY 3 isoform B	0.64 GO:0006468 protein phosphorylation	0.64 GO:0004672 protein kinase activity<br>0.56 GO:0005524 ATP binding	0.44 GO:0016021 integral component of membrane	
Lus10002132	0.0 Uncharacterized protein				
Lus10002133	0.81 Early-responsive to dehydration stress protein isoform 2	0.61 GO:0098655 cation transmembrane transport	0.80 GO:0005227 calcium activated cation channel activity<br>0.42 GO:0008381 mechanosensitive ion channel activity	0.44 GO:0016021 integral component of membrane<br>0.42 GO:0005886 plasma membrane	
