# Supplementary material for: Identification of QTNs Associated With Flowering Time, Maturity, and Plant Height Traits in Linum usitatissimum L. Using Genome-Wide Association Study
Source: Front Genet. 2022 Jun 14;13:811924. doi: 10.3389/fgene.2022.811924 (PMC9237403; doi:10.3389/fgene.2022.811924)
Supplement: Supplementary file 3 [file Table1.DOCX]

**Supplementary table S1: List of linseed germplasm accessions used for association panel.**

| **S.N.** | **Accession** | **Geographical location** |
| --- | --- | --- |
| 1. | EC0000522 | Argentina |
| 2. | EC0001475 | Australia |
| 3. | IC0096624 | Maharashtra, India |
| 4. | IC0096628 | Maharashtra, India |
| 5. | IC0096631 | Maharashtra, India |
| 6. | IC0096632 | Maharashtra, India |
| 7. | IC0096633 | Maharashtra, India |
| 8. | IC0096634 | Maharashtra, India |
| 9. | IC0096637 | Maharashtra, India |
| 10. | IC0096638 | Maharashtra, India |
| 11. | IC0096647 | Maharashtra, India |
| 12. | IC0096648 | Maharashtra, India |
| 13. | IC0096657 | Maharashtra, India |
| 14. | IC0096690 | Maharashtra, India |
| 15. | EC0022648 | United State Of America |
| 16. | IC0096695 | Maharashtra, India |
| 17. | IC0096711 | Maharashtra, India |
| 18. | IC0096718 | Maharashtra, India |
| 19. | IC0096731 | Maharashtra, India |
| 20. | IC0096739 | Maharashtra, India |
| 21. | IC0267677 | Maharashtra, India |
| 22. | IC0268345 | Madhya Pradesh, India |
| 23. | IC0268350 | Madhya Pradesh, India |
| 24. | EC0041462 | Argentina |
| 25. | IC0280927 | Meghalaya, India |
| 26. | IC0283435 | Bihar, India |
| 27. | IC0305055 | Punjab, India |
| 28. | IC0319846 | Himachal Pradesh, India |
| 29. | IC0332123 | Bihar, India |
| 30. | IC0345397 | Maharashtra, India |
| 31. | IC0345409 | Maharashtra, India |
| 32. | EC0041465 | Argentina |
| 33. | IC0356381 | Uttar Pradesh, India |
| 34. | IC0384575 | Madhya Pradesh, India |
| 35. | IC0426929 | Uttar Pradesh, India |
| 36. | IC0426932 | Uttar Pradesh, India |
| 37. | EC0041466 | Argentina |
| 38. | IC0449071 | Chhattisgarh, India |
| 39. | IC0512310 | Chhattisgarh, India |
| 40. | IC0523804 | Rajasthan, India |
| 41. | IC0523807 | Released variety |
| 42. | EC0041467 | Argentina |
| 43. | IC0525910 | Uttar Pradesh, India |
| 44. | IC0525915 | Uttar Pradesh, India |
| 45. | IC0525917 | Uttar Pradesh, India |
| 46. | IC0525928 | Uttar Pradesh, India |
| 47. | IC0525939 | Uttar Pradesh, India |
| 48. | EC0041469 | Argentina |
| 49. | IC0526010 | Assam, India |
| 50. | IC0526023 | Himachal Pradesh, India |
| 51. | IC0526027 | Himachal Pradesh, India |
| 52. | EC0041481 | Argentina |
| 53. | IC0526031 | Himachal Pradesh, India |
| 54. | IC0526039 | Orissa, India |
| 55. | EC0041528 | Argentina |
| 56. | IC0526058 | Orissa, India |
| 57. | IC0526094 | West Bengal, India |
| 58. | IC0526118 | West Bengal, India |
| 59. | IC0526124 | Madhya Pradesh, India |
| 60. | IC0526130 | Maharashtra, India |
| 61. | IC0526151 | Madhya Pradesh, India |
| 62. | IC0526153 | Madhya Pradesh, India |
| 63. | EC0041535 | Argentina |
| 64. | IC0526154 | Chhattisgarh, India |
| 65. | IC0538726 | Himachal Pradesh, India |
| 66. | IC0538760 | Himachal Pradesh, India |
| 67. | IC0538795 | Himachal Pradesh, India |
| 68. | IC0554605 | Himachal Pradesh, India |
| 69. | IC0586944 | Maharashtra, India |
| 70. | IC0613907 | Chhattisgarh, India |
| 71. | IC0420772 | Released variety |
| 72. | IC0611328 | Released variety |
| 73. | EC0041619 | Argentina |
| 74. | EC0041621 | Argentina |
| 75. | EC0041622 | Argentina |
| 76. | EC0041643 | Argentina |
| 77. | EC0041644 | Argentina |
| 78. | EC0000526 | Argentina |
| 79. | EC0041650 | Argentina |
| 80. | EC0041700 | Argentina |
| 81. | EC0041720 | Argentina |
| 82. | EC0041734 | Argentina |
| 83. | EC0041753 | Argentina |
| 84. | EC0041764 | Argentina |
| 85. | EC0041765 | Argentina |
| 86. | EC0110289 | Romania |
| 87. | EC0000531 | Argentina |
| 88. | EC0115148 | United State Of America |
| 89. | EC0118743 | Hungary |
| 90. | EC0718826 | Australia |
| 91. | EC0718827 | Australia |
| 92. | EC0718828 | Australia |
| 93. | EC0718829 | Australia |
| 94. | EC0718830 | Australia |
| 95. | EC0718831 | Australia |
| 96. | EC0000538 | Argentina |
| 97. | EC0718850 | Australia |
| 98. | EC0718851 | Australia |
| 99. | IC0002376 | Unknown |
| 100. | IC0053278 | Madhya Pradesh, India |
| 101. | EC0000545 | Argentina |
| 102. | IC0054960 | Maharashtra, India |
| 103. | IC0054967 | Maharashtra, India |
| 104. | IC0054969 | Maharashtra, India |
| 105. | IC0054971 | West Bengal, India |
| 106. | IC0054972 | Maharashtra, India |
| 107. | IC0054976 | Maharashtra, India |
| 108. | IC0054986 | Maharashtra, India |
| 109. | IC0056365 | Maharashtra, India |
| 110. | EC0001388 | Australia |
| 111. | IC0061278 | Madhya Pradesh, India |
| 112. | IC0096463 | Maharashtra, India |
| 113. | IC0096479 | Maharashtra, India |
| 114. | IC0096485 | Maharashtra, India |
| 115. | IC0096487 | Maharashtra, India |
| 116. | IC0096488 | Maharashtra, India |
| 117. | IC0096489 | Maharashtra, India |
| 118. | IC0096494 | Maharashtra, India |
| 119. | IC0096511 | Maharashtra, India |
| 120. | IC0096514 | Maharashtra, India |
| 121. | IC0096534 | Maharashtra, India |
| 122. | IC0096539 | Maharashtra, India |
| 123. | IC0096549 | Maharashtra, India |
| 124. | IC0096555 | Maharashtra, India |
| 125. | EC0001432 | Australia |
| 126. | IC0096564 | Maharashtra, India |
| 127. | IC0096565 | Maharashtra, India |
| 128. | IC0096580 | Maharashtra, India |
| 129. | IC0096591 | Maharashtra, India |
| 130. | IC0096601 | Maharashtra, India |
| 131. | Kartika | Released variety |
