# Supplementary figures and images for: Identification of QTNs Associated With Flowering Time, Maturity, and Plant Height Traits in Linum usitatissimum L. Using Genome-Wide Association Study
Source: Front Genet. 2022 Jun 14;13:811924. doi: 10.3389/fgene.2022.811924 (PMC9237403; doi:10.3389/fgene.2022.811924)

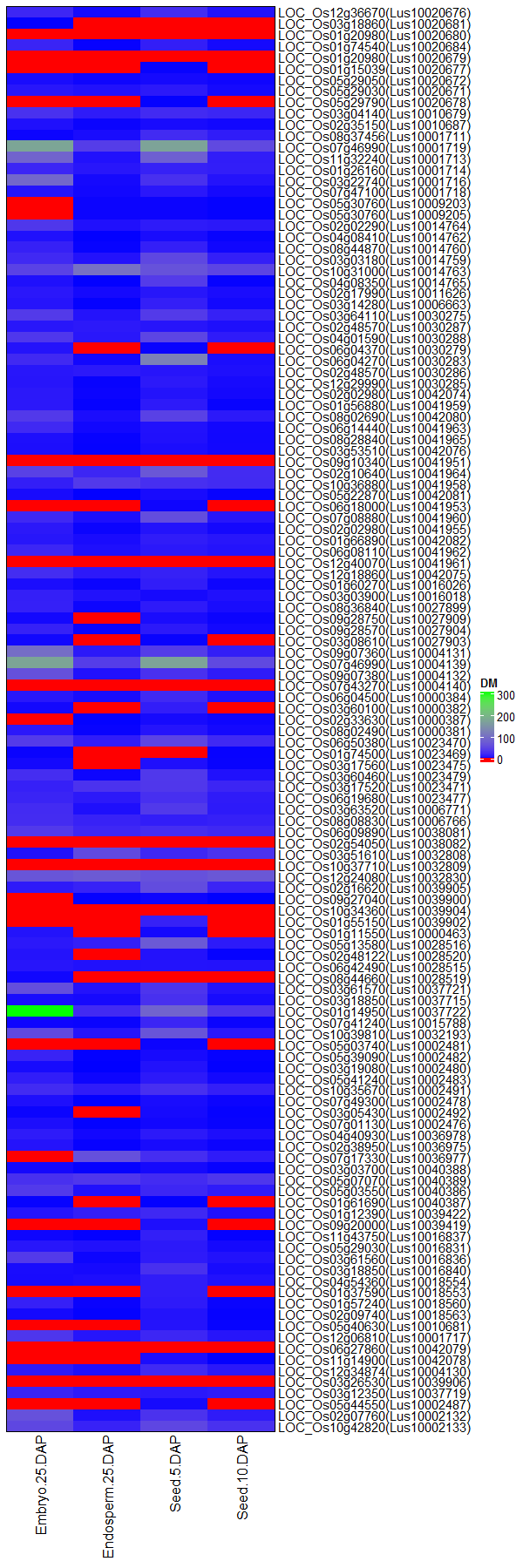

Supplement: Supplementary file 4 [file Image5.PNG]

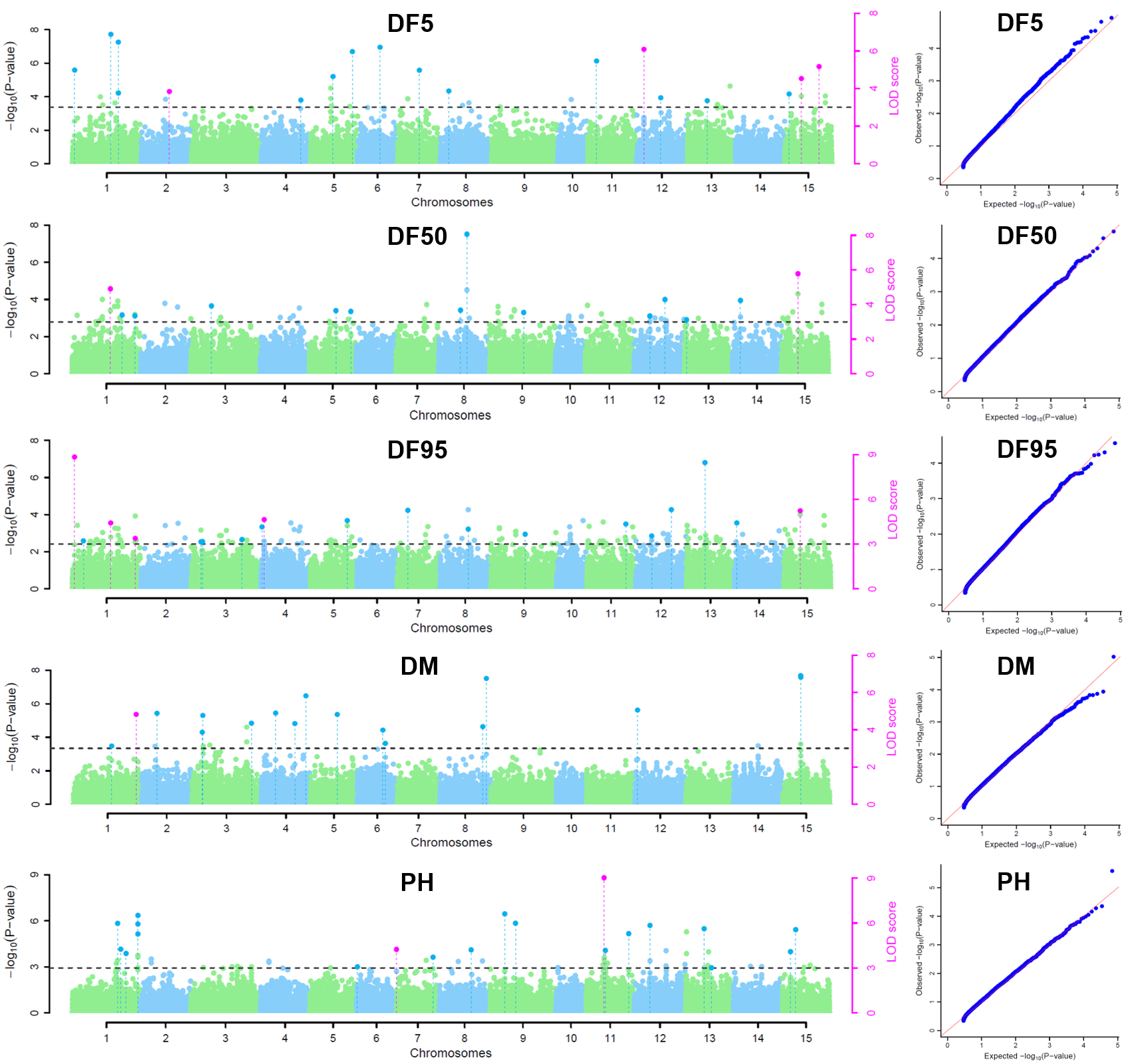

Supplement: Supplementary file 6 [file Image4.PNG]

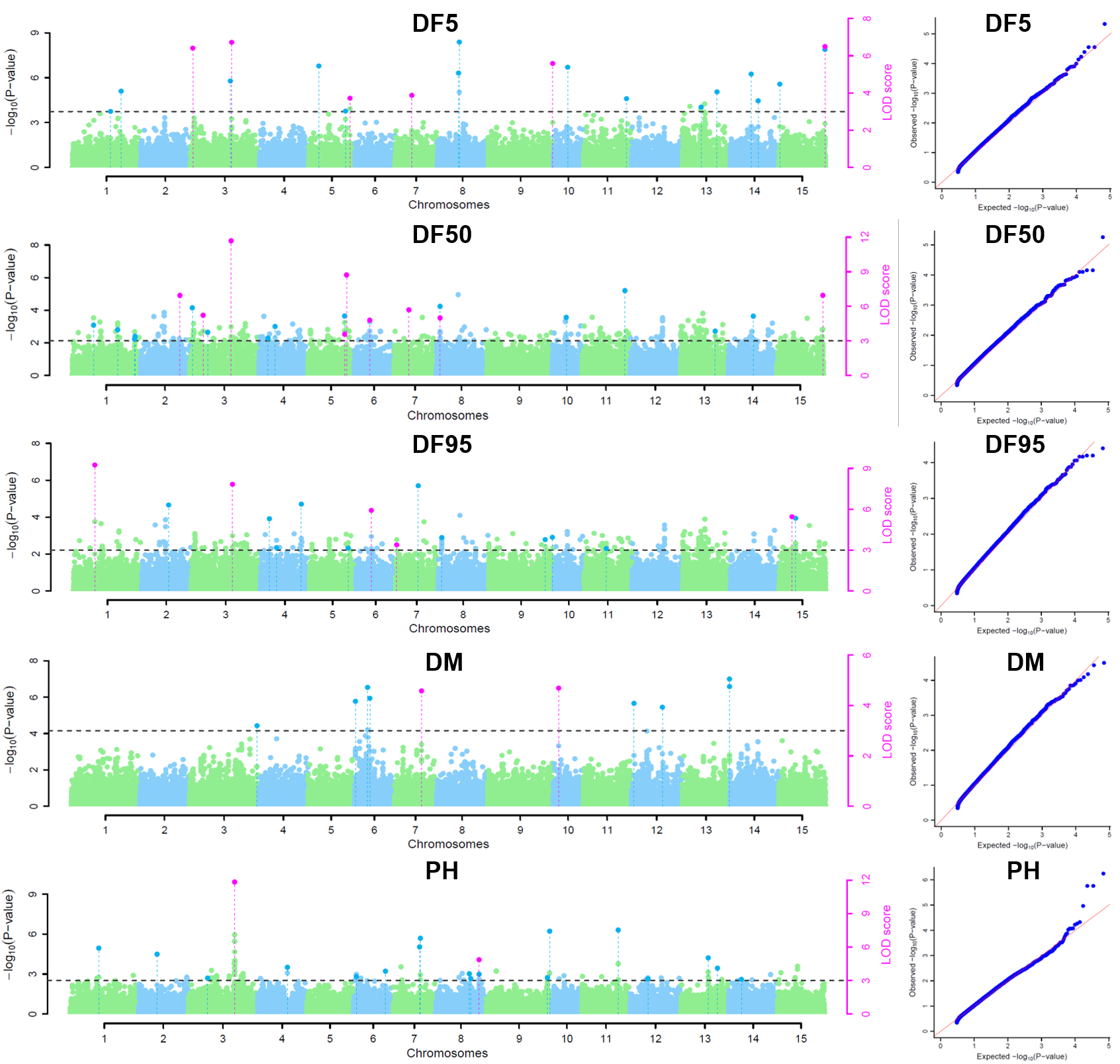

Supplement: Supplementary file 13 [file Image2.PNG]

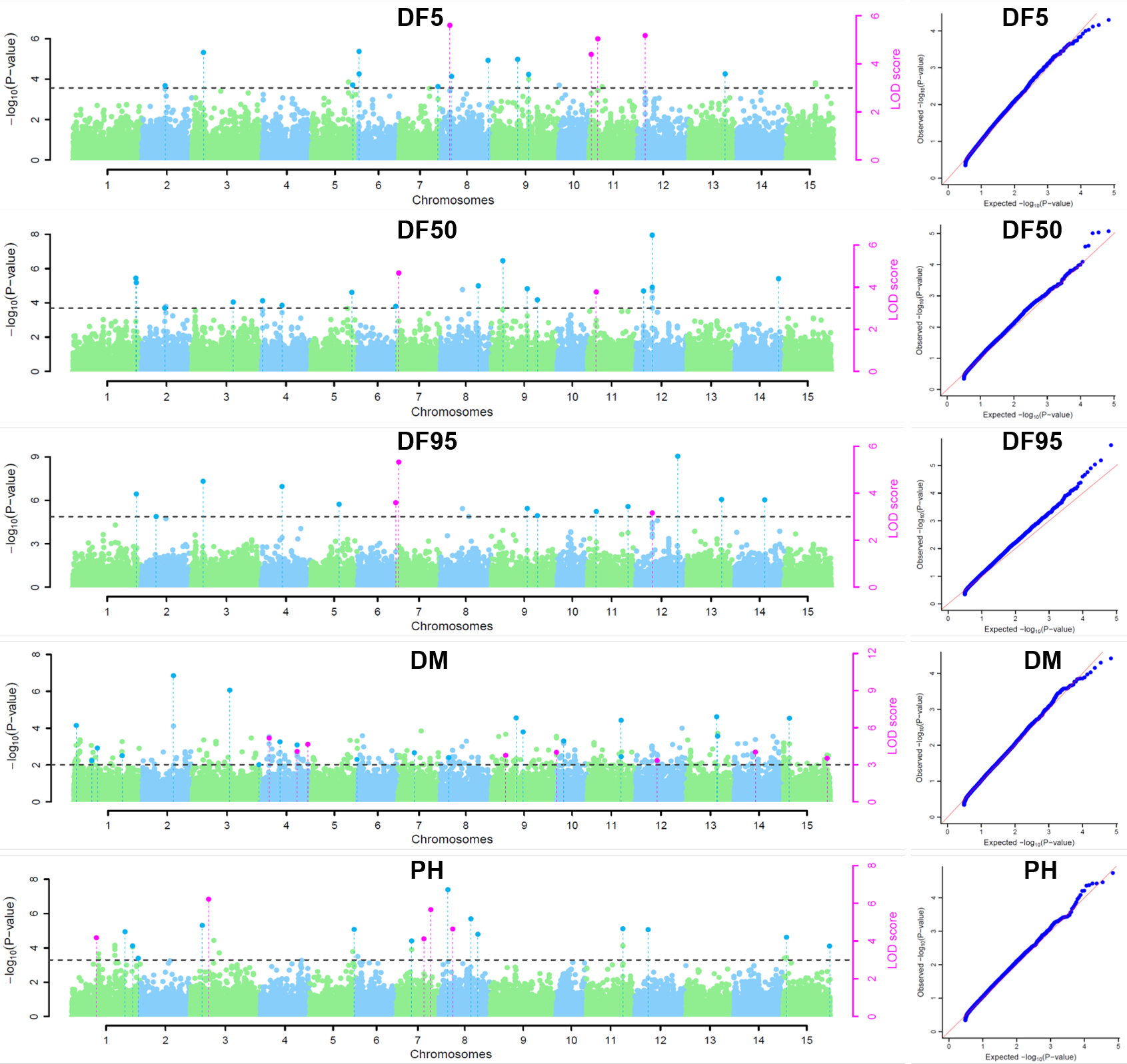

Supplement: Supplementary file 14 [file Image1.PNG]

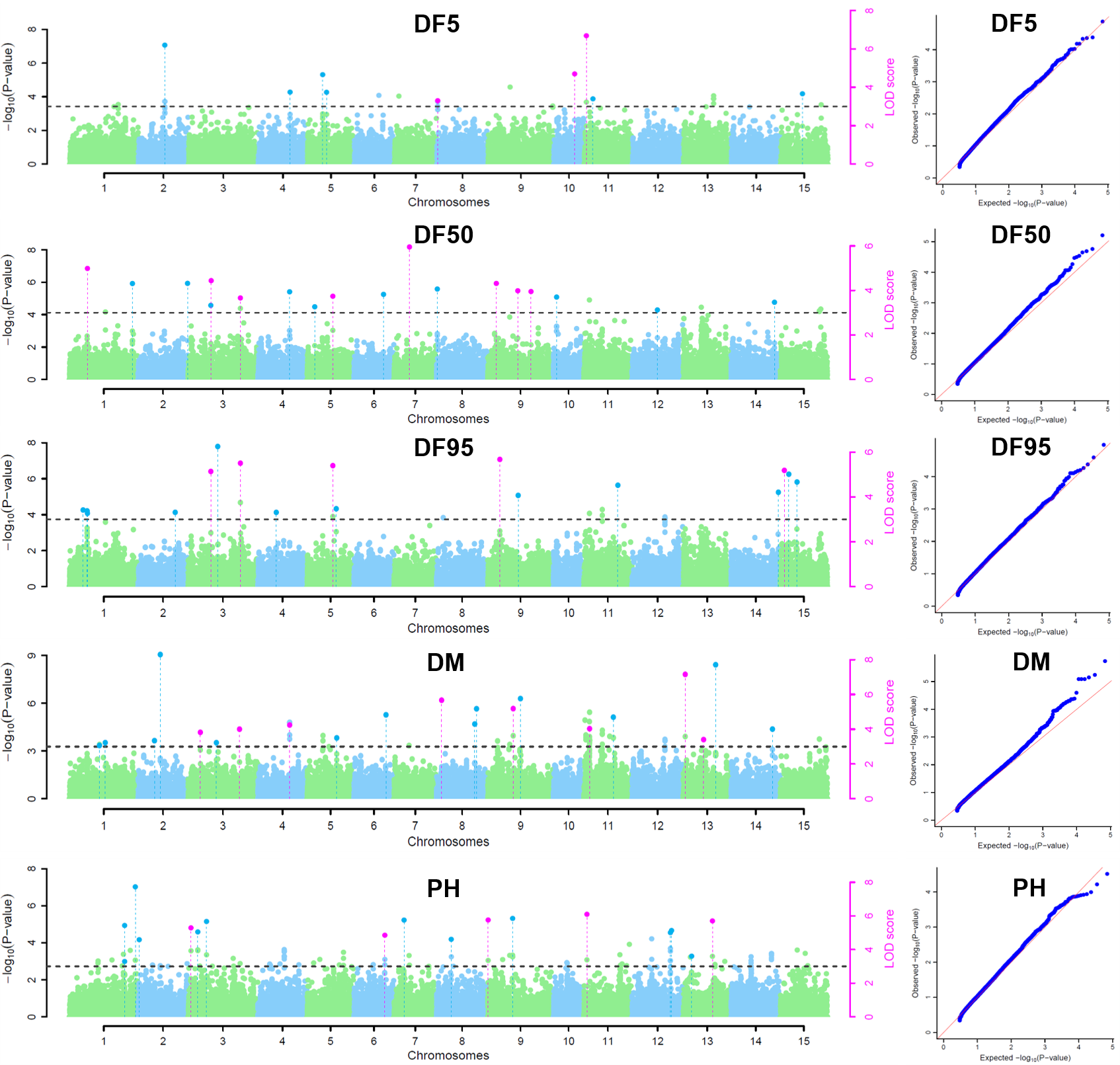

Supplement: Supplementary file 16 [file Image3.PNG]
