# Supplementary material for: Identification of QTNs Associated With Flowering Time, Maturity, and Plant Height Traits in Linum usitatissimum L. Using Genome-Wide Association Study
Source: Front Genet. 2022 Jun 14;13:811924. doi: 10.3389/fgene.2022.811924 (PMC9237403; doi:10.3389/fgene.2022.811924)
Supplement: Supplementary file 5 [file DataSheet3.PDF]

# Functional annotation of putative candidate genes for DF95

| Query header | Gene name Estimated PPV, Description | Biological process Estimated PPV, GO-id, Description | Molecular function Estimated PPV, GO-id, Description | Cellular component Estimated PPV, GO-id, Description | Inverse EC2GO, Kegg2GO |
|---|---|---|---|---|---|
| Lus10017320 | 0.0 Uncharacterized protein | | | | |
| Lus10036034 | 0.42 E3 ubiquitin-protein ligase RNF13 isoform X2 | 0.35 GO:0016310 phosphorylation | 0.54 GO:0046872 metal ion binding<br>0.35 GO:0016301 kinase activity<br>0.34 GO:0016874 ligase activity | | 0.34 EC:6.-.-.- GO:0016874 |
| Lus10016521 | 0.75 scarecrow-like protein 33 | 0.51 GO:2000112 regulation of cellular macromolecule biosynthetic process<br>0.45 GO:0006355 regulation of transcription, DNA-templated<br>0.42 GO:0009610 response to symbiotic fungus<br>0.36 GO:0005975 carbohydrate metabolic process | 0.51 GO:0043565 sequence-specific DNA binding<br>0.48 GO:0003700 DNA-binding transcription factor activity<br>0.38 GO:0004553 hydrolase activity, hydrolyzing O-glycosyl compounds | 0.59 GO:0005634 nucleus | 0.38 EC:3.2.1.- GO:0004553 |
| Lus10016528 | 0.71 3-ketoacyl-CoA synthase | 0.69 GO:0006633 fatty acid biosynthetic process<br>0.44 GO:0009409 response to cold<br>0.43 GO:0009416 response to light stimulus<br>0.37 GO:0090377 seed trichome initiation | 0.82 GO:0102756 very-long-chain 3-ketoacyl-CoA synthase activity | 0.44 GO:0016021 integral component of membrane<br>0.41 GO:0005783 endoplasmic reticulum | 0.82 EC:2.3.1.199 GO:0102756 |
| Lus10016529 | 0.61 PORR domain-containing protein | | 0.58 GO:0003723 RNA binding<br>0.35 GO:0016787 hydrolase activity | | 0.35 EC:3.-.-.- GO:0016787 |
| Lus10030162 | 0.67 Transducin/WD40 repeat-like superfamily protein isoform 1 | 0.86 GO:0010968 regulation of microtubule nucleation<br>0.62 GO:0000919 cell plate assembly<br>0.61 GO:2000694 regulation of phragmoplast microtubule organization<br>0.58 GO:0032467 positive regulation of cytokinesis<br>0.57 GO:0060236 regulation of mitotic spindle organization<br>0.53 GO:0009553 embryo sac development<br>0.52 GO:0009555 pollen development<br>0.36 GO:0009308 amine metabolic process<br>0.35 GO:0006470 protein dephosphorylation | 0.87 GO:0140496 gamma-tubulin complex binding<br>0.38 GO:0008131 primary amine oxidase activity<br>0.37 GO:0005507 copper ion binding<br>0.37 GO:0048038 quinone binding<br>0.35 GO:0106307 protein serine/threonine phosphatase activity<br>0.35 GO:0106306 protein serine/threonine phosphatase activity | 0.58 GO:0005828 kinetochore microtubule | 0.38 EC:1.4.3.21 GO:0008131<br>0.38 KEGG:R01853 GO:0008131 |
| Lus10030158 | 0.85 Delta-1-pyrroline-5-carboxylate synthase | 0.76 GO:0055129 L-proline biosynthetic process | 0.80 GO:0004349 glutamate 5-kinase activity | 0.51 GO:0005737 cytoplasm | 0.80 EC:2.7.2.11 GO:0004349<br>0.80 KEGG:R00239 GO:0004349 |

| | | | | | | |
|---|---|---|---|---|---|---|
| | | | 0.59 GO:0016310 phosphorylation | 0.80 GO:0004350 glutamate-5-semialdehyde dehydrogenase activity<br>0.56 GO:0005524 ATP binding | | |
| Lus10030167 | 0.45 | PPR domain-containing protein/PPR_1 domain-containing protein/PPR_2 domain-containing protein/PPR_3 domain-containing protein (Fragment) | | | 0.44 GO:0016021 integral component of membrane | |
| Lus10008264 | 0.92 | Agamous like-protein 11 | 0.75 GO:0045944 positive regulation of transcription by RNA polymerase II<br>0.67 GO:2000112 regulation of cellular macromolecule biosynthetic process<br>0.41 GO:0048316 seed development<br>0.37 GO:0010026 trichome differentiation<br>0.36 GO:0022412 cellular process involved in reproduction in multicellular organism | 0.77 GO:0000977 RNA polymerase II transcription regulatory region sequence-specific DNA binding<br>0.69 GO:0046983 protein dimerization activity<br>0.62 GO:0003700 DNA-binding transcription factor activity<br>0.42 GO:0000987 cis-regulatory region sequence-specific DNA binding | 0.60 GO:0005634 nucleus<br>0.35 GO:0016021 integral component of membrane | |
| Lus10010140 | 0.79 | desiccation protectant protein Lea14 homolog | 0.84 GO:0009269 response to desiccation | | 0.34 GO:0016021 integral component of membrane | |
| Lus10010126 | 0.93 | beclin-1-like protein isoform X1 | 0.76 GO:0006914 autophagy<br>0.49 GO:0006995 cellular response to nitrogen starvation<br>0.47 GO:0045324 late endosome to vacuole transport<br>0.46 GO:0007033 vacuole organization<br>0.43 GO:0070925 organelle assembly<br>0.37 GO:0006869 lipid transport | 0.37 GO:0008289 lipid binding | 0.50 GO:0034271 phosphatidylinositol 3-kinase complex, class III, type I<br>0.50 GO:0034272 phosphatidylinositol 3-kinase complex, class III, type II<br>0.46 GO:0000407 phagophore assembly site<br>0.32 GO:0016021 integral component of membrane | |
| Lus10010118 | 0.0 | Uncharacterized protein | | | | |
| Lus10010122 | 0.0 | Uncharacterized protein | 0.79 GO:0061458 reproductive system development | | 0.60 GO:0005634 nucleus | |
| Lus10010121 | 0.89 | Histone-lysine N-methyltransferase ATX3 | 0.77 GO:0034968 histone lysine methylation<br>0.47 GO:0060255 regulation of macromolecule metabolic process<br>0.38 GO:0035556 intracellular signal transduction | 0.78 GO:0018024 histone-lysine N-methyltransferase activity<br>0.54 GO:0046872 metal ion binding<br>0.37 GO:0003677 DNA binding | 0.59 GO:0005634 nucleus<br>0.32 GO:0016021 integral component of membrane | 0.78 EC:2.1.1.354 GO:0018024 |

| | | | | | | | | | | | | | | |
|---|---|---|---|---|---|---|---|---|---|---|---|---|---|---|
| Lus10010119 | 0.0 | Uncharacterized protein | | | | | | | | | | | | |
| Lus10010125 | 0.52 | Autophagy 6 isoform 1 | 0.67 | GO:0007049 | cell cycle | | | | 0.60<br>0.32 | GO:0005634<br>GO:0016021 | nucleus<br>integral component of membrane | | | |
| Lus10010123 | 0.0 | Uncharacterized protein | | | | | | | | | | | | |
| Lus10000367 | 0.68 | protein REDUCED WALL ACETYLATION 1-like isoform X2 | 0.59<br>0.42<br>0.38<br>0.36 | GO:0005975<br>GO:0009834<br>GO:0044038<br>GO:0043412 | carbohydrate metabolic process<br>plant-type secondary cell wall biogenesis<br>cell wall macromolecule biosynthetic process<br>macromolecule modification | 0.52 | GO:0016740 | transferase activity | 0.68<br>0.44 | GO:0005794<br>GO:0016021 | Golgi apparatus<br>integral component of membrane | 0.52 | EC:2.-.-.- | GO:0016740 |
| Lus10000986 | 0.50 | Protein CHUP1 chloroplastic | 0.77 | GO:0009658 | chloroplast organization | 0.45 | GO:0005525 | GTP binding | 0.79<br>0.33 | GO:0009707<br>GO:0016021 | chloroplast outer membrane<br>integral component of membrane | | | |
| Lus10000989 | 0.0 | Uncharacterized protein | | | | | | | | | | | | |
| Lus10037680 | 0.50 | RING/U-box | 0.57<br>0.56 | GO:0016567<br>GO:0006511 | protein ubiquitination<br>ubiquitin-dependent protein catabolic process | 0.64<br>0.44<br>0.41<br>0.35 | GO:0008270<br>GO:0097602<br>GO:0061630<br>GO:0016874 | zinc ion binding<br>cullin family protein binding<br>ubiquitin protein ligase activity<br>ligase activity | 0.42<br>0.38<br>0.32 | GO:0031461<br>GO:0005634<br>GO:0016021 | cullin-RING ubiquitin ligase complex<br>nucleus<br>integral component of membrane | 0.35 | EC:6.-.-.- | GO:0016874 |
| Lus10037685 | 0.64 | Serine/threonine-protein kinase SAPK2 | 0.64<br>0.42<br>0.41<br>0.40<br>0.36 | GO:0006468<br>GO:0007165<br>GO:0009845<br>GO:0071215<br>GO:0009651 | protein phosphorylation<br>signal transduction<br>seed germination<br>cellular response to abscisic acid stimulus<br>response to salt stress | 0.69<br>0.56<br>0.35<br>0.35 | GO:0004674<br>GO:0005524<br>GO:0106310<br>GO:0004712 | protein serine/threonine kinase activity<br>ATP binding<br>protein serine kinase activity<br>protein serine/threonine/tyrosine kinase activity | 0.36<br>0.35 | GO:0005634<br>GO:0005737 | nucleus<br>cytoplasm | 0.69 | EC:2.7.11.1 | GO:0004674 |
| Lus10013908 | 0.0 | Uncharacterized protein | | | | | | | 0.72 | GO:0005615 | extracellular space | | | |
| Lus10013910 | 0.40 | Non-specific serine/threonine protein kinase | | | | | | | | | | | | |
| Lus10013909 | 0.62 | DUF676 domain-containing protein | 0.56<br>0.49 | GO:0044255<br>GO:0009820 | cellular lipid metabolic process<br>alkaloid metabolic process | 0.44 | GO:0016787 | hydrolase activity | 0.39<br>0.33 | GO:0042579<br>GO:0016021 | microbody<br>integral component of membrane | 0.44 | EC:3.-.-.- | GO:0016787 |
| Lus10013905 | 0.70 | Dof zinc finger protein DOF1.5 | 0.67<br>0.58 | GO:2000112<br>GO:0006355 | regulation of cellular macromolecule biosynthetic process<br>regulation of transcription, DNA- | 0.57<br>0.40 | GO:0003677<br>GO:0003700 | DNA binding<br>DNA-binding transcription factor activity | 0.60 | GO:0005634 | nucleus | | | |

| | | | | | | | | | | | | | | |
|---|---|---|---|---|---|---|---|---|---|---|---|---|---|---|
| | | | | | templated | | | | | | | | | |
| Lus10033909 | 0.88 | Protein CHROMATIN REMODELING 8 | 0.50<br>0.37<br>0.36<br>0.35 | GO:0006283<br>GO:0032508<br>GO:0010332<br>GO:0006468 | transcription-coupled nucleotide-excision repair<br>DNA duplex unwinding<br>response to gamma radiation<br>protein phosphorylation | 0.56<br>0.47<br>0.40<br>0.36<br>0.35<br>0.33 | GO:0005524<br>GO:0008094<br>GO:0004386<br>GO:0004674<br>GO:0016787<br>GO:0003677 | ATP binding<br>ATP-dependent activity, acting on DNA<br>helicase activity<br>protein serine/threonine kinase activity<br>hydrolase activity<br>DNA binding | 0.43<br>0.34 | GO:0005634<br>GO:1990904 | nucleus<br>ribonucleoprotein complex | 0.36 | EC:2.7.11.1 | GO:0004674 |
| Lus10033881 | 0.64 | Autophagy-related protein 9 | 0.76<br>0.65<br>0.37 | GO:0006914<br>GO:0015031<br>GO:0032259 | autophagy<br>protein transport<br>methylation | 0.37<br>0.35 | GO:0008168<br>GO:0003676 | methyltransferase activity<br>nucleic acid binding | 0.72<br>0.69<br>0.44 | GO:0030659<br>GO:0005794<br>GO:0016021 | cytoplasmic vesicle membrane<br>Golgi apparatus<br>integral component of membrane | 0.37 | EC:2.1.1.- | GO:0008168 |
| Lus10033883 | 0.72 | Kinesin light chain 3 | | | | 0.52<br>0.43 | GO:0016740<br>GO:0003677 | transferase activity<br>DNA binding | | | | 0.52 | EC:2.-.-.- | GO:0016740 |
| Lus10036665 | 0.66 | LOB domain-containing protein | | | | | | | 0.44 | GO:0016021 | integral component of membrane | | | |
| Lus10036660 | 0.64 | Transcription factor MYB35 | 0.77<br>0.48<br>0.48<br>0.47 | GO:0010597<br>GO:0052545<br>GO:0055046<br>GO:0048658 | green leaf volatile biosynthetic process<br>callose localization<br>microgametogenesis<br>anther wall tapetum development | 0.68 | GO:0000976 | transcription cis-regulatory region binding | 0.39 | GO:0005634 | nucleus | | | |
| Lus10036668 | 0.59 | dnaJ homolog subfamily B member 1 | 0.69 | GO:0006457 | protein folding | 0.72<br>0.49 | GO:0051082<br>GO:0051087 | unfolded protein binding<br>chaperone binding | 0.45 | GO:0005829 | cytosol | | | |
| Lus10024412 | 0.73 | Origin recognition complex subunit 2 | 0.66 | GO:0006260 | DNA replication | 0.52<br>0.34 | GO:0003688<br>GO:0005515 | DNA replication origin binding<br>protein binding | 0.82<br>0.60<br>0.47 | GO:0000808<br>GO:0005634<br>GO:0070013 | origin recognition complex<br>nucleus<br>intracellular organelle lumen | | | |
| Lus10024417 | 0.53 | Actin-depolymerizing factor 2 | 0.83<br>0.74<br>0.37 | GO:0030042<br>GO:0043624<br>GO:0090378 | actin filament depolymerization<br>protein-containing complex disassembly<br>seed trichome elongation | 0.73<br>0.48 | GO:0003779<br>GO:0044877 | actin binding<br>protein-containing complex binding | 0.74<br>0.39 | GO:0015629<br>GO:0005737 | actin cytoskeleton<br>cytoplasm | | | |
| Lus10034282 | 0.67 | Mediator of RNA polymerase II transcription subunit 25 | 0.88<br>0.87<br>0.87<br>0.86<br>0.86<br>0.86 | GO:1905499<br>GO:0009911<br>GO:0010218<br>GO:0010091<br>GO:0010114<br>GO:0009867 | trichome papilla formation<br>positive regulation of flower development<br>response to far red light<br>trichome branching<br>response to red light<br>jasmonic acid mediated signaling pathway | 0.57 | GO:0003677 | DNA binding | 0.77<br>0.44 | GO:0016592<br>GO:0016021 | mediator complex<br>integral component of membrane | | | |

| | | | | | | | | | | | | | | |
|---|---|---|---|---|---|---|---|---|---|---|---|---|---|---|
| | | | 0.86 | GO:0009585 | red, far-red light phototransduction | | | | | | | | | |
| | | | 0.81 | GO:0050832 | defense response to fungus | | | | | | | | | |
| | | | 0.73 | GO:0031349 | positive regulation of defense response | | | | | | | | | |
| | | | 0.72 | GO:0045893 | positive regulation of transcription, DNA-templated | | | | | | | | | |
| Lus10026767 | 0.0 | Uncharacterized protein | | | | | | | | | | | | |
| Lus10026766 | 0.83 | Serrate RNA effector molecule | 0.69 | GO:0006397 | mRNA processing | | | | 0.60 | GO:0005634 | nucleus | | | |
| | | | 0.54 | GO:0031053 | primary miRNA processing | | | | 0.45 | GO:0070013 | intracellular organelle lumen | | | |
| | | | 0.45 | GO:2000011 | regulation of adaxial/abaxial pattern formation | | | | 0.43 | GO:0005846 | nuclear cap binding complex | | | |
| | | | 0.44 | GO:0010267 | primary ta-siRNA processing | | | | | | | | | |
| | | | 0.44 | GO:0031050 | ncRNA processing | | | | | | | | | |
| | | | 0.44 | GO:0048509 | regulation of meristem development | | | | | | | | | |
| | | | 0.42 | GO:0000381 | regulation of alternative mRNA splicing, via spliceosome | | | | | | | | | |
| | | | 0.42 | GO:0048367 | shoot system development | | | | | | | | | |
| | | | 0.40 | GO:0008380 | RNA splicing | | | | | | | | | |
| Lus10026773 | 0.88 | CCT-eta | 0.69 | GO:0006457 | protein folding | 0.72 | GO:0051082 | unfolded protein binding | 0.51 | GO:0005737 | cytoplasm | | | |
| | | | | | | 0.65 | GO:0016887 | ATP hydrolysis activity | 0.47 | GO:0101031 | chaperone complex | | | |
| | | | | | | 0.56 | GO:0005524 | ATP binding | | | | | | |
| Lus10026770 | 0.94 | F-box/WD-40 repeat-containing protein At5g21040 | 0.58 | GO:0016036 | cellular response to phosphate starvation | | | | 0.75 | GO:0030687 | preribosome, large subunit precursor | | | |
| | | | | | | | | | 0.45 | GO:0005634 | nucleus | | | |
| | | | | | | | | | 0.41 | GO:0005737 | cytoplasm | | | |
| Lus10026774 | 0.66 | NADH-cytochrome b5 reductase | 0.43 | GO:0022900 | electron transport chain | 0.83 | GO:0004128 | cytochrome-b5 reductase activity, acting on NAD(P)H | 0.44 | GO:0016021 | integral component of membrane | 0.83 | EC:1.6.2.2 | GO:0004128 |
| | | | | | | 0.46 | GO:0009703 | nitrate reductase (NADH) activity | | | | | | |
| Lus10023263 | 0.0 | Uncharacterized protein | | | | | | | | | | | | |
| Lus10023262 | 0.71 | GlcNAc kinase | 0.73 | GO:0046835 | carbohydrate phosphorylation | 0.85 | GO:0045127 | N-acetylglucosamine kinase activity | | | | 0.85 | EC:2.7.1.59 | GO:0045127 |
| | | | | | | | | | | | | 0.85 | KEGG:R01201 | GO:0045127 |
| Lus10023257 | 0.67 | Small RNA 2'-O-methyltransferase | 0.58 | GO:0032259 | methylation | 0.59 | GO:0008168 | methyltransferase activity | 0.44 | GO:0005634 | nucleus | 0.59 | EC:2.1.1.- | GO:0008168 |
| | | | 0.56 | GO:0034587 | piRNA metabolic process | 0.51 | GO:0003723 | RNA binding | 0.40 | GO:0005737 | cytoplasm | | | |
| | | | 0.56 | GO:0030422 | production of siRNA involved in RNA interference | 0.45 | GO:0140098 | catalytic activity, acting on RNA | | | | | | |
| | | | 0.46 | GO:0009451 | RNA modification | 0.43 | GO:0003755 | peptidyl-prolyl cis-trans isomerase activity | | | | | | |

| | | | | | | | | | | | | | | |
|---|---|---|---|---|---|---|---|---|---|---|---|---|---|---|
| | | | 0.46 | GO:0031050 | ncRNA processing | 0.39 | GO:0004399 | histidinol dehydrogenase activity | | | | | | |
| | | | 0.43 | GO:0000413 | protein peptidyl-prolyl isomerization | 0.37 | GO:0051287 | NAD binding | | | | | | |
| | | | 0.38 | GO:0000105 | histidine biosynthetic process | 0.35 | GO:0016791 | phosphatase activity | | | | | | |
| | | | 0.35 | GO:0016311 | dephosphorylation | 0.34 | GO:0046872 | metal ion binding | | | | | | |
| Lus10023256 | 0.18 | Putative tetratricopeptide-like helical domain, acetyltransferase A, auxiliary subunit | | | | 0.52 | GO:0016740 | transferase activity | | | | 0.52 | EC:2.-.-.- | GO:0016740 |
| Lus10023261 | 0.10 | GTP-binding protein (Fragment) | 0.37 | GO:1901001 | negative regulation of response to salt stress | 0.78 | GO:0043023 | ribosomal large subunit binding | 0.51 | GO:0005737 | cytoplasm | 0.34 | EC:6.-.-.- | GO:0016874 |
| | | | 0.36 | GO:0009651 | response to salt stress | 0.74 | GO:0043022 | ribosome binding | 0.32 | GO:0016021 | integral component of membrane | | | |
| | | | | | | 0.66 | GO:0005525 | GTP binding | | | | | | |
| | | | | | | 0.65 | GO:0016887 | ATP hydrolysis activity | | | | | | |
| | | | | | | 0.56 | GO:0005524 | ATP binding | | | | | | |
| | | | | | | 0.34 | GO:0016874 | ligase activity | | | | | | |
