# Supplementary material for: Identification of QTNs Associated With Flowering Time, Maturity, and Plant Height Traits in Linum usitatissimum L. Using Genome-Wide Association Study
Source: Front Genet. 2022 Jun 14;13:811924. doi: 10.3389/fgene.2022.811924 (PMC9237403; doi:10.3389/fgene.2022.811924)
Supplement: Supplementary file 8 [file Table2.DOCX]

**Supplementary table S2: Analysis of variance of DF5, DF50, DF95, DM and PH in five environments**

**Environment: AKOLA 2018-19**

| **Source** | **Df** | **DF5** | **DF50** | **DF95** | **DM** | **PH** |
| --- | --- | --- | --- | --- | --- | --- |
| **Block (ignoring treatments)** | 5 | 350.01 ** | 254.74 ** | 245.24 ** | 8.49 ns | 232.26 ** |
| **Treatment (eliminating blocks)** | 222 | 70.17 ** | 57.03 ** | 59.46 ** | 16.36 * | 52.73 ns |
| **Treatment: check** | 2 | 36.03 ns | 44.78 ns | 120.44 ** | 240.19 ** | 242.5 ** |
| **Treatment: test and test vs. check** | 220 | 70.48 ** | 57.15 ** | 58.9 ** | 14.33 * | 51 ns |
| **Residuals** | 28 | 11.86 | 13.63 | 18.33 | 7.93 | 32.3 |

ns P > 0.05; * P <= 0.05; ** P <= 0.01

**Environment: AKOLA 2019-20**

| **Source** | **Df** | **DF5** | **DF50** | **DF95** | **DM** |  | **PH** |  |
| --- | --- | --- | --- | --- | --- | --- | --- | --- |
| **Block (ignoring treatments)** | 5 | 100.82 ** | 81.22 ** | 69.48 ** | 10.83 ** |  | 303.24 ** |  |
| **Treatment (eliminating blocks)** | 222 | 27.92 ** | 22.01 * | 18.61 ** | 8.4 ** |  | 68.08 ** |  |
| **Treatment: check** | 2 | 242.11 ** | 137.25 ** | 124.08 ** | 47.19 ** |  | 550.9 ** |  |
| **Treatment: test and test vs. check** | 220 | 25.97 ** | 20.96 * | 17.65 * | 8.05 ** |  | 63.69 ** |  |
| **Residuals** | 28 | 11.94 | 10.82 | 8.39 | 2.52 |  | 24.47 |  |

ns P > 0.05; * P <= 0.05; ** P <= 0.01

**Environment: Delhi 2017-18**

| **Source** | **Df** | **DF5** | **DF50** | **DF95** | **DM** |  | **PH** |  |
| --- | --- | --- | --- | --- | --- | --- | --- | --- |
| **Block (ignoring treatments)** | 5 | 891.25 ** | 848.62 ** | 1021.58 ** | 158.34 ** |  | 654.98 ** |  |
| **Treatment (eliminating blocks)** | 222 | 136.39 ns | 177.84 ** | 203.29 ** | 43.45 ns |  | 109.35 ns |  |
| **Treatment: check** | 2 | 358.36 * | 302.11 ** | 316.75 ** | 197.33 ** |  | 825.93 ** |  |
| **Treatment: test and test vs. check** | 220 | 134.37 ns | 176.71 ** | 202.26 ** | 42.05 ns |  | 102.84 ns |  |
| **Residuals** | 28 | 81.66 | 51.66 | 29.07 | 34.24 |  | 91.19 |  |

ns P > 0.05; * P <= 0.05; ** P <= 0.01

**Environment: Delhi 2018-19**

| **Source** | **Df** | **DF5** | **DF50** | **DF95** | **DM** |  | **PH** |  |
| --- | --- | --- | --- | --- | --- | --- | --- | --- |
| **Block (ignoring treatments)** | 5 | 121.14 ** | 93.35 ** | 61.48 ** | 29.93 ** |  | 724.77 ** |  |
| **Treatment (eliminating blocks)** | 222 | 84.12 ** | 107.79 ** | 113.51 ** | 46.66 ** |  | 146.05 * |  |
| **Treatment: check** | 2 | 109.75 ** | 222.58 ** | 270.75 ** | 518.08 ** |  | 2149.17 ** |  |
| **Treatment: test and test vs. check** | 220 | 83.88 ** | 106.74 ** | 112.08 ** | 42.37 ** |  | 127.84 * |  |
| **Residuals** | 28 | 19.77 | 9.91 | 9.2 | 2.36 |  | 69.44 |  |

ns P > 0.05; * P <= 0.05; ** P <= 0.01

**Environment: Delhi 2019-20**

| **Source** | **Df** | **DF5** | **DF50** | **DF95** | **DM** |  | **PH** |  |
| --- | --- | --- | --- | --- | --- | --- | --- | --- |
| **Block (ignoring treatments)** | 5 | 799.62 ** | 850.67 ** | 775.48 ** | 42.79 ** |  | 589.44 ** |  |
| **Treatment (eliminating blocks)** | 222 | 156.42 ** | 141.59 ** | 150.13 ** | 31.69 ** |  | 100.09 ** |  |
| **Treatment: check** | 2 | 176.78 ns | 298.08 ** | 391.36 ** | 343.58 ** |  | 1373.46 ** |  |
| **Treatment: test and test vs. check** | 220 | 156.23 ** | 140.17 ** | 147.94 ** | 28.85 ** |  | 88.52 * |  |
| **Residuals** | 28 | 56.56 | 40.23 | 43.5 | 8.54 |  | 45.16 |  |

ns P > 0.05; * P <= 0.05; ** P <= 0.01
