# Supplementary material for: Identification of QTNs Associated With Flowering Time, Maturity, and Plant Height Traits in Linum usitatissimum L. Using Genome-Wide Association Study
Source: Front Genet. 2022 Jun 14;13:811924. doi: 10.3389/fgene.2022.811924 (PMC9237403; doi:10.3389/fgene.2022.811924)
Supplement: Supplementary file 9 [file DataSheet1.PDF]

Functional annotation of putative candidate genes for DF5

Query header	Gene name<br>Estimated PPV, Description	Biological process<br>Estimated PPV, GO-id, Description	Molecular function<br>Estimated PPV, GO-id, Description	Cellular component<br>Estimated PPV, GO-id, Description	Inverse EC2GO, Kegg2GO
Lus10024175	0.0 Uncharacterized protein		0.77 GO:0005516 calmodulin binding		
Lus10024180	0.77 Myosin heavy chain kinase B	0.58 GO:0016310 phosphorylation<br>0.38 GO:0032259 methylation	0.60 GO:0016301 kinase activity<br>0.38 GO:0008168 methyltransferase activity		0.38 EC:2.1.1.- GO:0008168
Lus10006490	0.95 THO complex subunit 5B			0.60 GO:0005634 nucleus	
Lus10006489	0.75 ABSCISIC ACID-INSENSITIVE 5-like protein 5	0.72 GO:0045893 positive regulation of transcription, DNA-templated<br>0.67 GO:2000112 regulation of cellular macromolecule biosynthetic process<br>0.36 GO:0009738 abscisic acid-activated signaling pathway	0.62 GO:0003700 DNA-binding transcription factor activity<br>0.34 GO:0005515 protein binding<br>0.33 GO:0003677 DNA binding	0.60 GO:0005634 nucleus	
Lus10007855	0.61 F-box/kelch-repeat protein At3g61590			0.44 GO:0016021 integral component of membrane	
Lus10007856	0.46 1-phosphatidylinositol-4-phosphate 5-kinase	0.76 GO:0046854 phosphatidylinositol phosphate biosynthetic process<br>0.59 GO:0016310 phosphorylation	0.85 GO:0016308 1-phosphatidylinositol-4-phosphate 5-kinase activity<br>0.56 GO:0005524 ATP binding		0.85 EC:2.7.1.68 GO:0016308<br>0.85 KEGG:R03469 GO:0016308
Lus10042079	0.0 Uncharacterized protein				
Lus10042078	0.80 Vignain	0.60 GO:0006508 proteolysis<br>0.48 GO:0044257 cellular protein catabolic process	0.72 GO:0008234 cysteine-type peptidase activity<br>0.46 GO:0004175 endopeptidase activity<br>0.36 GO:0032440 2-alkenal reductase [NAD(P)+] activity	0.50 GO:0005764 lysosome<br>0.49 GO:0005615 extracellular space<br>0.37 GO:0005788 endoplasmic reticulum lumen<br>0.37 GO:0033095 aleurone grain<br>0.32 GO:0016021 integral component of membrane	0.46 EC:3.4.99.- GO:0004175
Lus10008264	0.92 Agamous like-protein 11	0.75 GO:0045944 positive regulation of transcription by RNA polymerase II<br>0.67 GO:2000112 regulation of cellular macromolecule biosynthetic process<br>0.41 GO:0048316 seed development<br>0.37 GO:0010026 trichome differentiation	0.77 GO:0000977 RNA polymerase II transcription regulatory region sequence-specific DNA binding<br>0.69 GO:0046983 protein dimerization activity<br>0.62 GO:0003700 DNA-binding transcription factor activity	0.60 GO:0005634 nucleus<br>0.35 GO:0016021 integral component of membrane	

			0.36	GO:0022412	cellular process involved in reproduction in multicellular organism	0.42	GO:0000987	cis-regulatory region sequence-specific DNA binding						
Lus10013395	0.57	Carboxypeptidase	0.60	GO:0006508	proteolysis	0.73	GO:0004185	serine-type carboxypeptidase activity	0.64<br>0.33	GO:0005576<br>GO:0016021	extracellular region<br>integral component of membrane	0.73	EC:3.4.16.-<br>EC:3.4.16.2<br>EC:3.4.16.5<br>EC:3.4.16.6	GO:0004185
Lus10021863	0.78	L-ascorbate oxidase homolog				0.73<br>0.55	GO:0005507<br>GO:0016491	copper ion binding<br>oxidoreductase activity				0.55	EC:1.-.-.-	GO:0016491
Lus10021864	0.80	L-ascorbate oxidase homolog				0.73<br>0.55	GO:0005507<br>GO:0016491	copper ion binding<br>oxidoreductase activity				0.55	EC:1.-.-.-	GO:0016491
Lus10001757	0.68	serine/threonine-protein kinase AGC1-7-like isoform X2	0.59	GO:0006468	protein phosphorylation	0.61<br>0.58<br>0.54<br>0.52	GO:0016301<br>GO:0016773<br>GO:0140096<br>GO:0005524	kinase activity<br>phosphotransferase activity, alcohol group as acceptor<br>catalytic activity, acting on a protein<br>ATP binding				0.58	EC:2.7.1.-	GO:0016773
Lus10031994	0.48	putative pentatricopeptide repeat-containing protein At2g01510	0.42<br>0.34	GO:0009451<br>GO:0090305	RNA modification<br>nucleic acid phosphodiester bond hydrolysis	0.64<br>0.40<br>0.34	GO:0008270<br>GO:0003723<br>GO:0004519	zinc ion binding<br>RNA binding<br>endonuclease activity	0.40<br>0.33	GO:0043231<br>GO:0005737	intracellular membrane-bounded organelle<br>cytoplasm			
Lus10031993	0.46	RING finger protein 10	0.46<br>0.43<br>0.43<br>0.39<br>0.39<br>0.38	GO:0045944<br>GO:2000112<br>GO:0044260<br>GO:0034976<br>GO:0044238<br>GO:0006457	positive regulation of transcription by RNA polymerase II<br>regulation of cellular macromolecule biosynthetic process<br>cellular macromolecule metabolic process<br>response to endoplasmic reticulum stress<br>primary metabolic process<br>protein folding	0.54<br>0.46<br>0.40<br>0.36	GO:0046872<br>GO:0000976<br>GO:0016864<br>GO:0140096	metal ion binding<br>transcription cis-regulatory region binding<br>intramolecular oxidoreductase activity, transposing S-S bonds<br>catalytic activity, acting on a protein	0.51<br>0.37<br>0.35<br>0.32	GO:0005737<br>GO:0012505<br>GO:0043231<br>GO:0016021	cytoplasm<br>endomembrane system<br>intracellular membrane-bounded organelle<br>integral component of membrane	0.40	EC:5.3.4.-	GO:0016864
Lus10031992	0.97	sialyltransferase-like protein 1				0.65	GO:0016757	glycosyltransferase activity				0.65	EC:2.4.-.-	GO:0016757
Lus10031991	0.86	Protein AUXIN SIGNALING F-BOX 3	0.79<br>0.53	GO:0009734<br>GO:0031146	auxin-activated signaling pathway<br>SCF-dependent proteasomal ubiquitin-dependent protein catabolic process	0.44	GO:0000822	inositol hexakisphosphate binding	0.53	GO:0019005	SCF ubiquitin ligase complex			

Lus10006587	0.58	transcription factor UNE12-like	0.44	GO:0006357	regulation of transcription by RNA polymerase II	0.69	GO:0046983	protein dimerization activity	0.60	GO:0005634	nucleus			
			0.44	GO:2000112	regulation of cellular macromolecule biosynthetic process	0.47	GO:0000978	RNA polymerase II cis-regulatory region sequence-specific DNA binding						
						0.45	GO:0000981	DNA-binding transcription factor activity, RNA polymerase II-specific						
Lus10006590	0.84	Light-inducible protein CPRF2	0.67	GO:2000112	regulation of cellular macromolecule biosynthetic process	0.62	GO:0003700	DNA-binding transcription factor activity	0.60	GO:0005634	nucleus			
			0.58	GO:0006355	regulation of transcription, DNA-templated	0.40	GO:0043621	protein self-association						
			0.41	GO:0009649	entrainment of circadian clock	0.39	GO:0019900	kinase binding						
			0.41	GO:0071333	cellular response to glucose stimulus	0.39	GO:0046982	protein heterodimerization activity						
			0.40	GO:0071215	cellular response to abscisic acid stimulus	0.38	GO:0042802	identical protein binding						
			0.39	GO:0009267	cellular response to starvation	0.34	GO:0016301	kinase activity						
			0.33	GO:0016310	phosphorylation	0.33	GO:0003677	DNA binding						
Lus10002501	0.0	Uncharacterized protein												
Lus10002500	0.55	Glycerol-3-phosphate 2-O-acyltransferase 6	0.56	GO:0010143	cutin biosynthetic process	0.63	GO:0016746	acyltransferase activity	0.44	GO:0016021	integral component of membrane	0.63	EC:2.3.-.-	GO:0016746
			0.46	GO:0016311	dephosphorylation	0.47	GO:0016791	phosphatase activity						
			0.45	GO:0009908	flower development									
Lus10033912	0.59	GRAS family transcription factor	0.56	GO:2000112	regulation of cellular macromolecule biosynthetic process	0.56	GO:0043565	sequence-specific DNA binding	0.60	GO:0005634	nucleus			
			0.49	GO:0006355	regulation of transcription, DNA-templated	0.52	GO:0003700	DNA-binding transcription factor activity						
Lus10033883	0.72	Kinesin light chain 3				0.52	GO:0016740	transferase activity				0.52	EC:2.-.-.-	GO:0016740
						0.43	GO:0003677	DNA binding						
Lus10040255	0.57	Putative small GTPase superfamily, P-loop containing nucleoside triphosphate hydrolase				0.68	GO:0003924	GTPase activity	0.47	GO:0005768	endosome			
						0.66	GO:0005525	GTP binding	0.43	GO:0005794	Golgi apparatus			
									0.32	GO:0016021	integral component of membrane			
Lus10040258	0.33	LIM zinc-binding domain-containing protein				0.64	GO:0043130	ubiquitin binding						
						0.54	GO:0046872	metal ion binding						
Lus10040256														

	0.37	Homeobox domain-containing protein	0.65	GO:2000112	regulation of cellular macromolecule biosynthetic process	0.57	GO:0000978	RNA polymerase II cis-regulatory region sequence-specific DNA binding	0.58	GO:0005634	nucleus			
			0.56	GO:0006355	regulation of transcription, DNA-templated	0.54	GO:0000981	DNA-binding transcription factor activity, RNA polymerase II-specific						
Lus10001784	0.51	Pentatricopeptide repeat-containing protein							0.60	GO:0005739	mitochondrion			
									0.37	GO:0016021	integral component of membrane			
Lus10001783	0.45	Serine/threonine-protein kinase Nek2	0.64	GO:0006468	protein phosphorylation	0.64	GO:0004672	protein kinase activity	0.37	GO:0055028	cortical microtubule			
			0.36	GO:0000165	MAPK cascade	0.55	GO:0005524	ATP binding	0.33	GO:0016021	integral component of membrane			
			0.35	GO:0007017	microtubule-based process									
Lus10026767	0.0	Uncharacterized protein												
Lus10026770	0.94	F-box/WD-40 repeat-containing protein At5g21040	0.58	GO:0016036	cellular response to phosphate starvation				0.75	GO:0030687	preribosome, large subunit precursor			
									0.45	GO:0005634	nucleus			
									0.41	GO:0005737	cytoplasm			
Lus10023257	0.67	Small RNA 2'-O-methyltransferase	0.58	GO:0032259	methylation	0.59	GO:0008168	methyltransferase activity	0.44	GO:0005634	nucleus	0.59	EC:2.1.1.-	GO:0008168
			0.56	GO:0034587	piRNA metabolic process	0.51	GO:0003723	RNA binding	0.40	GO:0005737	cytoplasm			
			0.56	GO:0030422	production of siRNA involved in RNA interference	0.45	GO:0140098	catalytic activity, acting on RNA						
			0.46	GO:0009451	RNA modification	0.43	GO:0003755	peptidyl-prolyl cis-trans isomerase activity						
			0.46	GO:0031050	ncRNA processing	0.39	GO:0004399	histidinol dehydrogenase activity						
			0.43	GO:0000413	protein peptidyl-prolyl isomerization	0.37	GO:0051287	NAD binding						
			0.38	GO:0000105	histidine biosynthetic process	0.35	GO:0016791	phosphatase activity						
			0.35	GO:0016311	dephosphorylation	0.34	GO:0046872	metal ion binding						
Lus10023256	0.18	Putative tetratricopeptide-like helical domain, acetyltransferase A, auxiliary subunit				0.52	GO:0016740	transferase activity				0.52	EC:2.-.-.-	GO:0016740
Lus10023259	0.11	Geranylgeranyl diphosphate synthase, type II	0.70	GO:0008299	isoprenoid biosynthetic process									
Lus10042602	0.10	PHD-type domain-containing protein	0.74	GO:2000113	negative regulation of cellular macromolecule biosynthetic process	0.79	GO:0003714	transcription corepressor activity						
			0.71	GO:0045892	negative regulation of transcription,	0.57	GO:0003677	DNA binding						
						0.54	GO:0046872	metal ion binding						

			DNA-templated			
Lus10042604	0.63	Late embryogenesis abundant protein D-34				
