# Supplementary material for: Identification of QTNs Associated With Flowering Time, Maturity, and Plant Height Traits in Linum usitatissimum L. Using Genome-Wide Association Study
Source: Front Genet. 2022 Jun 14;13:811924. doi: 10.3389/fgene.2022.811924 (PMC9237403; doi:10.3389/fgene.2022.811924)
Supplement: Supplementary file 10 [file DataSheet5.PDF]

Functional annotation of putative candidate genes for PH

Query header	Gene name<br>Estimated PPV, Description	Biological process<br>Estimated PPV, GO-id, Description	Molecular function<br>Estimated PPV, GO-id, Description	Cellular component<br>Estimated PPV, GO-id, Description	Inverse EC2GO, Kegg2GO
Lus10019185	0.0 Uncharacterized protein				
Lus10019186	0.0 Uncharacterized protein				
Lus10019184	0.75 Cytochrome P450, family 72, subfamily A, polypeptide 9		0.69 GO:0016705 oxidoreductase activity, acting on paired donors, with incorporation or reduction of molecular oxygen<br>0.68 GO:0004497 monooxygenase activity<br>0.67 GO:0005506 iron ion binding<br>0.64 GO:0020037 heme binding	0.41 GO:0016021 integral component of membrane	0.69 EC:1.14.-.- GO:0016705
Lus10019188	0.0 Uncharacterized protein				
Lus10014401	0.47 Glycosyltransferase	0.51 GO:0009718 anthocyanin-containing compound biosynthetic process<br>0.46 GO:0006657 CDP-choline pathway	0.72 GO:0008194 UDP-glycosyltransferase activity<br>0.48 GO:0004142 diacylglycerol cholinephosphotransferase activity<br>0.48 GO:0016758 hexosyltransferase activity		0.48 EC:2.7.8.2 GO:0004142
Lus10014394	0.0 Uncharacterized protein				
Lus10014400	0.72 Caffeic acid 3-O-methyltransferase	0.63 GO:0032259 methylation<br>0.57 GO:1901847 nicotinate metabolic process<br>0.41 GO:0019438 aromatic compound biosynthetic process	0.73 GO:0008171 O-methyltransferase activity<br>0.60 GO:0008757 S-adenosylmethionine-dependent methyltransferase activity<br>0.58 GO:0046983 protein dimerization activity<br>0.47 GO:0008170 N-methyltransferase activity	0.39 GO:0005829 cytosol	0.73 EC:2.1.1.- GO:0008171
Lus10014399	0.75 Carotene epsilon-monooxygenase	0.48 GO:0016114 terpenoid biosynthetic process<br>0.47 GO:0016108 tetraterpenoid metabolic process<br>0.44 GO:0046148 pigment biosynthetic process<br>0.37 GO:0019438 aromatic compound biosynthetic process<br>0.36 GO:1901362 organic cyclic compound biosynthetic process	0.69 GO:0016705 oxidoreductase activity, acting on paired donors, with incorporation or reduction of molecular oxygen<br>0.68 GO:0004497 monooxygenase activity<br>0.67 GO:0005506 iron ion binding<br>0.64 GO:0020037 heme binding	0.36 GO:0016021 integral component of membrane	0.69 EC:1.14.-.- GO:0016705
Lus10003905	0.0 Uncharacterized protein				
Lus10003908	0.0 Uncharacterized protein				
Lus10003903	0.96 probable GABA transporter 2	0.69 GO:0006865 amino acid transport<br>0.57 GO:0009734 auxin-activated signaling pathway<br>0.44 GO:0055085 transmembrane transport	0.53 GO:0015293 symporter activity<br>0.33 GO:0003677 DNA binding	0.44 GO:0016021 integral component of membrane<br>0.40 GO:0005886 plasma membrane<br>0.33 GO:0005634 nucleus	
Lus10018947	0.55 Embryo defective				

Lus10018944	0.55	probable serine/threonine-protein kinase PBL5 isoform X1	0.64<br>0.57	GO:0006468<br>GO:0018212	protein phosphorylation<br>peptidyl-tyrosine modification	0.64<br>0.56	GO:0004672<br>GO:0005524	protein kinase activity<br>ATP binding	0.53<br>0.33	GO:0005886<br>GO:0016021	plasma membrane<br>integral component of membrane			
Lus10018946	0.39	DEAD-box ATP-dependent RNA helicase 39-like	0.42<br>0.37	GO:1902775<br>GO:0034622	mitochondrial large ribosomal subunit assembly<br>protein-containing complex assembly	0.68<br>0.56<br>0.56<br>0.50	GO:0003724<br>GO:0003723<br>GO:0005524<br>GO:0016787	RNA helicase activity<br>RNA binding<br>ATP binding<br>hydrolase activity	0.36<br>0.36<br>0.34	GO:0043231<br>GO:1990904<br>GO:0005737	intracellular membrane-bounded organelle<br>ribonucleoprotein complex<br>cytoplasm	0.68	EC:3.6.4.13	GO:0003724
Lus10018945	0.0	Uncharacterized protein												
Lus10000613	0.67	LOB domain-containing protein	0.76<br>0.72<br>0.67	GO:0009755<br>GO:0045893<br>GO:2000112	hormone-mediated signaling pathway<br>positive regulation of transcription, DNA-templated<br>regulation of cellular macromolecule biosynthetic process				0.60	GO:0005634	nucleus			
Lus10005957	0.0	Uncharacterized protein												
Lus10005952	0.83	Protein trichome birefringence	0.35	GO:0009247	glycolipid biosynthetic process	0.53<br>0.35	GO:0016413<br>GO:0016758	O-acetyltransferase activity<br>hexosyltransferase activity	0.49<br>0.44<br>0.35	GO:0005794<br>GO:0016021<br>GO:0005789	Golgi apparatus<br>integral component of membrane<br>endoplasmic reticulum membrane	0.35	EC:2.4.1.-	GO:0016758
Lus10015710	0.11	C2 calcium/lipid-binding plant phosphoribosyltransferase family protein				0.65	GO:0016757	glycosyltransferase activity	0.44	GO:0016021	integral component of membrane	0.65	EC:2.4.-.-	GO:0016757
Lus10035590	0.52	GDSL esterase/lipase	0.41	GO:0006629	lipid metabolic process	0.61	GO:0016788	hydrolase activity, acting on ester bonds	0.33	GO:0016021	integral component of membrane	0.61	EC:3.1.-.-	GO:0016788
Lus10035595	0.79	IQ domain-containing protein IQM3-like				0.34	GO:0016874	ligase activity	0.60	GO:0005634	nucleus	0.34	EC:6.-.-.-	GO:0016874
Lus10035591	0.63	Serine/threonine-protein kinase KIPK	0.64<br>0.38	GO:0006468<br>GO:0009638	protein phosphorylation<br>phototropism	0.64<br>0.55	GO:0004672<br>GO:0005524	protein kinase activity<br>ATP binding	0.43<br>0.40<br>0.39	GO:0005634<br>GO:0005886<br>GO:0005737	nucleus<br>plasma membrane<br>cytoplasm			
Lus10038763	0.80	BTB/POZ domain-containing protein At5g47800 isoform X2	0.71<br>0.35	GO:0016567<br>GO:0043086	protein ubiquitination<br>negative regulation of catalytic activity	0.38<br>0.35	GO:0005509<br>GO:0004857	calcium ion binding<br>enzyme inhibitor activity						
Lus10025454	0.82	homeobox-DDT domain protein RLT1 isoform X1	0.68<br>0.66<br>0.46	GO:0006357<br>GO:2000112<br>GO:0015713	regulation of transcription by RNA polymerase II<br>regulation of cellular macromolecule biosynthetic process<br>phosphoglycerate	0.56<br>0.46<br>0.44	GO:0003677<br>GO:0015120<br>GO:0015605	DNA binding<br>phosphoglycerate transmembrane transporter activity<br>organophosphate ester transmembrane transporter activity	0.59<br>0.43<br>0.35	GO:0005634<br>GO:0031969<br>GO:0016021	nucleus<br>chloroplast membrane<br>integral component of membrane			

			0.43	GO:0015748	transmembrane transport<br>organophosphate ester transport									
Lus10019044	0.74	Zinc finger CCCH domain-containing protein 53				0.58<br>0.54<br>0.52	GO:0003723<br>GO:0046872<br>GO:0003677	RNA binding<br>metal ion binding<br>DNA binding						
Lus10030872	0.92	probable cation transporter HKT7	0.71<br>0.61<br>0.39<br>0.37	GO:0006813<br>GO:0098655<br>GO:0006814<br>GO:0098660	potassium ion transport<br>cation transmembrane transport<br>sodium ion transport<br>inorganic ion transmembrane transport	0.62<br>0.37	GO:0008324<br>GO:0015318	cation transmembrane transporter activity<br>inorganic molecular entity transmembrane transporter activity	0.44<br>0.40	GO:0016021<br>GO:0005886	integral component of membrane<br>plasma membrane			
Lus10030865	0.0	Uncharacterized protein												
Lus10030875	0.84	AMSH-like ubiquitin thioesterase 2 isoform X1	0.84	GO:0070536	protein K63-linked deubiquitination	0.85<br>0.80<br>0.67<br>0.46	GO:0061578<br>GO:0070122<br>GO:0008237<br>GO:0004843	Lys63-specific deubiquitinase activity<br>isopeptidase activity<br>metallopeptidase activity<br>thiol-dependent deubiquitinase	0.44<br>0.35<br>0.35	GO:0005768<br>GO:0000502<br>GO:0016020	endosome<br>proteasome complex<br>membrane	0.46	EC:3.4.19.12	GO:0004843
Lus10026287	0.51	5'-methylthioadenosine/S-adenosylhomocysteine nucleosidase 2	0.78<br>0.71	GO:0019509<br>GO:0009116	L-methionine salvage from methylthioadenosine<br>nucleoside metabolic process	0.83<br>0.36	GO:0008930<br>GO:0008782	methylthioadenosine nucleosidase activity<br>adenosylhomocysteine nucleosidase activity				0.83	EC:3.2.2.16	GO:0008930
Lus10026294	0.48	Auxin-induced SAUR	0.78<br>0.35<br>0.35	GO:0009733<br>GO:0040008<br>GO:0009755	response to auxin<br>regulation of growth<br>hormone-mediated signaling pathway				0.33	GO:0005886	plasma membrane			
Lus10026296	0.52	Auxin-induced protein X15	0.78<br>0.35<br>0.35	GO:0009733<br>GO:0040008<br>GO:0009755	response to auxin<br>regulation of growth<br>hormone-mediated signaling pathway									
Lus10026284	0.71	Alkaline/neutral invertase	0.85<br>0.38<br>0.35	GO:0005987<br>GO:0006621<br>GO:0015031	sucrose catabolic process<br>protein retention in ER lumen<br>protein transport	0.85<br>0.85<br>0.38	GO:0004575<br>GO:0033926<br>GO:0046923	sucrose alpha-glucosidase activity<br>glycopeptide alpha-N-acetylgalactosaminidase activity<br>ER retention sequence binding	0.36<br>0.32	GO:0005789<br>GO:0016021	endoplasmic reticulum membrane<br>integral component of membrane	0.85<br>0.85	EC:3.2.1.48<br>KEGG:R00802	GO:0004575<br>GO:0004575
Lus10026281	0.78	Proline-rich protein 4	0.59<br>0.57	GO:0000413<br>GO:0006457	protein peptidyl-prolyl isomerization<br>protein folding	0.60	GO:0003755	peptidyl-prolyl cis-trans isomerase activity	0.56	GO:0005576	extracellular region	0.60	EC:5.2.1.8	GO:0003755
Lus10010522	0.51	Chromatin remodeling complex subunit isoform 1	0.48<br>0.45<br>0.35	GO:0045944<br>GO:2000112<br>GO:0032508	positive regulation of transcription by RNA polymerase II<br>regulation of cellular macromolecule biosynthetic process<br>DNA duplex	0.77<br>0.55<br>0.55<br>0.46<br>0.41<br>0.35	GO:0042393<br>GO:0005524<br>GO:0004386<br>GO:0008094<br>GO:0003677<br>GO:0016787	histone binding<br>ATP binding<br>helicase activity<br>ATP-dependent activity, acting on DNA<br>DNA binding<br>hydrolase activity	0.44	GO:0005634	nucleus	0.35	EC:3.-.-.-	GO:0016787

		unwinding			
Lus10010523	0.80 Ran GTPase activating protein 2	0.46 GO:0006955 immune response<br>0.45 GO:0098542 defense response to other organism<br>0.34 GO:0006412 translation	0.35 GO:0003735 structural constituent of ribosome	0.60 GO:0005634 nucleus<br>0.34 GO:0005840 ribosome<br>0.33 GO:0005737 cytoplasm<br>0.32 GO:0016021 integral component of membrane	
Lus10021892	0.49 Non-specific serine/threonine protein kinase	0.63 GO:0006468 protein phosphorylation<br>0.59 GO:0007165 signal transduction<br>0.39 GO:0009409 response to cold	0.72 GO:0106310 protein serine kinase activity<br>0.71 GO:0004712 protein serine/threonine/tyrosine kinase activity<br>0.69 GO:0004674 protein serine/threonine kinase activity<br>0.55 GO:0005524 ATP binding		0.71 EC:2.7.12.1 GO:0004712
Lus10021903	0.0 Uncharacterized protein				
Lus10021899	0.49 Mitogen-activated protein kinase kinase kinase YODA	0.63 GO:0006468 protein phosphorylation<br>0.48 GO:0018210 peptidyl-threonine modification<br>0.47 GO:0050832 defense response to fungus<br>0.47 GO:0018209 peptidyl-serine modification<br>0.36 GO:0006955 immune response	0.63 GO:0004672 protein kinase activity<br>0.55 GO:0005524 ATP binding	0.44 GO:0016021 integral component of membrane	
Lus10021902	0.80 1,2-dihydroxy-3-keto-5-methylthiopentene dioxygenase	0.77 GO:0019509 L-methionine salvage from methylthioadenosine	0.84 GO:0010309 acireductone dioxygenase [iron(II)-requiring] activity<br>0.66 GO:0005506 iron ion binding	0.59 GO:0005634 nucleus<br>0.51 GO:0005737 cytoplasm	0.84 EC:1.13.11.54 GO:0010309<br>0.84 KEGG:R07364 GO:0010309
Lus10031976	0.55 LRR receptor-like serine/threonine-protein kinase RPK2	0.64 GO:0006468 protein phosphorylation<br>0.36 GO:0050832 defense response to fungus<br>0.35 GO:0051726 regulation of cell cycle<br>0.35 GO:0000165 MAPK cascade	0.64 GO:0004672 protein kinase activity<br>0.56 GO:0005524 ATP binding<br>0.39 GO:0032440 2-alkenal reductase [NAD(P)+] activity	0.44 GO:0016021 integral component of membrane<br>0.34 GO:0005886 plasma membrane	0.39 EC:1.3.1.74 GO:0032440
Lus10031972	0.92 COBRA-like protein 4	0.85 GO:0010215 cellulose microfibril organization<br>0.54 GO:0052324 plant-type cell wall cellulose biosynthetic process<br>0.42 GO:0009834 plant-type secondary cell wall biogenesis		0.77 GO:0031225 anchored component of membrane<br>0.44 GO:0031226 intrinsic component of plasma membrane<br>0.41 GO:0016021 integral component of membrane<br>0.36 GO:0000325 plant-type vacuole	
Lus10014757	0.61 LOB domain-containing protein	0.76 GO:0009755 hormone-mediated signaling pathway<br>0.72 GO:0045893 positive regulation of transcription, DNA-templated<br>0.67 GO:2000112 regulation of cellular		0.60 GO:0005634 nucleus	

					macromolecule biosynthetic process									
Lus10023056	0.54	Cellulose synthase-like protein G3	0.80<br>0.53<br>0.48	GO:0030244<br>GO:0009833<br>GO:0097502	cellulose biosynthetic process<br>plant-type primary cell wall biogenesis<br>mannosylation	0.82<br>0.53	GO:0016760<br>GO:0051753	cellulose synthase (UDP-forming) activity<br>mannan synthase activity	0.44<br>0.39	GO:0016021<br>GO:0005886	integral component of membrane<br>plasma membrane	0.82	EC:2.4.1.12	GO:0016760
Lus10023055	0.97	probable E3 ubiquitin-protein ligase RHA4A	0.51<br>0.50<br>0.35	GO:0016567<br>GO:0006511<br>GO:0008643	protein ubiquitination<br>ubiquitin-dependent protein catabolic process<br>carbohydrate transport	0.52<br>0.36<br>0.34<br>0.33<br>0.33	GO:0061630<br>GO:0016874<br>GO:0016746<br>GO:0046872<br>GO:0016787	ubiquitin protein ligase activity<br>ligase activity<br>acyltransferase activity<br>metal ion binding<br>hydrolase activity	0.44	GO:0016021	integral component of membrane	0.36	EC:6.-.-.-	GO:0016874
Lus10023049	0.49	Plant regulator RWP-RK family protein	0.67<br>0.58<br>0.37<br>0.37<br>0.36<br>0.35	GO:2000112<br>GO:0006355<br>GO:0010118<br>GO:0010167<br>GO:0009414<br>GO:0042128	regulation of cellular macromolecule biosynthetic process<br>regulation of transcription, DNA-templated<br>stomatal movement<br>response to nitrate<br>response to water deprivation<br>nitrate assimilation	0.62<br>0.40	GO:0003700<br>GO:0000976	DNA-binding transcription factor activity<br>transcription cis-regulatory region binding	0.35	GO:0005634	nucleus			
Lus10027958	0.0	Uncharacterized protein												
