# Supplementary material for: Identification of QTNs Associated With Flowering Time, Maturity, and Plant Height Traits in Linum usitatissimum L. Using Genome-Wide Association Study
Source: Front Genet. 2022 Jun 14;13:811924. doi: 10.3389/fgene.2022.811924 (PMC9237403; doi:10.3389/fgene.2022.811924)
Supplement: Supplementary file 12 [file Table3.DOCX]

**Supplementary table S3: Distribution of identified QTNs across methods/environments/traits.**

**(A) Distribution of QTNs across methods and environments:**

| **Method** | **AK1819** | **AK1920** | **DL1718** | **DL1819** | **DL1920** | **TOTAL** |
| --- | --- | --- | --- | --- | --- | --- |
| **FASTmrEMMA** | 7 (7) | 8 (8) | 6 (6) | 4 (4) | 3 (3) | **28 (26)** |
| **FASTmrMLM** | 17 (15) | 25 (22) | 23 (22) | 19 (17) | 27 (25) | **111 (94)** |
| **ISIS EM-BLASSO** | 37 (35) | 32 (28) | 36 (34) | 32 (29) | 31 (26) | **168 (144)** |
| **mrMLM** | 23 (20) | 36 (31) | 27 (26) | 26 (26) | 23 (21) | **135 (115)** |
| **pLARmEB** | 42 (39) | 36 (29) | 35 (34) | 28 (26) | 37 (35) | **178 (149)** |
| **TOTAL** | **126 (82)** | **137 (76)** | **127 (77)** | **109 (79)** | **121 (68)** | **620 (335)** |

*Number of QTNs in the parentheses are unique QTNs after removing trait redundancy.

**(B) Distribution of QTNs across traits and environments:**

| **Environment** | **DF5** | **DF50** | **DF95** | **DM** | **PH** | **TOTAL** |
| --- | --- | --- | --- | --- | --- | --- |
| **AK1819** | 24 (15) | 20 (18) | 19 (15) | 39 (28) | 24 (18) | **126 (82)** |
| **AK1920** | 36 (21) | 40 (23) | 26 (16) | 13 (10) | 22 (20) | **137 (76)** |
| **DL1718** | 14 (9) | 30 (18) | 25 (17) | 33 (21) | 25 (17) | **127 (77)** |
| **DL1819** | 23 (18) | 17 (14) | 27 (19) | 20 (16) | 22 (20) | **109 (79)** |
| **DL1920** | 17 (12) | 20 (14) | 20 (11) | 29 (15) | 35 (25) | **121 (68)** |
| **TOTAL** | **114 (68)** | **127 (80)** | **117 (76)** | **134 (84)** | **128 (95)** | **620 (335)** |

*Number of QTNs in the parentheses are unique QTNs after removing method redundancy.

**(C) Distribution of QTNs across methods and traits:**

| **Method** | **DF5** | **DF50** | **DF95** | **DM** | **PH** | **TOTAL** |
| --- | --- | --- | --- | --- | --- | --- |
| **FASTmrEMMA** | 4 (4) | 7 (6) | 1 (1) | 7 (7) | 9 (9) | **28 (26)** |
| **FASTmrMLM** | 19 (18) | 23 (22) | 22 (22) | 22 (21) | 25 (25) | **111 (94)** |
| **ISIS EM-BLASSO** | 30 (28) | 37 (37) | 34 (33) | 36 (36) | 31 (31) | **168 (144)** |
| **mrMLM** | 28 (26) | 26 (25) | 24 (23) | 31 (31) | 26 (26) | **135 (115)** |
| **pLARmEB** | 33 (29) | 34 (32) | 36 (35) | 38 (35) | 37 (37) | **178 (149)** |
| **TOTAL** | **114 (68)** | **127 (80)** | **117 (76)** | **134 (84)** | **128 (95)** | **620 (335)** |

*Number of QTNs in the parentheses are unique QTNs after removing environment redundancy.
